# Global, regional, and national estimates of pneumonia morbidity and mortality in children younger than 5 years between 2000 and 2015: a systematic analysis

**DOI:** 10.1016/S2214-109X(18)30408-X

**Published:** 2018-11-26

**Authors:** David A McAllister, Li Liu, Ting Shi, Yue Chu, Craig Reed, John Burrows, Davies Adeloye, Igor Rudan, Robert E Black, Harry Campbell, Harish Nair

**Affiliations:** a Institute of Health and Wellbeing, University of Glasgow, Glasgow, UK; b Department of International Health, Institute for International Programs, Baltimore, MD, USA; c Department of Population, Family and Reproductive Health, Baltimore, MD, USA; d Johns Hopkins Bloomberg School of Public Health, Baltimore, MD, USA; e Centre for Global Health Research, Usher Institute of Population Health Sciences and Informatics, University of Edinburgh, Edinburgh, UK; f Global Health Research Institute, Lagos, Nigeria; g Public Health Foundation of India, Gurgaon, India

## Abstract

**Background:**

Global child mortality reduced substantially during the Millennium Development Goal period (2000–15). We aimed to estimate morbidity, mortality, and prevalence of risk factors for child pneumonia at the global, regional, and national level for developing countries for the Millennium Development Goal period.

**Methods:**

We estimated the incidence, number of hospital admissions, and in-hospital mortality due to all-cause clinical pneumonia in children younger than 5 years in developing countries at 5-year intervals during the Millennium Development Goal period (2000–15) using data from a systematic review and Poisson regression. We estimated the incidence and number of cases of clinical pneumonia, and the pneumonia burden attributable to HIV for 132 developing countries using a risk-factor-based model that used Demographic and Health Survey data on prevalence of the various risk factors for child pneumonia. We also estimated pneumonia mortality in young children using data from multicause models based on vital registration and verbal autopsy.

**Findings:**

Globally, the number of episodes of clinical pneumonia in young children decreased by 22% from 178 million (95% uncertainty interval [UI] 110–289) in 2000 to 138 million (86–226) in 2015. In 2015, India, Nigeria, Indonesia, Pakistan, and China contributed to more than 54% of all global pneumonia cases, with 32% of the global burden from India alone. Between 2000 and 2015, the burden of clinical pneumonia attributable to HIV decreased by 45%. Between 2000 and 2015, global hospital admissions for child pneumonia increased by 2·9 times with a more rapid increase observed in the WHO South-East Asia Region than the African Region. Pneumonia deaths in this age group decreased from 1·7 million (95% UI 1·7–2·0) in 2000 to 0·9 million (0·8–1·1) in 2015. In 2015, 49% of global pneumonia deaths occurred in India, Nigeria, Pakistan, Democratic Republic of the Congo, and Ethiopia collectively. All key risk factors for child pneumonia (non-exclusive breastfeeding, crowding, malnutrition, indoor air pollution, incomplete immunisation, and paediatric HIV), with the exception of low birthweight, decreased across all regions between 2000 and 2015.

**Interpretation:**

Globally, the incidence of child pneumonia decreased by 30% and mortality decreased by 51% during the Millennium Development Goal period. These reductions are consistent with the decrease in the prevalence of some of the key risk factors for pneumonia, increasing socioeconomic development and preventive interventions, improved access to care, and quality of care in hospitals. However, intersectoral action is required to improve socioeconomic conditions and increase coverage of interventions targeting risk factors for child pneumonia to accelerate decline in pneumonia mortality and achieve the Sustainable Development Goals for health by 2030.

**Funding:**

Bill & Melinda Gates Foundation.

## Introduction

Global child mortality, including pneumonia mortality, has decreased substantially since 2000; however, mortality remains high: an estimated 921 000 children younger than 5 years died of pneumonia in 2015.^1^ In addition to monitoring mortality, it is important to monitor the underlying incidence of childhood pneumonia to inform preventive intervention strategies and health service planning, because the burden of pneumonia on both in-patient and out-patient health-care services is substantial. We previously published the first global estimates of clinical pneumonia incidence for young children for the year 2000 (155·8 million cases),^2^ and subsequently updated these estimates for 2010 (120·4 million cases).^3^ However, data on HIV and measles immunisation status (two key risk factors for pneumonia in young children) were not included in the model used to estimate clinical pneumonia episodes at the country level.^4^ We have since developed an improved risk-factor-based model incorporating HIV and measles immunisation data and have reported that the overall global pneumonia morbidity estimates from this new model are consistent with those reported using the previous approach.^5^ We also reported the first global estimates of clinical pneumonia episodes in young children with HIV in 2010 (approximately 1·4 million episodes), of which 86% of cases were attributable to HIV.^5^ We have also reported the first global and regional 2010 estimates for hospital admissions due to pneumonia in young children (11·9 million admissions).^6^

**Research in context**

**Evidence before this study**

In 2000, we published global estimates for clinical pneumonia (155·8 million cases) and severe pneumonia (13·1 million cases), and pneumonia deaths (2·1 million deaths) in children younger than 5 years. We subsequently updated these estimates for 2010 (clinical pneumonia [120·4 million cases] and severe pneumonia [14·1 million cases]) and published the first estimates of pneumonia episodes attributable to HIV (1·4 million cases) and hospital admissions for pneumonia (11·9 million admissions) in young children (<5 years) in 2010. We have also published 2015 estimates of pneumonia deaths (921 000 deaths) in young children (<5 years). The Institute for Health Metrics and Evaluation (IHME) estimated 101·8 million episodes of pneumonia (95% uncertainty interval [UI] 90·0–114·4) and 0·7 million pneumonia deaths (95% UI 0·7–0·8) in this age group in 2015 and reported a 37% decrease in morbidity and mortality between 2005 and 2015. The IHME also estimated that globally, 8·9% of the decrease in disability-adjusted life-years lost due to lower respiratory infections in children younger than 5 years during this period was attributable to reductions in childhood malnutrition and 4·3% to decreased exposure to air pollution.

**Added value of this study**

We present updated national estimates of child pneumonia morbidity and mortality for the Millennium Development Goal period (2000–15). We incorporate new data from ten studies reporting incidence of clinical pneumonia and 30 studies reporting hospital admission rates due to pneumonia in young children, and use improved methods to yield a valid time-series analysis. We also provide an in-depth analysis of the seven key risk factors for child pneumonia, and assess the relative contribution of these (particularly HIV) to changes in pneumonia burden.

**Implications of all the available evidence**

Our systematic analysis provides the most up-to-date estimates of morbidity and mortality for child pneumonia in the Millennium Development Goal period. These estimates update previously published estimates and highlight global progress towards reducing the morbidity and mortality from child pneumonia and identify areas that require targeted interventions (especially reducing the prevalence of risk factors for child pneumonia) and can be used to inform national child health intervention strategies.

In this paper, we provide updated estimates of the incidence of all-cause clinical and severe pneumonia and the morbidity attributable to HIV in young children during the Millennium Development Goal period (2000–15) with estimates for both 2000 and 2015. We provide global estimates, overall estimates for developing countries, and country-level estimates for 132 developing countries with high pneumonia mortality. We also estimate the burden of hospital admissions due to pneumonia and pneumonia mortality for 2000 and 2015.

## Methods

### Search strategy and selection criteria

We did a systematic literature review with various search terms. We searched MEDLINE, Embase, Global Health, and LILACS for community-based studies published between Jan 1, 1980, and Dec 31, 2016. Full search terms are provided in the appendix. We also manually searched reference lists of included papers to identify any additional studies not identified using our search strategy. We also searched three Chinese language databases (China National Knowledge Infrastructure, Wanfang, and Chongqing VIP) for studies published in Chinese. We included studies that reported the incidence of WHO-defined clinical pneumonia^7^ (defined as cough or difficulty breathing with tachypnoea) done in developing countries (classified according to UNICEF definitions^8^). We defined low-income and middle-income countries on the basis of the World Bank classification.^9^ We included countries that reported high pneumonia mortality in our previous estimates. Countries without Demographic and Health Surveys (DHS) data were excluded. Full eligibility criteria for the systematic analysis are shown in the panel.

Panel **Eligibility criteria for studies included in the systematic analysis**

**Inclusion criteria**

• Studies published between Jan 1, 1980, and Dec 31, 2016, which reported acute lower respiratory infections among children in developing countries
• Studies reporting incidence of clinical pneumonia in children aged younger than 5 years or the number of episodes of clinical pneumonia with a clear denominator population at risk in developing countries
• Studies reporting number of hospital admissions for pneumonia in children aged younger than 5 years or number of hospital admissions for pneumonia in this age group with a clear denominator population at risk (well defined catchment population)

**Exclusion criteria**

*Clinical pneumonia*

• Studies that are not community-based longitudinal studies
• Studies that do not estimate the incidence of clinical pneumonia
• Studies that are not community-based surveillance of a predefined population of children
• Studies not reporting data for a minimum of 12 consecutive months (or multiples thereof)
• Studies without active case detection
• Studies with surveillance intervals longer than 2 weeks
• Studies with case definitions not clearly defined and consistently applied

*Pneumonia treated in hospital*

• Studies not reporting data for a minimum of 12 consecutive months (or multiples thereof)
• Studies without a clear description of the methods for estimating denominator population
• Studies not reporting data for all-cause pneumonia
• Studies reporting only aetiology specific or chest radiograph confirmed pneumonia
• Studies with case definitions not clearly defined and consistently applied
• Studies reporting hospital admissions using modelling techniques

### Data analysis

DA and IR independently extracted incidence data, and JB and TS independently extracted hospital admission data. Any discrepancies regarding the inclusion of studies or data extraction were resolved by HN. TS searched and extracted data from the Chinese literature. If more than one publication from the same study was identified, we included the paper that reported data for the longest study duration. We estimated the predicted incidence rates of clinical pneumonia in developing countries at 5-year intervals for the period 2000–15 using Poisson regression. We used Markov chain Monte Carlo (MCMC) sampling within the JAGS software package^10^ because this approach allowed us to readily incorporate the uncertainty in these estimates into our previous risk-factor-based pneumonia morbidity model for country specific estimates.^5^ From all clinical pneumonia studies, we identified a subset of studies that reported the proportion of clinical pneumonia cases with lower chest wall indrawing (to estimate severe pneumonia). Since we expected significant heterogeneity in the data, we did random-effects meta-analyses to estimate the overall proportion for developing countries, which was applied to the estimated incidence of clinical pneumonia to estimate the incidence of severe pneumonia at each 5-year timepoint between 2000 and 2015. We used our pneumonia morbidity model to estimate the incidence of clinical pneumonia and severe pneumonia in developing countries.^5^ We stratified all developing countries into WHO subregions on the basis of child and adult mortality data.^11^ We used individual participant-level data on all key risk factors for child pneumonia (low birthweight, crowding, malnutrition, non-exclusive breastfeeding, indoor air pollution, incomplete immunisation, and paediatric HIV)^4^ from DHS, with the exception of data on prevalence of paediatric (<5 years) HIV, which were obtained from UNAIDS, for all developing countries except China (data obtained from the National Bureau of Statistics of China) in the years closest to years 2000 and 2015 to calculate the proportion of participants with each combination of risk factors. If country-level prevalence data were not available for any risk factor, we imputed the median prevalence for the subregion. We used the model to calculate country-specific incidence rates (appendix). In our models, we assumed that the incidence of pneumonia in children without these seven risk factors would be similar within a region, risk ratios could be multiplied when two or more risk factors were present, and risk factors were independently distributed within countries. We also calculated the incidence of clinical pneumonia in children with HIV and incidence of pneumonia attributable to HIV.

We also updated our 2013 review^6^ reporting the number of hospital admissions due to child pneumonia (on the basis of physician's diagnosis and judgment that hospital care was indicated) and in-hospital case fatality ratio (hCFR) and included any studies published between April 1, 2012, and Dec 31, 2016, which satisfied our eligibility criteria (panel). We used Poisson regression with MCMC sampling in JAGS (10 000 samples) to estimate the predicted rate of hospital admissions and hCFR at 5-year intervals between 2000 and 2015. Since we had substantial data for all WHO regions for the period 1995–2015, we reported hospital admissions and hCFR estimates by WHO region. We also estimated the proportion of child pneumonia episodes that were treated in hospital (using the number of episodes with lower chest wall indrawing since the denominator for these cases would have been eligible for hospitalisation as per the WHO recommendations that applied at that time).

We also estimated pneumonia mortality burden for 2000–15. The numbers of deaths and mortality due to pneumonia were calculated separately for neonates and children aged 1–59 months. Estimates were derived by applying the estimated age-specific pneumonia mortality fraction to age-specific all-cause number of deaths and mortality rates from the UN Inter-Agency Group for Child Mortality Estimation.^12^ To generate the age-specific pneumonia mortality fractions, we used vital registration data in countries with adequate vital registration systems, vital-registration-based multicause models for countries with inadequate vital registration systems and low under-5 mortality rate (U5MR), and verbal-autopsy-based multicause models for countries with high U5MR. We used post-hoc adjustment to assess the effect of pneumococcal conjugate vaccines and *Haemophilus influenzae* type b vaccine on pneumonia mortality. Detailed methods are available elsewhere.^1^

All statistical analyses were done using R (version 3.2; Vienna, Austria), JAGS (version 3.4), and Stata (version 11.1). This study was done in accordance with the Guidelines for Accurate and Transparent Health Estimates Reporting (GATHER) recommendations (appendix).

### Role of the funding source

The funder had no role in study design, data collection, data analysis, data interpretation, or writing of the report. DAM, LL, and HN had full access to all the data in the study and HN had the final responsibility for the decision to submit for publication.

## Results

**Figure 1:**
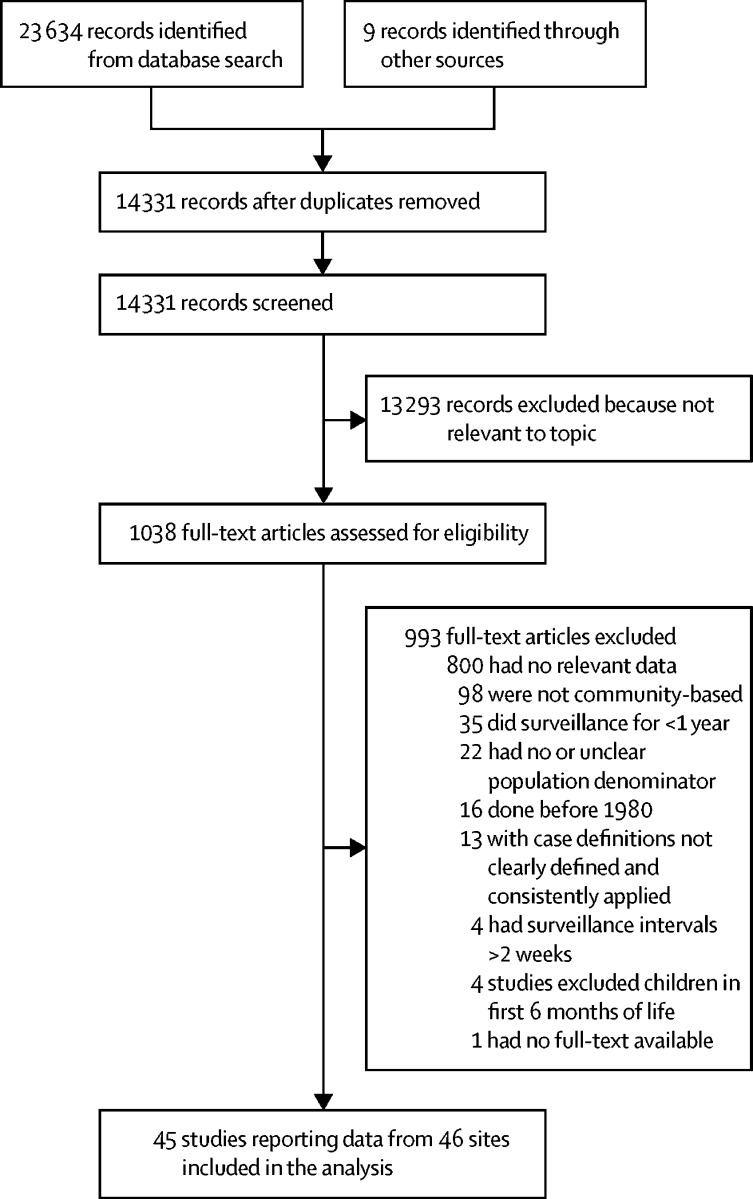
Flow diagram for selection of studies for clinical pneumonia

We identified 45 community-based studies (done between Jan 1, 1980, and Dec 31, 2016) reporting incidence of clinical pneumonia in children younger than 5 years (figure 1; appendix). References for all included studies are listed in the appendix. One study reported implementation of pneumococcal conjugate vaccines and *H influenzae* type b vaccination programmes at study sites during the study period.^13^ 40 (89%) of 45 studies used the WHO Integrated Management of Childhood Illness (IMCI) case definitions for pneumonia.^7^ On the basis of these data, we estimated that the annual incidence of clinical pneumonia in young children in developing countries decreased from 329 episodes per 1000 children (95% UI 201–537) in 2000 to 231 episodes per 1000 children (141–377) in 2015 (appendix). Across developing countries, the number of episodes of clinical pneumonia in children younger than 5 years decreased from 178 million (95% UI 110–289) in 2000 to 138 million (86–226) in 2015, which corresponds to a 30% decrease in incidence and a 22% decrease in number of annual episodes of clinical pneumonia in young children during the 15-year period. We also identified 13 studies (appendix) from developing countries that reported the proportion of cases of clinical pneumonia with lower chest wall indrawing (ie, severe pneumonia). The meta-estimate of the proportion of cases classified as severe was 16% (95% CI 7–39; appendix), which we assumed was fixed across years. This proportion indicates that the number of episodes of severe pneumonia in young children decreased from 28 million (95% UI 18–46) in 2000 to 22 million (14–36) in 2015.

**Figure 2:**
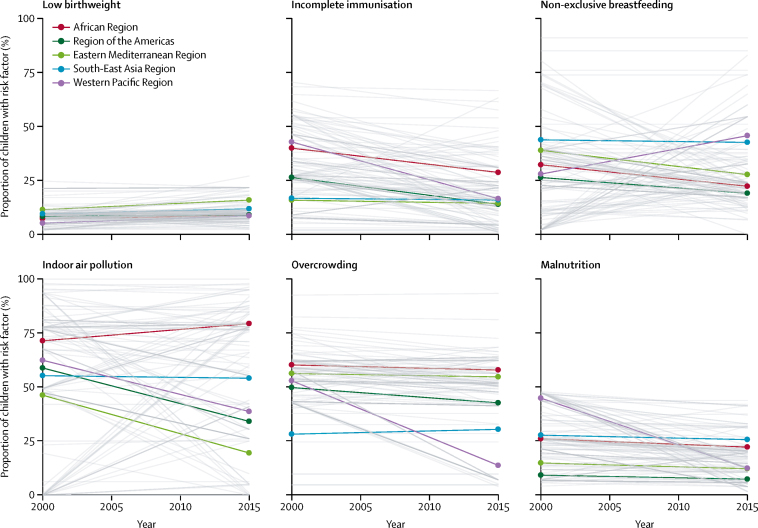
Prevalence of risk factors for child pneumonia (2000–15) Data are from country-level Demographic and Health Surveys for the years closest to 2000 and 2015. Bold coloured lines indicate the proportion of children with the risk factor at the WHO regional level. Grey lines indicate the proportion of children with the risk factor at the country level.

We analysed the difference in prevalence of risk factors for clinical pneumonia at both country and regional levels. We observed that country-level estimates for prevalence of a specific risk factor for clinical pneumonia varied substantially across the DHS. At the regional level, the prevalence of most risk factors decreased between 2000 and 2015 (figure 2). The proportion of children who were malnourished decreased across all regions (ranging from a 7% decrease in the South-East Asia Region to a 72% decrease in the Western Pacific Region), as did the proportion of children with incomplete immunisation (ranging from a 5% decrease in the South-East Asia Region to a 61% decrease in the Western Pacific Region). However, the proportion of newborn babies with a low birthweight increased across all regions between 2000 and 2015 (ranging from a 9% increase in the Region of the Americas to a 67% increase in the Western Pacific region). In 2015, in developing countries, an estimated 317 million (95% UI 316–318) children younger than 5 years were exposed to indoor air pollution, 270 million (269–271) were exposed to overcrowding, and 146 million (145–147) were exposed to non-exclusive breastfeeding (appendix). In the African Region in 2015, 126 million (95% UI 126–127) children younger than 5 years were exposed to indoor air pollution and 935 000 (876 000–994 000) children were exposed to HIV. In the South-East Asia Region, 125 million (95% UI 124–125) children younger than 5 years were exposed to indoor air pollution and this remained unchanged from 2000 (appendix).

**Figure 3:**
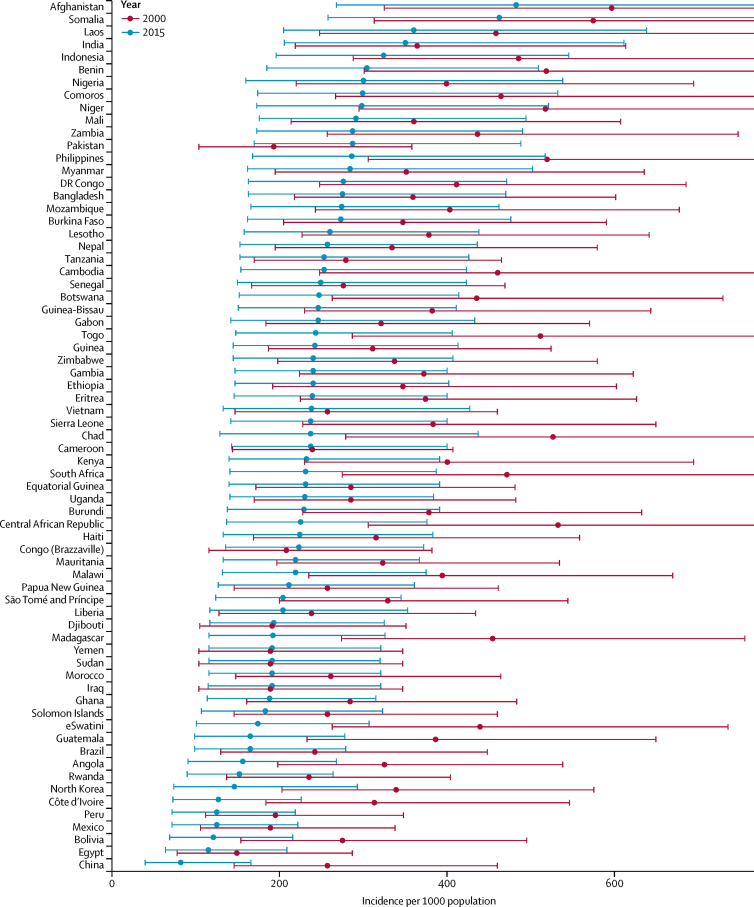
Change in incidence of clinical pneumonia in children younger than 5 years in 132 developing countries between 2000 and 2015 Error bars show 95% uncertainty intervals. 95% uncertainty intervals for some 2000 estimates were wide and the upper limits exceeded the scale used.

In 2000, at the country level, the annual incidence of clinical pneumonia and severe pneumonia in children younger than 5 years ranged from 598 episodes per 1000 children (95**%** UI 325–1122) and 95 episodes per 1000 children (35–233), respectively, in Afghanistan to 151 (78–287) and 24 (8–60), respectively, in Egypt (appendix). In 2000, India, China, Indonesia, Nigeria, and Bangladesh contributed to more than 50% of cases of clinical pneumonia and severe pneumonia in developing countries. In 2015, at the country level, the annual incidence of clinical pneumonia and severe pneumonia ranged from 484 episodes per 1000 children (95% UI 268–895) and 78 episodes per 1000 children (29–188), respectively, in Afghanistan to 84 (40–166) and 14 (5–34), respectively, in China (figure 3; appendix). In 2015, India, Nigeria, Indonesia, Pakistan, and China contributed to more than 54% of the cases of clinical pneumonia and severe pneumonia. The incidence of clinical pneumonia and severe pneumonia decreased by at least 25% in 99 (75%) of 132 developing countries, with the highest reduction observed in China (around 69%; figure 3; appendix). We also estimated that episodes of clinical pneumonia and severe pneumonia decreased by at least 25% in 72 (55%) of 132 developing countries with the highest reductions in China and Malaysia (68–69%; appendix). Our analysis indicates that the incidence of clinical pneumonia and severe pneumonia increased in Pakistan by about 50% with a concomitant rise in the number of episodes by about 75%. In 2000 and 2015, India had the highest number of episodes of clinical pneumonia (32% of all episodes in developing countries) and the number of pneumonia episodes decreased by only 3% during this 15-year period.

Across the 132 developing countries included, episodes of clinical pneumonia in children younger than 5 years with HIV decreased from 3·1 million (95% UI 1·9–5·3) in 2000 to 1·7 million (1·0–2·9) in 2015 (appendix). Overall, episodes of clinical pneumonia attributable to HIV decreased from 2·5 million (95% UI 1·5–4·3) in 2000 to 1·4 million (0·8–2·4) in 2015, which corresponds to a 45% reduction in episodes of clinical pneumonia in children with HIV younger than 5 years during this 15-year period. In 33 (25%) of the 132 developing countries, pneumonia incidence decreased by more than 50% and in 93 (70%) incidence decreased by at least 25% during this period (appendix). In 11 countries, the number of episodes of clinical pneumonia in children with HIV decreased by more than 70%, and 59% of developing countries reported a reduction of at least 33% in the number of pneumonia cases in children with HIV. 14 countries reported an increase in the episodes of clinical pneumonia in children with HIV (ranging from 5% in Nigeria to 104% in Equatorial Guinea). Of these 14 countries, only Nigeria and Mozambique are included in the UNAIDS Global Plan.^14^ The proportionate contribution of sub-Saharan Africa to the global estimates of clinical pneumonia in children with HIV decreased from 78% in 2000 to 72% in 2015.

**Figure 4:**
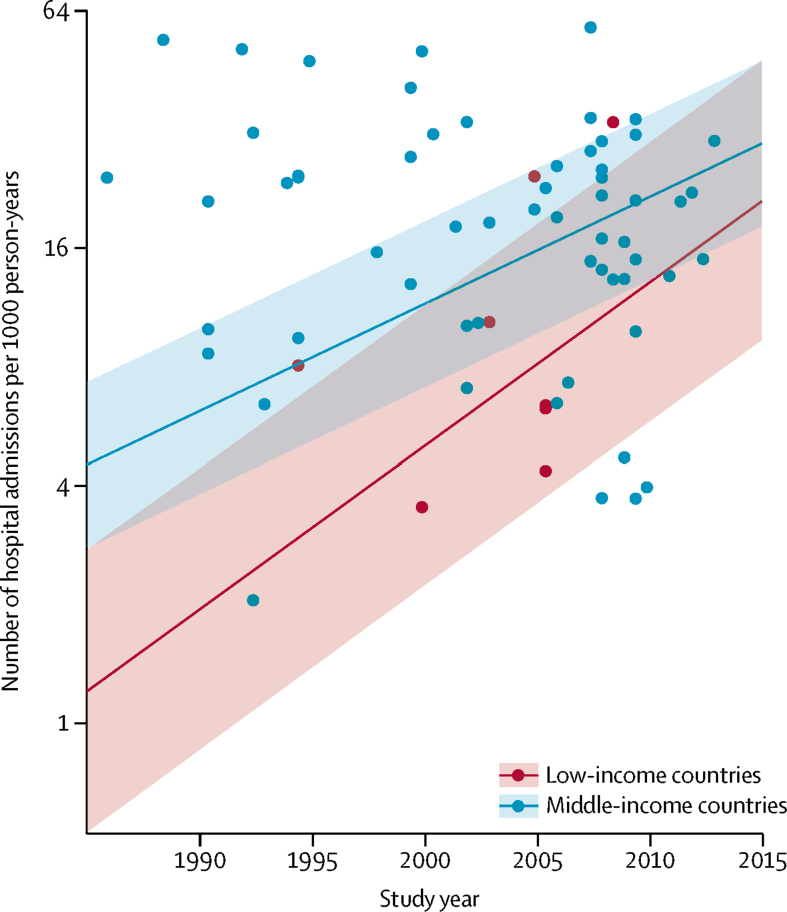
Change in rate of hospital admissions due to pneumonia in low-income and middle-income countries between 1990 and 2015 Shaded bands show 95% uncertainty intervals. Estimates for 2000 were based on data obtained between 1980 and 2000.

**Figure 5:**
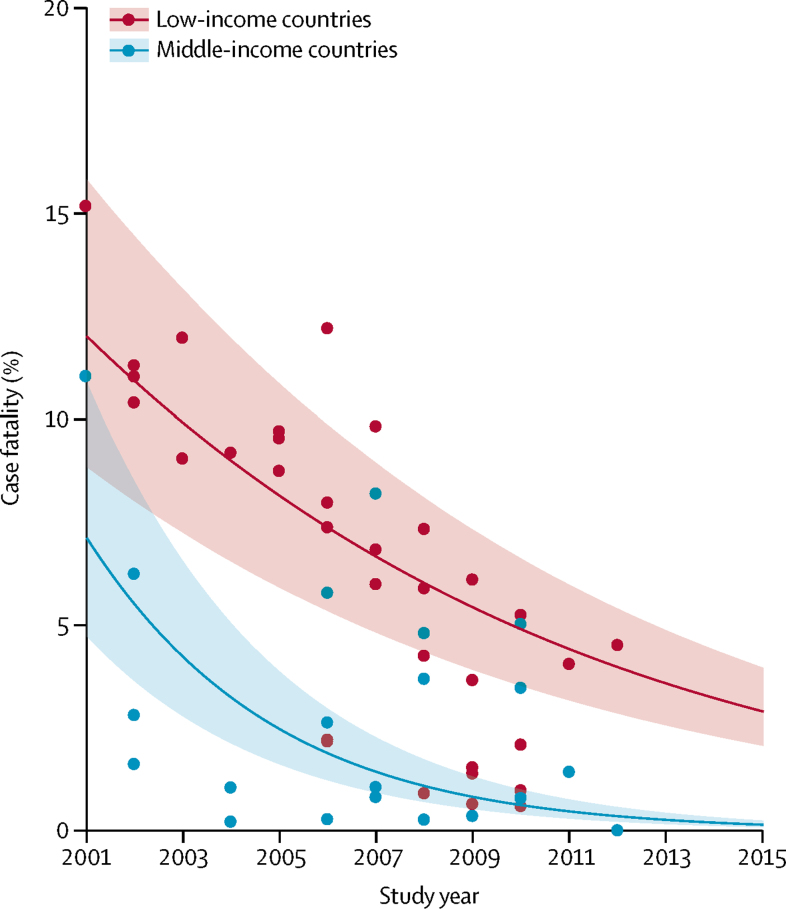
In-hospital case fatality rate for young children admitted to hospital with pneumonia in low-income and middle-income countries between 2001 and 2015 Shaded bands show 95% uncertainty intervals.

We updated our 2013 systematic review of hospital admissions for pneumonia in children younger than 5 years and identified 18 new studies reporting the number of hospital admissions (appendix). During the 15-year period, the number of hospital admissions due to pneumonia in low-income countries increased by 320% compared with around 155% for middle-income countries (figure 4; appendix). The proportion of children with severe pneumonia who were admitted to hospital in low-income countries increased by 7·6 times (from 7% to 50%) and by 3·5 times (from 23% to 80%) in middle-income countries in this 15-year period. The increase in hospital admissions was higher in the South-East Asia Region than the African Region (appendix). In low-income and middle-income countries, the number of children admitted to hospital due to pneumonia increased by 187%, from 5·7 million (95% UI 3·5–9·5) in 2000 to 16·4 million (9·8–28·0) in 2015 (appendix). Overall, hospital admissions for child pneumonia increased by 2·9 times during the 15-year period. We also identified 27 new studies reporting hCFR for hospital admissions due to pneumonia (appendix) and observed a substantial decline in hCFR during this period (figure 5). The decline was faster in the South-East Asia Region than the African Region (appendix).

Globally, the number of deaths due to pneumonia among children younger than 5 years was 0·9 million (95% UI 0·8–1·1) in 2015, with more than 80% of deaths occurring in those aged 1–59 months (0·8 million [0·7–0·9]; appendix). Between 2000 and 2015, the burden of pneumonia deaths decreased substantially. Specifically, the number of deaths due to pneumonia decreased by nearly half, from 1·7 million (95% UI 1·7–2·0) in 2000 to 0·9 million (0·8–1·1) in 2015 (appendix). Globally, pneumonia-specific mortality among children younger than 5 years decreased from 13·6 per 1000 livebirths (95% UI 12·9–15·4) in 2000 to 6·6 per 1000 livebirths (5·8–8·0) in 2015, with the largest contribution to total decrease in U5MR among all causes. In developing countries, pneumonia-specific mortality decreased from about 15·2 per 1000 livebirths (95% UI 14·3–17·3) in 2000 to about 7·4 per 1000 livebirths (6·7–8·8) in 2015. The CFR for clinical pneumonia decreased from 0·96% in 2000 to 0·65% in 2015 and from 6·1% to 4·2% for severe pneumonia during the same period.

In 2015, the WHO African Region had the highest burden of pneumonia deaths (0·5 million [95% UI 0·4–0·6]) among children younger than 5 years, followed by the South-East Asia Region (0·2 million [0·2–0·3]). Taken together, the two regions accounted for more than three-quarters of global pneumonia deaths in children younger than 5 years. The five countries with the highest number of pneumonia deaths in children younger than 5 years were India, Nigeria, Pakistan, Democratic Republic of the Congo, and Ethiopia (appendix), accounting for 49% of global pneumonia deaths collectively. The five countries with the highest pneumonia mortality rates were Somalia, Chad, Angola, Central African Republic, and Niger (appendix).

## Discussion

We report regional-level and country-level estimates for clinical pneumonia and hospital admissions due to pneumonia in young children (aged <5 years) during a 15-year period (2000–15) using a time-trend analysis. Globally, the incidence of clinical pneumonia in young children decreased by 30% and the number of episodes of clinical pneumonia decreased by 22% during this period. The number of episodes of clinical pneumonia in children with HIV decreased by 45% in this age group, with an increasing proportion of episodes occurring in non-African countries. Data from hospital-based studies indicate a 187% increase in hospital admissions due to child pneumonia between 2000 and 2015. Additionally, pneumonia mortality decreased by 51% in this age group during the 15-year period.

Our current morbidity estimates used improved methods compared with previous estimates, such as the inclusion of data from Chinese language databases, a model based on seven risk factors instead of five, Poisson regression with MCMC sampling in JAGS to report uncertainty ranges, use of individual-level data rather than aggregate data from large-scale surveys, and the assumption that risk factors were independently distributed rather than mutually exclusive. We used these methods to recalculate the 2000–15 estimates to yield a valid time-series analysis. The new estimates are thus not directly comparable with our previous 2000 and 2010 estimates for clinical pneumonia or hospital admissions due to pneumonia.^3^, ^6^ Our pneumonia morbidity and mortality estimates are also higher than those reported by the 2015 Global Burden of Disease Study.^15^ Those authors reported global estimates of 101·8 million (95% UI 90·0–114·4) episodes of pneumonia and 0·7 million (0·7–0·8) pneumonia deaths in this age group with a 37% decrease in morbidity and mortality between 2005 and 2015. In 2015, we published a review^16^ comparing the methods used by our group and the Global Burden of Disease group and identified a number of key differences. Our group used UN Population Division census estimates on number of children nationally and globally and the UN Inter-Agency Group on Mortality Estimation estimates on numbers of child deaths to promote global consensus and consistency, which are not currently used by the Institute for Health Metrics and Evaluation. For cause of death modelling within child deaths, our group used stricter inclusion criteria (requiring higher levels of completeness and representativeness to minimise selection biases) for verbal autopsy and vital registration data and attempted to avoid using data from high-income countries to estimate cause of death patterns in low-income and middle-income settings, which have substantially different epidemiology. We also attributed the neonatal causes differently across pneumonia, sepsis, and meningitis categories. This exercise was a useful first step in attempting to harmonise global estimates. Further progress requires that all research groups adhere to the minimum reporting requirements contained in the GATHER recommendations with increasing willingness to share details of datasets, data processing (including analysis code), and covariates. Ideally, we can move towards a position in which there is agreement on a common set of covariates and modelling groups would be able to access an agreed common dataset.^17^

Several sources of uncertainty exist in our morbidity estimates: case definitions, coverage of pneumococcal conjugate vaccines and *H influenzae* type b vaccine in children, prevalence data for the risk factors for pneumonia, limited data on the proportion of clinical pneumonia that are classified as severe, and assumptions of independence. Our mortality estimates include a post-model adjustment for pneumococcal conjugate vaccines and *H influenzae* type b vaccines in children.^1^ The unadjusted global estimates for child pneumonia deaths in 2000 and 2015 were 1·76 million and 1·09 million, respectively (appendix). In our mortality estimates, we accounted for some sources of uncertainty, such as random sampling error and uncertainty from all-cause mortalities, but did not account for other important sources, including model uncertainty, uncertainty around covariate values, and uncertainty associated with misclassification of causes of deaths, particularly when data from verbal autopsy were used.

About 90% of the included studies used the 2005 WHO-IMCI case definitions for clinical pneumonia.^7^ These case definitions were developed for programmatic purposes for use by community health workers and thus are highly sensitive. These definitions have been widely adopted, understood, and used by national control programmes. Continued adoption of these widely accepted definitions over time provides a secure basis from which to assess trends and provides estimates that can be understood in terms of national control programmes in developing countries since WHO guidelines that recommend the use of these clinical definitions are widely used in these programmes. However, use of these case definitions is likely to lead to overestimation of true pneumonia in most settings due to the relatively low specificity of clinical pneumonia (around 70%) and low prevalence of pneumonia compared with children who have cough and difficult breathing.^18^, ^19^ Moreover, this clinical pneumonia definition, which is based on simple clinical signs, encompasses various lower respiratory infections ranging from bronchiolitis to radiologically confirmed pneumonia.^20^ The use of this case definition has had substantial effect at a programmatic level. On one hand, the high detection rate for pneumonia had resulted in a substantial decline in pneumonia mortality; however, on the other hand, a level of overprescription of antibiotics has been accepted, which is a matter of concern with regard to widespread antimicrobial resistance. Several experts have proposed that cases diagnosed in this way should be defined as WHO pneumonia (based on simple clinical signs) to better distinguish from physician-diagnosed pneumonia. The previous WHO case definition for severe pneumonia is based on lower chest wall indrawing alone, which often overestimates the proportion of children with pneumonia who required referral to hospital. WHO revised the definition for severe pneumonia in 2014,^21^ restricting this to children with signs of pneumonia who also had so-called danger signs, such as reduced conscious level and inability to drink. However, none of the included studies used the revised case definitions. The adoption of these revised case definitions is expected to result in improved pneumonia case management in the community and a lower number of hospital admissions over time.^22^ The effect of these revised guidelines on pneumonia mortality in populations with poor access to health care is uncertain.^23^

Between 2000 and 2015, incidence of both clinical and severe pneumonia decreased by 30%, case fatality rate decreased by 32%, and mortality by 50% for child pneumonia. The decrease in pneumonia incidence during this period is consistent with the decrease in the number of children exposed to some of the key risk factors for pneumonia (HIV, incomplete immunisation [measles vaccination], malnutrition, and overcrowding [figure 2; appendix]). However, increases in the number of children born with a low birthweight, and those exposed to indoor air pollution and non-exclusive breastfeeding (both amenable to low-cost interventions and behavioural change), are a cause of concern and an area for targeted action. We postulate that the reduction in pneumonia mortality is a result of both decrease in incidence, due to reduced risk factor exposure with increasing socioeconomic development and preventive interventions, and a decrease in case fatality rate, which is a result of improved access to care, likely lowering of the threshold for hospital admission, and improved quality of care in hospitals. The decrease in incidence of, and mortality from, childhood pneumonia during this period is a result of interconnected social, economic, political, health system, and health programme factors.^24^ The decrease can be partly attributed to the introduction and scale-up of vaccines against bacterial pneumonia (*H influenzae* type b and *Streptococcus pneumoniae*). Although in 2015, the estimated coverage for three doses of *H influenzae* type b and pneumococcal conjugate vaccines received by infants was 63% and 37%, respectively (with wide variations between regions and within countries),^25^ only one of the 45 community-based studies included in our analysis reported implementation of pneumococcal conjugate vaccines and *H influenzae* type b immunisation in the population. Three doses of *H influenzae* type b and pneumococcal conjugate vaccines individually are estimated to reduce the incidence of clinical pneumonia by 4% and 7%, respectively, and severe pneumonia by 6% and 7%, respectively.^26^ Thus, although we did not adjust our estimates for clinical pneumonia and severe pneumonia for vaccine coverage, we do not think we have overestimated the incidence of clinical pneumonia and severe pneumonia at the global level.

The data on prevalence of the risk factors for pneumonia were obtained from country-level DHS datasets. DHS data have several limitations, including the small number of repeat surveys, poor consistency in prevalence estimates between surveys, and absence of prevalence data on one or more risk factors for some countries. Caution should be taken when interpreting data for the Western Pacific Region because of the small number of countries that contributed to the 2000 DHS. We only used prevalence data from surveys for years closest to 2000 and 2015, and if data were not available for a specific country, we imputed the median prevalence of the risk factor in the WHO subregion. We have also been unable to account for wide intracountry variations in socioeconomic conditions and associated risk factor prevalence in populations residing in middle-income countries, which would account for the high heterogeneity in pneumonia incidence in large countries such as India and China. Furthermore, the absence of independence among risk factors is an important limitation. We modelled the effect of relaxing this assumption for clinical pneumonia using individual-level data from 102 DHS, and found that, since the marginal proportions for each risk factor were relatively low, the impact of the assumption of independence on incidence estimates was low (appendix).

The 187% increase in hospital admissions due to child pneumonia between 2000 and 2015 reflects improving access to hospital care in the public and private sectors. This increase supports the current focus on improving quality of care in hospitals and the efforts of WHO and UNICEF in launching a Network for Improving Quality of Care for Maternal, Newborn and Child Health.^27^ The increase in hospital admissions also underlines the importance of health-care planning to ensure adequate capacity in future and focusing hospital referral and admission of children with the poorest prognosis (such as those with hypoxaemia) who would benefit most from inpatient care so that hospital resources are used efficiently and are not overwhelmed by the increase in patient load.

These disease burden estimates do not include the long-term sequelae that accompany pneumonia episodes in young children. Pneumonia leads to interstitial lung damage and reduced future alveolar growth leading to reduced lung capacity and increased risk of chronic lung disease. The increased risk of restrictive lung disease (such as chronic suppurative lung diseases like bronchiectasis and chronic bronchitis) is well documented in both case-control and large population cohort studies, whereas the risk of obstructive lung disease is less clear at present.^28^ The incidence, hospital admission rate, case fatality rates, and risk factors for child pneumonia are likely to be age dependent. However, most of the included studies did not report results using narrow age bands, which in turn limited our ability to report age-specific estimates that could be used for targeted interventions. A small number of studies^13^, ^29^, ^30^ has shown that children without HIV infection who were exposed to the virus prenatally (HIV-exposed uninfected; HEU) have intermediate disease severity and are less likely to respond to treatment than children with HIV and children who have not been exposed to the virus. However, country-level data on the prevalence of pneumonia in HEU children are not available at present. Moreover, little evidence is available regarding pneumonia mortality in this population of children.^30^, ^31^ Therefore, we did not include HEU as a risk factor in our analysis.

Global funding for child pneumonia more than doubled in the period 2008–2013 (from US$306 million to $663 million), with most funding allocated to south Asia and sub-Saharan Africa, where the majority of child pneumonia deaths occur.^32^ In 2011, 69% of the pneumonia funding was allocated to sub-Saharan Africa. However, the progress in improving care and reducing pneumonia mortality has not been equitable between the two regions. Hospital admissions due to pneumonia increased more rapidly in south Asia than Africa, whereas hCFR decreased more rapidly in south Asia than Africa between 2000 and 2015. The decrease observed in south Asia indicates a marked improvement in access to hospital care, care-seeking behaviour, and quality of hospital care. These reductions are consistent with a 2016 UNICEF report,^33^ which showed that three of five children with signs of pneumonia seek care in south Asia compared with only two of five children in sub-Saharan Africa, where the majority of pneumonia deaths occur. Substantial efforts need to be made in sub-Saharan Africa if further improvements in access to hospital care and reductions in pneumonia mortality are to be achieved.

The rate of decline in child pneumonia mortality needs to accelerate to achieve the Sustainable Development Goals for health by 2030.^34^ Future reductions in pneumonia disease burden will require continued socioeconomic development (with attention to minimising population inequities) together with integrated, multisectoral action to protect (by exclusive breastfeeding and complementary feeding and micronutrient supplementation), prevent (timely immunisation, HIV prevention, cotrimoxazole prophylaxis, reduction of air pollution, and improved handwashing and sanitation) and treat (improved care seeking, treatment and referral, and continued feeding) pneumonia according to strategies advocated in the WHO Global Action Plan against Pneumonia and Diarrhoea.^35^ Intersectoral action will be required to reduce fertility, improve socioeconomic conditions, increase coverage of interventions targeting risk factors for child pneumonia mortality, promote appropriate care seeking, and improve quality of care of health services. Tracking progress will be important to highlight problem areas and gaps and could inform priorities and planning. Improved data on childhood pneumonia episodes, hospital admissions, and deaths in each country obtained from District Health Information Systems, civil registration and vital statistics, and high-quality epidemiological studies are required to accurately track trends, including identification and tracking of inequities at the subnational level. The widespread measurement of selected valid indicators of levels of appropriate care seeking and quality of care from health providers for child pneumonia would help guide priorities and investment to identify problems and achieve targets.

## References

[1] Liu L, Oza S, Hogan D (2016). Global, regional, and national causes of under-5 mortality in 2000–15: an updated systematic analysis with implications for the Sustainable Development Goals. Lancet.

[2] Rudan I, Tomaskovic L, Boschi-Pinto C, Campbell H, WHO Child Health Epidemiology Reference Group (2004). Global estimate of the incidence of clinical pneumonia among children under five years of age. Bull World Health Organ.

[3] Rudan I, O'Brien KL, Nair H (2013). Epidemiology and etiology of childhood pneumonia in 2010: estimates of incidence, severe morbidity, mortality, underlying risk factors and causative pathogens for 192 countries. J Glob Health.

[4] Jackson S, Mathews KH, Pulanic D (2013). Risk factors for severe acute lower respiratory infections in children: a systematic review and meta-analysis. Croat Med J.

[5] Theodoratou E, McAllister DA, Reed C (2014). Global, regional, and national estimates of pneumonia burden in HIV-infected children in 2010: a meta-analysis and modelling study. Lancet Infect Dis.

[6] Nair H, Simoes EA, Rudan I (2013). Global and regional burden of hospital admissions for severe acute lower respiratory infections in young children in 2010: a systematic analysis. Lancet.

[7] WHO (2005). Handbook: IMCI integrated management of childhood illness. http://apps.who.int/iris/bitstream/handle/10665/42939/9241546441.pdf?sequence=1&isAllowed=y.

[8] UNICEF (2014). The state of the world's children 2015: Executive summary. Reimagine the future. Innovation for every child. https://www.unicef.org/publications/files/SOWC_2015_Summary_and_Tables.pdf.

[9] The World Bank World Bank country and lending groups. https://datahelpdesk.worldbank.org/knowledgebase/articles/906519.

[10] Plummer M (2003). JAGS: a program for analysis of bayesian graphical models using Gibbs sampling. https://www.r-project.org/conferences/DSC-2003/Proceedings/Plummer.pdf.

[11] WHO Global Burden of Disease Regions used for WHO-CHOICE analyses. http://www.who.int/choice/demography/regions/en/.

[12] United Nations Inter-agency Group for Child Mortality Estimation (2015). Levels and trends in child mortality: report 2015. https://www.unicef.org/media/files/IGME_Report_Final2.pdf.

[13] le Roux DM, Myer L, Nicol MP, Zar HJ (2015). Incidence and severity of childhood pneumonia in the first year of life in a South African birth cohort: the Drakenstein Child Health Study. Lancet Glob Health.

[14] UNAIDS (2011). Global Plan towards the elimination of new HIV infections among children by 2015 and keeping their mothers alive.

[15] GBD 2015 LRI Collaborators (2017). Estimates of the global, regional, and national morbidity, mortality, and aetiologies of lower respiratory tract infections in 195 countries: a systematic analysis for the Global Burden of Disease Study 2015. Lancet Infect Dis.

[16] Kovacs SD, Mullholland K, Bosch J (2015). Deconstructing the differences: a comparison of GBD 2010 and CHERG's approach to estimating the mortality burden of diarrhea, pneumonia, and their etiologies. BMC Infect Dis.

[17] Stevens GA, Alkema L, Black RE (2016). Guidelines for Accurate and Transparent Health Estimates Reporting: the GATHER statement. Lancet.

[18] WHO (1991). Technical bases for the WHO recommendations on the management of pneumonia in children at first-level health facilities.

[19] Campbell H, El Arifeen S, Hazir T (2013). Measuring coverage in MNCH: challenges in monitoring the proportion of young children with pneumonia who receive antibiotic treatment. PLoS Med.

[20] Cherian T, Mulholland EK, Carlin JB (2005). Standardized interpretation of paediatric chest radiographs for the diagnosis of pneumonia in epidemiological studies. Bull World Health Organ.

[21] WHO (2014). Integrated management of childhood illness. Chart booklet.

[22] Black RE, El Arifeen S (2012). Community-based treatment of severe childhood pneumonia. Lancet.

[23] Mulholland K (2018). Problems with the WHO guidelines for management of childhood pneumonia. Lancet Glob Health.

[24] Feng XL, Theodoratou E, Liu L (2012). Social, economic, political and health system and program determinants of child mortality reduction in China between 1990 and 2006: a systematic analysis. J Glob Health.

[25] WHO (2017). Global and regional immunization profile.

[26] Theodoratou E, Johnson S, Jhass A (2010). The effect of *Haemophilus influenzae* type b and pneumococcal conjugate vaccines on childhood pneumonia incidence, severe morbidity and mortality. Int J Epidemiol.

[27] WHO What is quality of care and why is it important?. http://www.who.int/maternal_child_adolescent/topics/quality-of-care/definition/en/.

[28] Edmond K, Scott S, Korczak V (2012). Long term sequelae from childhood pneumonia; systematic review and meta-analysis. PLoS One.

[29] Izadnegahdar R, Fox MP, Jeena P, Qazi SA, Thea DM (2014). Revisiting pneumonia and exposure status in infants born to HIV-infected mothers. Pediatr Infect Dis J.

[30] Kelly MS, Zheng J, Boiditswe S (2017). Investigating mediators of the poor pneumonia outcomes of human immunodeficiency virus-exposed but uninfected children. J Pediatric Infect Dis Soc.

[31] Iroh Tam PY, Wiens MO, Kabakyenga J, Kiwanuka J, Kumbakumba E, Moschovis PP (2018). Pneumonia in HIV-exposed and infected children and association with malnutrition. Pediatr Infect Dis J.

[32] Institute for Health Metrics and Evaluation (2014). Pushing the pace: progress and challenges in fighting childhood pneumonia.

[33] UNICEF (2016). Every breath counts. https://www.unicef.org/health/index_91917.html.

[34] Countdown to 2030 Collaboration (2018). Countdown to 2030: tracking progress towards universal coverage for reproductive, maternal, newborn, and child health. Lancet.

[35] WHO (2018). GAPPD: ending preventable child deaths from pneumonia and diarrhoea by 2025. http://www.who.int/woman_child_accountability/news/gappd_2013/en/.

