# Supplementary material for: Global, regional, and national estimates of pneumonia morbidity and mortality in children younger than 5 years between 2000 and 2015: a systematic analysis
Source: Lancet Glob Health. 2018 Nov 26;7(1):e47–57. doi: 10.1016/S2214-109X(18)30408-X (PMC6293057; doi:10.1016/S2214-109X(18)30408-X)
Supplement: Supplementary appendix [file mmc1.pdf]

THE LANCET
Global Health

Supplementary appendix

This appendix formed part of the original submission and has been peer reviewed. We post it as supplied by the authors.

Supplement to: McAllister DA, Liu L, Shi T, et al. Global, regional, and national estimates of pneumonia morbidity and mortality in children younger than 5 years between 2000 and 2015: a systematic analysis. *Lancet Glob Health* 2018; published online Nov 26. http://dx.doi.org/10.1016/S2214-109X(18)30408-X.

Supplementary material for "Global, regional and national estimates of trends in child pneumonia morbidity and mortality for 2000−2015: a systematic analysis"

CLINICAL PNEUMONIA

SEARCH TERMS (Medline)

#	Searches
1	clinical pneumonia.mp. [mp=title, abstract, original title, name of substance word, subject heading word, keyword heading word, protocol supplementary concept word, rare disease supplementary concept word, unique identifier]
2	severe pneumonia.mp. [mp=title, abstract, original title, name of substance word, subject heading word, keyword heading word, protocol supplementary concept word, rare disease supplementary concept word, unique identifier]
3	exp Respiratory Tract Infections/ep [Epidemiology]
4	pneumonia/ or bronchopneumonia/ or pleuropneumonia/ or pneumonia, bacterial/ or chlamydial pneumonia/ or pneumonia, mycoplasma/ or pneumonia, pneumococcal/ or pneumonia, rickettsial/ or pneumonia, staphylococcal/ or pneumonia, pneumocystis/ or pneumonia, viral/
5	("acute lower respiratory infection" or "alri").mp. [mp=title, abstract, original title, name of substance word, subject heading word, keyword heading word, protocol supplementary concept word, rare disease supplementary concept word, unique identifier]
6	morbidity/ or incidence/ or prevalence/ or mortality/ or epidemiological monitoring/ or geographic mapping/
7	1 or 2 or 3 or 4 or 5
8	6 and 7
9	limit 8 to (yr="1980 -Current" and "all child (0 to 18 years)")

SEARCH TERMS (EMBASE & Global Health)

#	Searches
1	clinical pneumonia.mp. [mp=abstract, title, original title, broad terms, heading words, identifiers, cabicodes]
2	severe pneumonia.mp. [mp=abstract, title, original title, broad terms, heading words, identifiers, cabicodes]
3	[exp Respiratory Tract Infections/ep [Epidemiology]]
4	("acute lower respiratory infection" or "alri").mp. [mp=abstract, title, original title, broad terms, heading words, identifiers, cabicodes]
5	exp pneumonia/
6	1 or 2 or 3 or 4 or 5
7	(incidence or morbidity).mp. [mp=abstract, title, original title, broad terms, heading words, identifiers, cabicodes]
8	6 and 7
**9**	limit 8 to (yr="1980 -Current" and (child <unspecified age> or preschool child <1 to 6 years> or school child <7 to 12 years>))

HOSPITALISED PNEUMONIA

MEDLINE

1. exp Pneumonia/
2. exp Respiratory Tract Infections/ or acute lower respiratory infections.mp.
3. acute respiratory infection$.mp.
4. lower respiratory infection$.mp.
5. exp Bronchiolitis/ or Bronchiolitis, Viral/
6. Pneumococcal Vaccines/ or Haemophilus Vaccines/
7. *Zinc/
8. Vitamin A/
9. exp Incidence/
10. disease burden.mp.
11. exp Morbidity/
12. 1 or 2 or 3 or 4 or 5
13. 6 or 7 or 8
14. 1 and 13
15. 9 or 10 or 11
16. 12 or 14
17. 15 and 16
18. limit 17 to (yr="1 April 2012 -Current" and ("all infant (birth to 23 months)" or "preschool child (2 to 5 years)") and humans)

EMBASE

1. exp respiratory tract infection/ or exp lower respiratory tract infection/ or acute lower respiratory tract infection$.mp. or exp pneumonia/
2. lower respiratory infection$.mp.
3. acute lower respiratory infection$.mp.
4. exp BRONCHIOLITIS/ or VIRAL BRONCHIOLITIS/
5. exp Pneumococcus vaccine/
6. exp Haemophilus influenzae type b vaccine/
7. *ZINC/
8. *alpha tocopherol/ or Vitamin A.mp.
9. exp INCIDENCE/
10. disease burden.mp.
11. exp MORBIDITY/
12. 1 or 2 or 3 or 4
13. 5 or 6 or 7 or 8

14. 1 and 13
15. 9 or 10 or 11
16. 12 or 14
17. 15 and 16
18. limit 17 to (human and yr="1990 -Current" and (infant or preschool child <1 to 6 years>))

GLOBAL HEALTH

1. (pneumonia or lower respiratory tract infections).sh.
2. acute respiratory infection$.mp.
3. exp bronchiolitis/
4. vaccines.sh.
5. (retinol or zinc).sh.
6. exp incidence/
7. disease burden.mp. or morbidity.sh.
8. exp children/
9. 1 or 2 or 3
10. 4 or 5
11. 9 and 10
12. 6 or 7
13. 9 or 11
14. 12 and 13
15. 8 and 14
16. limit 15 to yr="1 April 2012 -Current"

CINAHL

TI Pneumonia

OR

TI Community Acquired Pneumonia

OR

TI Pneumonia Virus$

OR

TI Pneumonia bacteria$

OR

TI bronchiolitis

OR

TI Acute Lower Respiratory Infection.

AND

TI Children

Limiters: 2012-2016; infants: 1 to 23 months & Child, Preschool 2-5 years

Methods for estimating incidence and episodes of clinical pneumonia in young children at country level

Step 1 We conducted a systematic literature review to identify the risk factors for pneumonia in children aged 0-4 years. We selected risk factors which in meta-analyses had strong statistically significant associations with pneumonia. These were:

- Malnutrition (weight for age <-2SD)– odds ratio (OR) 4.5 (2.1-9.5)
- Low birth weight (<2500g) – OR 3.6 (0.8-16.3)
- Non-exclusive breastfeeding (4 months) – OR 2.7 (1.7-4.4)
- Indoor air pollution – OR 1.6 (1.1-2.3)
- HIV – OR 6.51 (5.86-7.24)
- Crowding (more than 5 persons per household) – OR 1.9 (1.5-2.5)
- Incomplete immunisation (No measles immunisation at 12 months of age) – OR 1.8 (1.3-2.5)

Step 2 We obtained data on the prevalence of all risk factors other than HIV from nationally representative household surveys with large sample sizes - the demographic and health surveys (DHS). DHS surveys are generally carried out every 2 to 5 years and provide data on demography and health indicators. The data for China were obtained from the Chinese National Bureau of Statistics. The HIV data were obtained from the United Nations programme on HIV/AIDS (UNAIDS) estimates for 2013 (http://www.unaids.org/en/media/unaids/contentassets/documents/epidemiology/2013/gr2013/20131118_Methodology.pdf). We ensured that the definitions of risk factors were the same in the studies estimating the risk ratios as in the surveys that measured prevalence of exposure to these risk factors.

Specifically, we defined each risk factor using one or more DHS variables. Then, using individual patient data from the standard DHS survey (excluding special surveys) for each country at the survey version closest to 2000 and 2015 respectively, we calculated the proportion of participants with each combination of risk factors.

Step 3 We categorised the developing countries into different subgroups based on the six WHO regions and child and adult mortality strata: A, very low child and very low adult mortality; B, low child and low adult mortality; C, low child and high adult mortality; D, high child and high adult mortality; E, high child and very high adult mortality. Where available, we decided to use the latest data for the prevalence of the seven risk factors. If the prevalence data were not available for any risk factor in a particular country, we imputed the median prevalence for the subgroup to which the country belonged.

Step 4 The overall incidence (of clinical pneumonia in community) and hospitalisation rate (for physician diagnosed pneumonia) in developing countries were obtained from the Poisson regression models as described in the main methods section.

10,000 samples for each prediction were obtained. For the proportion of cases which were severe (i.e. with lower chest wall indrawing), the estimate from random effects meta-analysis (using Stata 11.1) was represented as a beta distribution, from which 10,000 samples were obtained using R. The incidence of severe pneumonia was calculated as the incidence of clinical pneumonia in the community times the proportion with severe pneumonia.

The overall incidence (of clinical and hospitalised pneumonia respectively) in a population is the sum of each stratum specific rate, weighted by the proportion in each stratum where there are *j* mutually exclusive strata:-

$$Total\ rate = \frac{\sum x_j}{\sum n_j} = \sum_j \left(\frac{x_j}{n_j} \cdot \frac{n_0}{x_0} \cdot \frac{x_0}{n_0} \cdot \frac{n_j}{\sum n_j} \right)$$

where x is the number of cases in a stratum, n is the total population in a stratum and where j = 0 indicates the stratum which has no exposures (i.e. the unexposed) and j = 1 to j = 127 indicate the strata for each unique combination of risk factors. This equation can be re-written as:-

$$Total\ rate = \frac{x_0}{n_0} \times \sum j \left(\frac{x_j}{n_j} \cdot \frac{n_0}{x_0} \cdot \frac{n_j}{\sum n_j} \right)$$

where $\frac{x_0}{n_0}$ is the rate in the unexposed, $\frac{x_j}{n_j} \cdot \frac{n_0}{x_0}$ is the rate ratio, and $\frac{n_j}{\sum n_j}$ is the proportion in each stratum.

Therefore, using the regional estimates (for developing countries) of the total rate, the combined rate ratio for each stratum, and the proportion in each stratum we can calculate the incidence in unexposed people for the region (all developing countries). If we then assume that the rate in the unexposed is consistent across the region we can then calculate the incidence rate for each country:-

$$Country\ specific\ rate\ = \frac{\sum x_j^c}{\sum n_j^c} = \frac{\sum x_j^r}{\sum n_j^r} \cdot \frac{\sum_j \left(\frac{x_j}{n_j} \cdot \frac{n_0}{x_0} \cdot \frac{n_j^r}{\sum n_j^r} \right)}{\sum_j \left(\frac{x_j}{n_j} \cdot \frac{n_0}{x_0} \cdot \frac{n_j^c}{\sum n_j^c} \right)}$$

where c indicates a country specific value and r indicates a regional value.

Step 5 Assumptions:

- As with attributable fraction calculations, we assume that the pooled risk ratios obtained from a range of case-control/cohort studies in the developing region were applicable across each developing country. As described in Step 6 we added additional uncertainty to our estimates to relax this assumption somewhat.
- As in our previous analysis for HIV and pneumonia[1] we assumed that there was an n-way interaction between each risk factor (1.25 fold reduction) such that rate ratios combined sub-multiplicatively (e.g. for the group with malnutrition and low birth weight, $RR_{malnutrition}$ = 4.5, and $RR_{low\ birth\ weight}$ = 3.6, RR_{both} = 4.5 x 3.6 / 1.25 = 12.96 rather than 4.5 x 3.6 =16.2). None of the primary studies included in the meta-analysis reported interaction/heterogeneity of effect measure estimates on a multiplicative scale. However, although we noted that the majority of studies did not report testing for interaction (presumably because of an actual or perceived lack of statistical power) it seemed unlikely to us that all risks combined fully multiplicatively. Therefore, we added a weak interaction term to shrink the combined rate ratios towards the null. This would have the effect of causing the country specific estimates to be attenuated towards the overall estimate for the developing region.
- In order to calculate the proportion of the population in each country within each of the j strata we first aggregated the survey data to obtain a marginal (overall) estimate for each risk factor, then assumed independence to estimate the proportion of individuals with each combination of risk factors (i.e. the proportion in each of the j strata). This assumption was made so that the uncertainty around the proportion for each combination of risk factors could be obtained by sampling from 7 beta distributions (with shape parameters determined by the number of participants in the survey with and without each of the 7 risk factors), then multiplying together each of the 7 sampling distributions. We could instead have used the actual number of participants with each combination of risk factors, and sampled instead from a Dirichlet distribution (a multivariate extension of the beta distribution). However, our chosen approach had a number of advantages. Firstly, it allowed us to use data from more than one source (as maternal HIV is not well recorded in the DHS surveys). Secondly, data processing was more straightforward. Thirdly, it meant that we could make the data used in the analyses publicly available, which would not have been possible had we used the actual joint proportions, which are potentially disclosive. We examined the extent to which our assumption of independence over or underestimated each joint proportion across 102 DHS surveys (See Table below). For the purpose of this tabulation alone, self-reported maternal sexually transmitted infection was used as a surrogate for HIV infection.

- In 97% of the 127 strata (in 102 DHS surveys) where people had one or more of the seven risk factors, the estimated proportion was within 5% of the true proportion. The independence assumption meant that we tended to overestimate the true proportion of participants with no risk factors or few risk factors, as expected given that these risk factors are in fact correlated. This means that the model will tend to underestimate differences between countries on average, although it may overestimate or underestimate the incidence rate depending on the actual correlation of risk factors in particular countries.
- Finally we assumed that the rate among the unexposed (i.e. the risk in the absence of these seven risk factors) came from the same distribution for all countries. This is the same as assuming that there was no residual confounding.

Step 6 Using a simulation based approach, we performed the calculation described in Step 4 to estimate the country specific rates along with uncertainty estimates as uncertainty ranges (UR).

This was done by performing this calculation for each of the 10,000 samples from the distributions representing the incidence rates, disease severity proportion, odds ratios and risk factor proportions (methods used to obtain each of these were obtained as described above) within R. Full analysis code and data are available from the authors on request.

Step 7 In a similar manner to equation 3 within step 4, we calculated the incidence of pneumonia in HIV-uninfected children by summing those strata where children were HIV negative regardless of the presence/absence of other risk factors. Similarly, we calculated the incidence of pneumonia in HIV-infected children.

Step 8 Having obtained estimates of the total rate and rate in HIV-uninfected children in each country, we used the standard formula for attributable fraction.[1]

$$Attributable\ fraction = \frac{I_e - I_0}{I_e}$$

$$or, equivalently, attributable\ fraction = \frac{RR - 1}{RR}$$

where I_e is the incidence in HIV-infected and I_0 is the incidence in HIV-uninfected in each country; and RR is the risk ratio for pneumonia in HIV-infected (compared to -uninfected). We then applied the attributable fraction to the rate among the HIV-infected (and the UR) in order to obtain the rate attributable to HIV with the UR. As we had already incorporated uncertainty in the HIV risk factor estimates in calculating the total country rates, we did not add additional uncertainty at this stage.

Supplementary table 1: Impact of obtaining joint proportions from marginal proportions, using assumption of independence

Risk factors	Independence assumption leads to overestimation (> 5%)	Independence assumption correct within 5% of true value	Independence assumption leads to underestimation (> 5%)
0	77(75.5)	14(13.7)	11(10.8)
1	238(33.3)	466(65.3)	10(1.4)
2	258(12.0)	1879(87.7)	5(0.2)
3	97(2.7)	3469(97.2)	4(0.1)
4	1(0.0)	3566(99.9)	3(0.1)
5	0(0.0)	2142(100.0)	0(0.0)
6	0(0.0)	714(100.0)	0(0.0)
7	0(0.0)	102(100.0)	0(0.0)

Cells contain the number and (%) of strata in which proportions are overestimated, underestimated and correctly estimated, for 102 DHS surveys.

Supplementary table 2: Details of studies reporting incidence of clinical pneumonia in children younger than 5 years

S.No	Location (study period)	Region	Duration of study (months)	Age group under study (months)	Case definition		Status of Hib vaccine implementation at time of study	Status of PCV implementation at time of study (indicate valency of the vaccine)	Assessor	Surveillance intervals	Site of diagnosis	Denominator (cohort size)	Incidence 0-4 years (episodes/child-year)	Proportion of cases with severe pneumonia
					Clinical pneumonia	Severe pneumonia								
**1**	Ibadan, Nigeria; urban (Oct 1984 - Oct 1987)[2]	AFR	36	0-59	IC, W-, CR+		No	No	FW (and Physician)	Weekly	Clinic and home	610	1.38	
**2**	Maragua, Kenya; rural (Feb 1985 - Jan 1988)[3]	Africa	36	0-59	IC, W-, CR+		No	No	FW (and Physician)	Weekly	Clinic and home	470	0.16	
**3**	Basse, The Gambia; rural (Mar 1987 - Mar 1988)[4]	Africa	12	0-59	IB, W-, CR-		No	No	TFW (and Physician)	Weekly	Home and hospital	491	0.45	
**4**	Accra, Ghana; rural (Jan 1987 - Dec 1989)[5]	Africa	36	0-59	IB, W-, CR-		No	No	Nurses (and Physician)	Weekly	Clinic and home	1350	0.06	
**5**	Ilorin, Nigeria; suburban (Jul 1988 - Jun 1989)[6]	Africa	12	0-59	IA, W+, CR-	IA, W+, CR+	No	No	TFW	3x/week	Home	481	1.65	0.11
**6**	Ibadan, Nigeria; urban and rural (Jun 1999 - May 2001)[7]	Africa	24	0-59	IB, W-, CR-		No	No	Nurses (TFW)	Weekly	Home and hospital	1579	0.27	

S.No	Location (study period)	Region	Duration of study (months)	Age group under study (months)	Case definition		Status of Hib vaccine implementation at time of study	Status of PCV implementation at time of study (indicate valency of the vaccine)	Assessor	Surveillance interval s	Site of diagnosis	Denominator (cohort size)	Incidence 0-4 years (episodes/child-year)	Proportion of cases with severe pneumonia
					Clinical pneumonia	Severe pneumonia								
**7**	Upper and Central River Division, The Gambia; rural (Aug 2000 - Apr 2004))[8]	Africa	45	3-29	IB, W-, CR-	II	No	No	Nurses (and Physician)	Weekly	Hospital	8151	0.2	0.07
**8**	Paarl, South Arica; periurban (May 2012 - May 2014)[9]	Africa	24	0-11	IA, W-, CR-	IA, W-, CR+	Yes	Yes	TFW (and Physician)	Not known	Clinic and hospital	697	0.22	0.23
**9**	Monte-video, Uruguay; urban (May 1985 - Dec 1987)[10]	Americas	32	0-35	II		No	No	Pediatrician	10 days	Home	166	2.26	
**10**	Guatemala city, Guatemala; suburban (Jan 1985 - Dec 1986)[11]	Americas	24	0-59	IC, W-, CR+		No	No	TFW (and Physician)	Fortnightly	Clinic	521	0.31	
**11**	Cali, Colombia; urban (Jan 1987 - Dec 1989)[12]	Americas	36	0-17	IC, W-, CR+		No	No	TFW	Weekly	Clinic	340	1.26	
**12**	San Marcos, Guatemala; rural (Oct 2002 - Dec 2004)[13]	Americas	15	0-59	II	II	No	No	Physician	Weekly	Home	253	0.28	0.34

S.No	Location (study period)	Region	Duration of study (months)	Age group under study (months)	Case definition		Status of Hib vaccine implementation at time of study	Status of PCV implementation at time of study (indicate valency of the vaccine)	Assessor	Surveillance intervals	Site of diagnosis	Denominator (cohort size)	Incidence 0-4 years (episodes/child-year)	Proportion of cases with severe pneumonia
					Clinical pneumonia	Severe pneumonia								
**13**	Canto Grande, Peru; suburban (Jul 1987 - Oct 1989)[14]	Americas	27	0-41	II		No	No	Physician (and TFW)	2x/week	Home and clinic	1500	0.26	
**14**	Ghizer, Pakistan; rural (Nov 2001 - Dec 2002)[15]	Eastern Mediterranean	14	2-35	IC, W-, CR-	IC, W-, CR+	No	No	TFW	Fortnightly	Home and hospital	5204	0.23	0.27
**15**	Karachi, Pakistan; urban (Apr 2002 - Apr 2003)[16]	Eastern Mediterranean	12	0-59	IA, W-, CR-		No	No	TFW	Weekly	Home	1634	0.9	
**16**	Haryana, India; rural (Jan 1982 - Sep 1983)[17]	South East Asia	21	0-23	IB, W+, CR-		No	No	TFW	Weekly	Home	347	0.24	
**17**	Kathmandu, Nepal; rural (Feb 1984 - Jan 1987)[18]	South East Asia	36	0-59	IB, W+, CR+		No	No	TFW	Fortnightly	Home	1019	0.22	
**18**	Bangkok, Thailand; urban (Jan 1986 - Dec 1987)[19]	South East Asia	24	0-59	IC, W-, CR+		No	No	TFW (and Physician)	2x/week	Home	674	0.07	
**19**	Haryana, India; rural (Jan 1986 - Dec 1986)[20]	South East Asia	12	0-59	IB, W-, CR+		No	No	TFW	Fortnightly	Home	5078	0.54	

S.No	Location (study period)	Region	Duration of study (months)	Age group under study (months)	Case definition		Status of Hib vaccine implementation at time of study	Status of PCV implementation at time of study (indicate valency of the vaccine)	Assessor	Surveillance intervals	Site of diagnosis	Denominator (cohort size)	Incidence 0-4 years (episodes/child-year)	Proportion of cases with severe pneumonia
					Clinical pneumonia	Severe pneumonia								
**20**	Yumla, Nepal; rural (Jun 1986 - Jun 1989)[21]	South East Asia	36	0-59	IA, W+, CR-		No	No	TFW	Fortnightly	Home	13404	0.7	
**21**	Matlab, Bangladesh; rural (May 1988 - Apr 1989)[22]	South East Asia	12	0-59	IB, W-, CR-		No	No	TFW	2x/week	Home	696	0.23	
**22**	Wardha, India; rural (Sep 1990 - Aug 1991)[23]	South East Asia	12	0-59	IA, W+, CR-		No	No	Not reported (probably TFW)	Fortnightly	Home	384	0.08	
**23**	Mirzapur, Bangladesh; rural (Oct 1993 - Sep 1996)[24]	South East Asia	36	0-23	IB, W+, CR-	IB, W+, CR+	No	No	TFW (and pediatrician)	2x/week	Home and hospital	288	0.38	0.26
**24**	Bandung, Indonesia; suburban and rural (Feb 1999 - Jan 2001)[7]	South East Asia	24	0-59	IC, W+, CR-	IC, W+, CR+	No	No	TFW	Weekly	Home and clinic	1420	0.19	0.12

S.No	Location (study period)	Region	Duration of study (months)	Age group under study (months)	Case definition		Status of Hib vaccine implementation at time of study	Status of PCV implementation at time of study (indicate valency of the vaccine)	Assessor	Surveillance intervals	Site of diagnosis	Denominator (cohort size)	Incidence 0-4 years (episodes/child-year)	Proportion of cases with severe pneumonia
					Clinical pneumonia	Severe pneumonia								
**25**	Ballabgarh, Haryana, India; rural (Oct 2001 - Mar 2005)[25]	South East Asia	42	0-35	IC, W-, CR-	IC, W-, CR+	No	No	TFW (and Physician)	Weekly	Home and hospital	281	0.24	0.17
**26**	Dhaka, Bangladesh; urban (Apr 2004 - Dec 2007)[26]	South East Asia	45	0-59	IC, W-, CR-	IC, W+, CR+	No	No	TFW (and Physician)	Weekly	Home and hospital	12062	0.47	0.070
**27**	Mirzapur, Bangladesh; rural (Jul 2004 - Jun 2007)[27]	South East Asia	36	0-59	IC, W+, CR-	IC, W+, CR+	No	No	TFW (and Physician)	Weekly	Home and hospital	22378	0.31	0.53
**28**	Delhi, India; periurban (Jan 2011 - Jan 2012)[28]	South East Asia	12	0-59	IA, W-, CR-	IA, W-, CR+	No	No	TFW (and Physician)	Fortnightly	Home and hospital	106	0.37	0.06
**29**	Tari & Asaro, Papua New Guinea; rural (Jan 1979 - Jan 1983)[29]	Western Pacific	48	0-59	IB, W-, CR-	IB, W-, CR+	No	No	TFW (and Physician)	Fortnightly	Home and hospital	1595	0.41	0.7
**30**	Changping county, China; semirural (Jun 1981 - Jun 1983)[30]	Western Pacific	24	0-143	II		No	No	Physician	Weekly	Home and primary school	526	0.07	

S.No	Location (study period)	Region	Duration of study (months)	Age group under study (months)	Case definition		Status of Hib vaccine implementation at time of study	Status of PCV implementation at time of study (indicate valency of the vaccine)	Assessor	Surveillance intervals	Site of diagnosis	Denominator (cohort size)	Incidence 0-4 years (episodes/child-year)	Proportion of cases with severe pneumonia
					Clinical pneumonia	Severe pneumonia								
**31**	Albang Manilla, Phillipines; urban (Apr 1985 - Mar 1987)[31]	Western Pacific	24	0-59	IC, W-, CR+		No	No	Nurse (and Physician)	Weekly	Home and clinic	1978	0.53	
**32**	Asaro valley, Papua New Guinea; rural (Jan 1985 - Dec 1987)[32]	Western Pacific	36	0-59	IB, W+, CR-		No	No	Lay reporters	2x/week	Home	156	1.32	
**33**	Zhejiang Province, China; rural (Apr 1990 - Mar 1991)[33]	Western Pacific	12	0-59	IA, W-, CR-		No	No	TFW (and Physician)	Fortnightly	Home and clinic	1215	0.10	
**34**	Zhejiang Province, China; rural (Apr 1990 - Mar 1991)[34]	Western Pacific	12	0-59	IA, W-, CR-		No	No	TFW (and Physician)	Monthly	Home and clinic	7472	0.13	
**35**	Rudong County, Jiangsu Province, China; rural (Feb 1991 - Jan 1993)[35]	Western Pacific	12	0-59	IA, W-, CR-		No	No	TFW (and Physician)	Not known	Home, clinic and hospital	10541	0.28	
**36**	Liyang City, China; rural (1991 - 1994)[36]	Western Pacific	36	0-59	IA, W-, CR-		No	No	TFW (and Physician)	Not known	Clinic and hospital	20867	0.12	

S.No	Location (study period)	Region	Duration of study (months)	Age group under study (months)	Case definition		Status of Hib vaccine implementation at time of study	Status of PCV implementation at time of study (indicate valency of the vaccine)	Assessor	Surveillance intervals	Site of diagnosis	Denominator (cohort size)	Incidence 0-4 years (episodes/child-year)	Proportion of cases with severe pneumonia
					Clinical pneumonia	Severe pneumonia								
**37**	Changshou County, China; rural (Feb 1992 - Jan 1993)[37]	Western Pacific	12	0-59	IA, W-, CR-		No	No	TFW	Not known	Clinic	2246	0.19	
**38**	Huanan and Keshan County, Heilongjiang Province; rural (Jan 1993 - Dec 1993)[38]	Western Pacific	12	0-59	IA, W-, CR-		No	No	TFW	Not known	Clinic	5812	0.16	
**39**	Guangzhou, China; urban (Oct 1993 - Sep 1998)[39]	Western Pacific	60	0-59	IC, III		No	No	TFW	Not known	Clinic	120970	0.32	
**40**	Liyang City, China; rural (Feb 1994 - Jan 1995)[40]	Western Pacific	12	0-59	IA, W-, CR-		No	No	TFW (and Physician)	Not known	Clinic and hospital	11729	0.12	
**41**	Henan Province, China; rural and urban (Jan 1994 - Dec 1994)[41]	Western Pacific	12	0-59	IA, W-, CR-		No	No	TFW (and Physician)	Not known	Clinic and hospital	7917	0.11	
**42**	Licheng District, Quanzhou City, China; rural (Oct 1994 - Sept 1995)[42]	Western Pacific	12	0-59	IA, W-, CR-		No	No	TFW (and Physician)	Not known	Clinic and hospital	4665	0.13	

S.No	Location (study period)	Region	Duration of study (months)	Age group under study (months)	Case definition		Status of Hib vaccine implementation at time of study	Status of PCV implementation at time of study (indicate valency of the vaccine)	Assessor	Surveillance intervals	Site of diagnosis	Denominator (cohort size)	Incidence 0-4 years (episodes/child-year)	Proportion of cases with severe pneumonia
					Clinical pneumonia	Severe pneumonia								
**43**	Huaning County, Yunnan Province, China (Jan 1995 - Dec 1997)[43]	Western Pacific	24	0-59	IA, W-, CR-		No	No	TFW (and Physician)	Not known	Clinic and hospital	6966	0.66	
**44**	Hubei Province, China; rural (Jul 1996- Jun 1999)[44]	Western Pacific	36	0-59	IA, W-, CR-		No	No	TFW	Not known	Clinic	75337	0.64	
**45**	Jinan City, China; urban (Jan 1995- Dec 2001)[45]	Western Pacific	84	0-59	II		No	No	TFW	Not known	Clinic	321249	0.22	
**46**	Haryana, India; rural (Aug 2012- Aug 2013)[46]	South East Asia	12	0-119	IB, W-, CR-		No	No	TFW	Weekly	Home and hospital	1782	0.37	

Supplementary table 3: Incidence of clinical pneumonia in young children in developing countries (2000-2015)

Year	Incidence rate per 1000 children per year	Number of episodes of clinical pneumonia (millions)
**2000**	329 (201-537)	178 (110-289)
**2005**	292 (179-477)	162 (100-263)
**2010**	259 (159-424)	150 (92-244)
**2015**	231 (141-377)	138 (86-226)

Supplementary table 4: Incidence and episodes of clinical pneumonia in children younger than 5 years in 2000

Country	Incidence of clinical pneumonia (per 1000 children per year) in children younger than 5 years	Episodes of clinical pneumonia in children younger than 5 years	Incidence of clinical pneumonia in HIV infected children (per 1000 children per year)	Episode of clinical pneumonia in HIV infected children	Episodes of clinical pneumonia attributable to HIV
**Afghanistan**	598 (325-1122)	2551075 (1383857-4783262)	3106 (1662-5850)	13240 (7084-24940)	10703 (5687-20272)
**Algeria**	282 (170-472)	877260 (528817-1467580)	1467 (876-2493)	4590 (2732-8033)	3706 (2191-6511)
**Angola**	327 (198-538)	892424 (540837-1468768)	1684 (1011-2810)	14149 (5974-30437)	11427 (4808-24620)
**Antigua and Barbuda**	191 (106-338)	1479 (824-2621)	1000 (551-1796)	8 (4-14)	6 (3-12)
**Argentina**	191 (106-337)	657436 (365513-1162540)	999 (551-1789)	3481 (1914-6511)	2819 (1542-5293)
**Bahamas**	191 (106-339)	5377 (2986-9541)	998 (551-1795)	43 (19-108)	35 (16-88)
**Bahrain**	302 (160-590)	21901 (11599-42773)	1579 (824-3114)	115 (60-232)	93 (48-188)
**Bangladesh**	361 (218-601)	6041515 (3655788-10059442)	1875 (1116-3153)	31396 (18674-52847)	25369 (14992-42892)
**Barbados**	191 (106-338)	3523 (1955-6236)	999 (551-1795)	19 (10-35)	15 (8-29)
**Belize**	266 (153-459)	9138 (5273-15782)	1375 (786-2403)	103 (46-217)	83 (37-176)
**Benin**	520 (301-892)	647464 (374548-1111086)	2675 (1530-4633)	10717 (4464-23843)	8660 (3597-19332)
**Bhutan**	216 (121-387)	16262 (9112-29089)	1134 (633-2045)	85 (48-154)	69 (38-126)
**Bolivia**	277 (154-495)	337941 (187863-603328)	1444 (794-2591)	1845 (999-3751)	1491 (803-3051)
**Botswana**	437 (263-729)	99258 (59597-165451)	1936 (1150-3252)	18501 (10799-31635)	14960 (8709-25745)
**Brazil**	244 (130-448)	4256325 (2271522-7820093)	1269 (670-2367)	22998 (11948-47240)	18586 (9602-38436)
**Burkina Faso**	349 (205-590)	757451 (446236-1280901)	1762 (1030-3001)	28310 (14915-52310)	22857 (12029-42352)
**Burundi**	380 (228-632)	455332 (273225-756144)	1937 (1146-3256)	12826 (6746-24414)	10367 (5414-19818)
**Cambodia**	462 (248-801)	686893 (368951-1191233)	2386 (1278-4189)	6749 (2988-14503)	5451 (2405-11793)
**Cameroon**	241 (144-407)	665451 (397146-1121198)	1197 (706-2041)	42650 (20601-85216)	34477 (16573-69189)
**Cape Verde**	325 (197-537)	20141 (12234-33279)	1684 (1013-2823)	138 (72-358)	112 (58-291)
**Central African Republic**	534 (306-923)	315982 (180965-546095)	2589 (1467-4510)	26995 (14341-50351)	21806 (11505-40696)

Country	Incidence of clinical pneumonia (per 1000 children per year) in children younger than 5 years	Episodes of clinical pneumonia in children younger than 5 years	Incidence of clinical pneumonia in HIV infected children (per 1000 children per year)	Episode of clinical pneumonia in HIV infected children	Episodes of clinical pneumonia attributable to HIV
**Chad**	528 (279-947)	889337 (469358-1594283)	2673 (1401-4856)	30044 (13177-63028)	24260 (10571-51284)
**Chile**	191 (106-338)	253993 (141123-451107)	999 (552-1792)	1341 (737-2488)	1086 (594-2017)
**China**	259 (146-460)	21664371 (12245213-38478707)	1355 (753-2429)	123010 (66254-368509)	99749 (53155-299137)
**Colombia**	199 (109-359)	886715 (487441-1599359)	1042 (565-1905)	5032 (2644-11565)	4076 (2129-9387)
**Comoros**	466 (267-800)	39824 (22834-68374)	2418 (1373-4228)	228 (124-754)	184 (100-611)
**Congo**	210 (116-382)	108072 (59727-196704)	1049 (572-1929)	7323 (3758-14298)	5935 (3031-11624)
**Cook Islands**	259 (146-461)	565 (318-1006)	1355 (755-2434)	4 (2-12)	3 (1-10)
**Costa Rica**	190 (106-338)	75133 (41738-133190)	999 (550-1793)	398 (218-740)	322 (176-603)
**Cote d'Ivoire**	315 (184-546)	831445 (484438-1440515)	1527 (882-2672)	73607 (38051-139556)	59479 (30609-113495)
**Dem. Peoples's Rep. of Korea**	341 (203-575)	676397 (401937-1139705)	1770 (1042-3014)	3511 (2066-5977)	2837 (1664-4851)
**Dem. Rep. of the Congo**	413 (248-685)	3650795 (2197093-6059257)	2122 (1265-3565)	60481 (30828-115511)	48864 (24850-93872)
**Djibouti**	193 (105-351)	19012 (10360-34615)	995 (540-1824)	375 (180-751)	303 (145-608)
**Dominica**	190 (106-338)	1324 (738-2351)	999 (552-1792)	7 (4-13)	6 (3-11)
**Dominican Republic**	187 (103-334)	191800 (105433-343156)	969 (526-1746)	3106 (751-12577)	2519 (607-10197)
**Ecuador**	191 (106-338)	286484 (158824-508053)	999 (551-1795)	1537 (839-3056)	1245 (675-2485)
**Egypt**	151 (78-287)	1177828 (607352-2238003)	798 (408-1526)	6229 (3185-11916)	5057 (2575-9728)
**El Salvador**	265 (153-456)	205855 (118928-354492)	1376 (788-2393)	1128 (624-2290)	912 (503-1855)
**Equatorial Guinea**	287 (172-481)	24450 (14649-40939)	1468 (873-2493)	624 (313-1208)	504 (252-981)
**Eritrea**	376 (225-626)	252602 (151360-420420)	1935 (1148-3256)	3819 (1767-7705)	3083 (1425-6238)
**Ethiopia**	349 (192-602)	4315381 (2372433-7441136)	1734 (946-2999)	249513 (132475-444146)	201532 (106512-360140)

Country	Incidence of clinical pneumonia (per 1000 children per year) in children younger than 5 years	Episodes of clinical pneumonia in children younger than 5 years	Incidence of clinical pneumonia in HIV infected children (per 1000 children per year)	Episode of clinical pneumonia in HIV infected children	Episodes of clinical pneumonia attributable to HIV
**Fiji**	259 (146-461)	25507 (14382-45446)	1356 (754-2431)	146 (78-436)	118 (63-354)
**Gabon**	323 (184-570)	59423 (33903-104862)	1629 (917-2895)	2394 (1206-4723)	1933 (973-3834)
**Gambia**	374 (224-622)	86442 (51801-143743)	1937 (1149-3259)	702 (333-1595)	567 (268-1291)
**Ghana**	286 (161-483)	816597 (459248-1380859)	1454 (814-2498)	25458 (11905-51720)	20554 (9586-41929)
**Grenada**	191 (106-338)	1993 (1112-3532)	999 (551-1786)	11 (6-19)	9 (5-16)
**Guatemala**	388 (233-649)	721079 (432287-1207098)	2014 (1188-3409)	3817 (2246-7170)	3082 (1805-5817)
**Guinea**	313 (187-524)	472902 (282938-792242)	1611 (953-2724)	6397 (2921-13360)	5160 (2344-10844)
**Guinea-Bissau**	384 (230-643)	82753 (49494-138394)	1937 (1147-3256)	3368 (1833-6129)	2723 (1475-4983)
**Guyana**	265 (153-459)	23147 (13409-40094)	1376 (787-2411)	144 (76-330)	116 (61-267)
**Haiti**	317 (169-558)	387837 (207366-682802)	1597 (848-2842)	15817 (7937-29997)	12780 (6378-24403)
**Honduras**	201 (118-347)	189792 (110776-327403)	1044 (603-1820)	2177 (1063-4383)	1760 (858-3569)
**India**	366 (219-613)	45046369 (26965775-75354251)	1902 (1125-3197)	233949 (138393-393122)	189006 (110999-319705)
**Indonesia**	487 (288-815)	10234904 (6048250-17145028)	2526 (1465-4288)	53117 (30817-90179)	42901 (24758-73130)
**Iran (Islamic Republic of)**	302 (160-593)	1909614 (1010586-3748381)	1581 (827-3124)	10062 (5241-20226)	8134 (4227-16400)
**Iraq**	191 (104-347)	736872 (402841-1341896)	996 (538-1821)	3850 (2081-7037)	3111 (1673-5724)
**Jamaica**	191 (107-340)	54224 (30190-96254)	999 (551-1798)	557 (227-1333)	452 (183-1083)
**Jordan**	283 (136-586)	196313 (94252-406342)	1470 (703-3079)	1021 (489-2141)	825 (394-1739)
**Kenya**	402 (230-694)	2052821 (1174951-3545949)	1892 (1076-3320)	243421 (133266-441954)	196827 (107284-359187)
**Kiribati**	259 (146-460)	2568 (1447-4567)	1355 (757-2425)	15 (8-46)	12 (6-37)
**Kuwait**	302 (159-592)	56363 (29726-110517)	1579 (825-3113)	296 (155-598)	240 (125-486)

Country	Incidence of clinical pneumonia (per 1000 children per year) in children younger than 5 years	Episodes of clinical pneumonia in children younger than 5 years	Incidence of clinical pneumonia in HIV infected children (per 1000 children per year)	Episode of clinical pneumonia in HIV infected children	Episodes of clinical pneumonia attributable to HIV
**Lao People's Dem. Republic**	460 (248-799)	374893 (202336-651223)	2386 (1276-4188)	1945 (1040-3413)	1571 (838-2771)
**Lebanon**	302 (160-592)	92236 (48850-180689)	1580 (828-3108)	485 (253-979)	393 (204-794)
**Lesotho**	380 (227-641)	102662 (61166-172908)	1627 (958-2773)	23007 (13242-40055)	18598 (10655-32523)
**Liberia**	240 (128-434)	119385 (63357-215525)	1231 (647-2234)	3517 (1696-7050)	2847 (1366-5725)
**Libyan Arab Jamahiriya**	302 (160-593)	165612 (87544-325316)	1579 (827-3115)	871 (455-1731)	705 (367-1402)
**Madagascar**	456 (274-755)	1296879 (777902-2144110)	2357 (1402-3949)	9540 (4726-24034)	7705 (3797-19558)
**Malawi**	396 (235-669)	811482 (481185-1370637)	1758 (1028-3006)	149884 (86737-257893)	121128 (69795-209479)
**Malaysia**	460 (247-801)	1252567 (672604-2180834)	2385 (1276-4201)	6490 (3471-11430)	5245 (2794-9263)
**Maldives**	207 (116-372)	7140 (4021-12830)	1095 (607-1977)	38 (21-68)	31 (17-56)
**Mali**	362 (214-607)	694702 (411142-1165704)	1855 (1089-3136)	13650 (6707-26332)	11023 (5403-21371)
**Marshall Islands**	259 (146-461)	1620 (912-2884)	1356 (755-2427)	9 (5-30)	8 (4-25)
**Mauritania**	325 (197-534)	143839 (86991-236454)	1685 (1010-2820)	906 (493-2321)	733 (397-1887)
**Mauritius**	283 (169-471)	27851 (16707-46445)	1468 (873-2485)	146 (87-263)	118 (70-214)
**Mexico**	191 (106-338)	2343698 (1305052-4157848)	999 (553-1792)	12343 (6805-22718)	9997 (5480-18403)
**Micronesia (Fed. States of)**	259 (146-460)	3879 (2194-6895)	1355 (755-2427)	22 (12-69)	18 (10-56)
**Mongolia**	259 (146-458)	60535 (34233-107205)	1355 (753-2426)	343 (183-1015)	278 (147-826)
**Morocco**	263 (148-464)	821577 (461136-1447938)	1368 (760-2438)	4272 (2374-7628)	3453 (1907-6206)
**Mozambique**	405 (243-677)	1287681 (771694-2151012)	2003 (1189-3368)	82950 (46307-147298)	67018 (37264-119272)
**Myanmar**	353 (195-635)	1715988 (945637-3081256)	1836 (1001-3325)	9168 (4930-17672)	7407 (3972-14306)
**Namibia**	372 (224-622)	102839 (61903-171772)	1712 (1020-2890)	15194 (8706-26572)	12283 (6991-21557)
**Nauru**	259 (146-461)	312 (176-555)	1355 (757-2429)	2 (1-8)	2 (1-7)

Country	Incidence of clinical pneumonia (per 1000 children per year) in children younger than 5 years	Episodes of clinical pneumonia in children younger than 5 years	Incidence of clinical pneumonia in HIV infected children (per 1000 children per year)	Episode of clinical pneumonia in HIV infected children	Episodes of clinical pneumonia attributable to HIV
**Nepal**	336 (195-579)	1164550 (677745-2008253)	1747 (1011-3042)	6069 (3511-10624)	4909 (2813-8646)
**Nicaragua**	236 (135-411)	166240 (94929-289477)	1227 (694-2163)	866 (489-1538)	701 (393-1246)
**Niger**	519 (295-889)	1173726 (666126-2009603)	2686 (1506-4642)	10130 (4465-23403)	8178 (3600-18953)
**Nigeria**	401 (220-694)	8459265 (4643080-14631547)	2039 (1111-3569)	254032 (124773-489847)	204837 (100444-397171)
**Niue**	467 (251-812)	109 (58-189)	2386 (1277-4182)	2 (1-5)	2 (1-4)
**Oman**	302 (160-594)	81486 (43251-160364)	1580 (828-3126)	431 (224-876)	349 (181-711)
**Pakistan**	195 (104-358)	4099287 (2188254-7533266)	1012 (535-1870)	21309 (11257-39354)	17200 (9075-32022)
**Palau**	460 (248-799)	1058 (569-1838)	2384 (1279-4184)	6 (3-10)	4 (2-8)
**Panama**	191 (106-338)	66520 (37104-117916)	999 (552-1794)	366 (197-785)	296 (159-638)
**Papua New Guinea**	259 (146-461)	217962 (122918-387468)	1356 (757-2426)	1830 (758-6413)	1483 (611-5190)
**Paraguay**	265 (153-456)	188106 (108930-324473)	1375 (789-2402)	978 (562-1709)	791 (451-1391)
**Peru**	197 (112-348)	593405 (336178-1044596)	1030 (580-1838)	3123 (1758-5624)	2527 (1414-4568)
**Philippines**	521 (306-885)	5569601 (3263920-9457115)	2705 (1564-4640)	28893 (16715-49616)	23351 (13433-40141)
**Qatar**	302 (160-595)	16773 (8877-33019)	1580 (826-3121)	88 (46-177)	71 (37-143)
**Republic of Korea**	259 (146-460)	809678 (455843-1440605)	1355 (758-2428)	4591 (2456-13273)	3725 (1973-10831)
**Rwanda**	237 (137-404)	397823 (228977-677865)	1189 (680-2049)	19478 (10612-35151)	15725 (8541-28588)
**Saint Kitts and Nevis**	191 (106-338)	867 (481-1537)	999 (553-1789)	5 (3-9)	4 (2-7)
**Saint Lucia**	191 (106-339)	3020 (1686-5363)	999 (553-1797)	16 (9-29)	13 (7-24)
**Saint Vincent and Grenadines**	191 (106-338)	2085 (1158-3696)	999 (552-1791)	11 (6-20)	9 (5-16)
**Samoa**	258 (145-460)	6615 (3724-11778)	1353 (758-2431)	38 (20-114)	31 (16-93)
**Sao Tome and Principe**	331 (200-544)	7162 (4339-11775)	1685 (1014-2812)	204 (101-395)	165 (82-320)

Country	Incidence of clinical pneumonia (per 1000 children per year) in children younger than 5 years	Episodes of clinical pneumonia in children younger than 5 years	Incidence of clinical pneumonia in HIV infected children (per 1000 children per year)	Episode of clinical pneumonia in HIV infected children	Episodes of clinical pneumonia attributable to HIV
**Saudi Arabia**	302 (160-592)	846478 (447812-1658882)	1578 (827-3109)	4451 (2327-8919)	3598 (1874-7245)
**Senegal**	278 (167-469)	467974 (282049-790009)	1440 (857-2448)	3053 (1628-6775)	2466 (1311-5493)
**Seychelles**	282 (170-471)	2054 (1235-3426)	1468 (874-2481)	11 (6-19)	9 (5-15)
**Sierra Leone**	385 (228-649)	270598 (160436-456733)	1996 (1173-3396)	1821 (943-4337)	1472 (756-3513)
**Solomon Islands**	259 (146-460)	16733 (9415-29777)	1355 (753-2426)	95 (51-278)	77 (41-227)
**Somalia**	576 (313-1037)	845515 (460326-1522995)	2980 (1611-5414)	5839 (2831-12872)	4715 (2278-10478)
**South Africa**	473 (275-817)	2287592 (1327850-3950583)	2212 (1263-3861)	293956 (163330-521477)	237464 (131595-423254)
**Sri Lanka**	237 (133-431)	393835 (221526-715052)	1237 (689-2256)	2054 (1144-3746)	1662 (920-3035)
**Sudan**	191 (104-347)	902948 (491641-1643377)	995 (539-1821)	4711 (2549-8636)	3810 (2054-7030)
**Suriname**	191 (106-338)	9634 (5353-17033)	1000 (549-1792)	88 (37-214)	71 (30-173)
**Swaziland**	441 (263-735)	69222 (41325-115306)	1937 (1149-3249)	13442 (7829-22820)	10856 (6301-18547)
**Syrian Arab Republic**	191 (104-346)	457405 (249915-830569)	995 (537-1823)	2387 (1289-4372)	1929 (1039-3557)
**Thailand**	207 (104-406)	967387 (487365-1892866)	1079 (538-2132)	5706 (2746-11858)	4612 (2216-9640)
**Timor-Leste**	328 (179-580)	57165 (31217-101160)	1701 (918-3038)	297 (160-530)	239 (129-430)
**Togo**	513 (287-894)	414104 (231712-721224)	2554 (1420-4517)	22939 (11807-43200)	18520 (9520-35137)
**Tonga**	259 (146-459)	3443 (1941-6108)	1355 (753-2423)	20 (11-61)	16 (8-50)
**Trinidad and Tobago**	191 (107-338)	17113 (9553-30309)	999 (550-1790)	124 (57-320)	101 (46-260)
**Tunisia**	271 (140-555)	225313 (116572-461328)	1423 (730-2916)	1296 (634-3868)	1049 (511-3135)
**Tuvalu**	259 (146-462)	299 (168-533)	1354 (756-2436)	2 (1-8)	2 (1-6)
**Uganda**	287 (170-482)	1384676 (822972-2328519)	1388 (821-2360)	119754 (69045-210786)	96713 (55411-170809)
**United Arab Emirates**	302 (160-593)	78282 (41430-153692)	1581 (824-3115)	413 (214-822)	334 (174-668)

Country	Incidence of clinical pneumonia (per 1000 children per year) in children younger than 5 years	Episodes of clinical pneumonia in children younger than 5 years	Incidence of clinical pneumonia in HIV infected children (per 1000 children per year)	Episode of clinical pneumonia in HIV infected children	Episodes of clinical pneumonia attributable to HIV
**United Republic of Tanzania**	281 (170-465)	1655983 (1003583-2747126)	1328 (798-2235)	194405 (110677-342126)	157023 (89146-277747)
**Uruguay**	190 (106-337)	51639 (28699-91377)	1000 (551-1793)	274 (150-510)	222 (121-415)
**Vanuatu**	259 (146-458)	7326 (4136-12986)	1353 (758-2425)	42 (22-126)	34 (18-102)
**Venezuela**	191 (106-339)	530593 (295687-942111)	1000 (551-1791)	2954 (1593-6553)	2394 (1281-5333)
**Viet Nam**	259 (147-460)	1844250 (1046715-3278031)	1353 (756-2419)	10797 (5688-34320)	8747 (4590-27785)
**Yemen**	191 (104-347)	593564 (325017-1079034)	996 (537-1824)	3100 (1671-5679)	2505 (1347-4615)
**Zambia**	438 (257-747)	817804 (478902-1393559)	1980 (1150-3414)	134456 (76937-233500)	108548 (61775-189297)
**Zimbabwe**	339 (198-579)	613876 (358127-1047587)	1405 (813-2434)	156959 (89530-276565)	126760 (72075-224531)

Supplementary table 5: Incidence and episodes of severe pneumonia in children younger than 5 years in 2000

Country	Incidence of severe pneumonia (per 1000 children per year) in children younger than 5 years	Episodes of severe pneumonia in children younger than 5 years	Incidence of severe pneumonia in HIV infected children (per 1000 children per year)	Episode of severe pneumonia in HIV infected children	Episodes of severe pneumonia attributable to HIV
**Afghanistan**	95 (35-233)	407117 (148652-992565)	497 (181-1213)	2118 (773-5171)	1711 (624-4195)
**Algeria**	45 (17-101)	140737 (54214-313443)	236 (91-528)	740 (284-1678)	598 (229-1360)
**Angola**	53 (20-116)	143488 (55826-315880)	271 (105-603)	2244 (716-6148)	1817 (577-4979)
**Antigua and Barbuda**	30 (11-72)	236 (88-555)	159 (59-377)	1 (0-3)	1 (0-2)
**Argentina**	30 (11-71)	105073 (38910-246476)	160 (59-377)	557 (207-1336)	451 (167-1083)
**Bahamas**	31 (11-72)	858 (317-2013)	159 (59-377)	7 (2-21)	6 (2-17)
**Bahrain**	49 (17-121)	3516 (1259-8752)	253 (91-632)	18 (7-47)	15 (5-38)
**Bangladesh**	58 (23-128)	970148 (378020-2149954)	301 (116-669)	5036 (1949-11205)	4071 (1567-9077)
**Barbados**	30 (11-72)	563 (208-1324)	159 (59-377)	3 (1-7)	2 (1-6)
**Belize**	43 (16-97)	1463 (559-3339)	220 (83-506)	16 (5-44)	13 (4-36)
**Benin**	83 (32-189)	103760 (39250-235194)	430 (163-980)	1707 (532-4711)	1376 (428-3802)
**Bhutan**	35 (13-82)	2600 (959-6155)	182 (67-432)	14 (5-33)	11 (4-26)
**Bolivia**	44 (17-103)	54060 (20115-126020)	231 (86-543)	297 (109-753)	240 (88-611)
**Botswana**	70 (27-156)	15936 (6133-35350)	311 (119-696)	2970 (1138-6717)	2398 (917-5461)
**Brazil**	39 (14-94)	678996 (245811-1638881)	203 (73-493)	3676 (1316-9516)	2969 (1064-7732)
**Burkina Faso**	56 (21-125)	121027 (46372-272093)	282 (108-640)	4514 (1650-10948)	3646 (1329-8857)
**Burundi**	61 (23-135)	72867 (28042-161192)	311 (120-694)	2047 (743-5045)	1653 (597-4097)
**Cambodia**	73 (27-171)	109120 (40440-253904)	379 (140-888)	1074 (346-2900)	868 (278-2349)
**Cameroon**	39 (15-87)	106900 (40982-239230)	192 (74-434)	6797 (2353-17506)	5494 (1902-14244)
**Cape Verde**	52 (20-115)	3238 (1255-7110)	271 (104-604)	23 (8-71)	18 (6-58)
**Central African Republic**	86 (32-195)	50667 (19195-115462)	415 (157-951)	4322 (1604-10467)	3491 (1294-8457)

Country	Incidence of severe pneumonia (per 1000 children per year) in children younger than 5 years	Episodes of severe pneumonia in children younger than 5 years	Incidence of severe pneumonia in HIV infected children (per 1000 children per year)	Episode of severe pneumonia in HIV infected children	Episodes of severe pneumonia attributable to HIV
**Chad**	84 (31-200)	141824 (51626-336983)	426 (156-1024)	4771 (1579-12801)	3854 (1273-10372)
**Chile**	30 (11-72)	40565 (15081-95442)	159 (59-378)	214 (80-515)	173 (65-419)
**China**	42 (16-98)	3479385 (1302888-8200548)	217 (82-516)	20105 (7275-66755)	16292 (5876-54079)
**Colombia**	32 (12-76)	141751 (51964-338603)	167 (61-397)	812 (287-2245)	659 (233-1820)
**Comoros**	75 (28-169)	6391 (2396-14478)	388 (146-888)	38 (13-132)	30 (11-106)
**Congo**	34 (12-81)	17391 (6397-41409)	169 (62-403)	1182 (417-2919)	957 (337-2377)
**Cook Islands**	42 (16-98)	91 (34-215)	218 (82-516)	1 (0-2)	0 (0-2)
**Costa Rica**	30 (11-71)	12007 (4464-28160)	159 (59-377)	64 (24-152)	51 (19-124)
**Cote d'Ivoire**	51 (19-115)	133774 (50949-302183)	246 (93-560)	11791 (4255-28418)	9522 (3441-23087)
**Dem. Peoples's Rep. of Korea**	55 (21-122)	108473 (42180-242819)	284 (109-637)	563 (217-1264)	455 (174-1029)
**Dem. Rep. of the Congo**	66 (25-145)	585545 (224676-1286089)	341 (132-757)	9671 (3462-23971)	7806 (2796-19342)
**Djibouti**	31 (11-74)	3039 (1117-7281)	159 (58-383)	60 (20-155)	48 (16-126)
**Dominica**	30 (11-72)	212 (79-498)	159 (59-377)	1 (0-3)	1 (0-2)
**Dominican Republic**	30 (11-70)	30690 (11207-72350)	155 (57-367)	479 (96-2354)	388 (77-1913)
**Ecuador**	30 (11-71)	45739 (16954-107112)	159 (59-376)	247 (91-618)	200 (73-502)
**Egypt**	24 (8-60)	187682 (66308-467353)	127 (45-319)	994 (350-2489)	806 (283-2029)
**El Salvador**	42 (16-97)	32954 (12536-75067)	220 (83-507)	182 (68-457)	147 (54-371)
**Equatorial Guinea**	46 (18-103)	3926 (1517-8740)	236 (91-527)	99 (36-247)	80 (29-201)
**Eritrea**	60 (23-133)	40521 (15540-89563)	311 (120-696)	609 (207-1578)	492 (167-1283)
**Ethiopia**	55 (21-128)	684397 (254500-1578045)	275 (102-639)	39752 (14434-93770)	32094 (11694-76032)
**Fiji**	42 (16-99)	4093 (1534-9716)	218 (82-515)	24 (9-80)	19 (7-65)
**Gabon**	52 (20-120)	9535 (3609-21998)	262 (98-605)	385 (136-974)	312 (109-790)

Country	Incidence of severe pneumonia (per 1000 children per year) in children younger than 5 years	Episodes of severe pneumonia in children younger than 5 years	Incidence of severe pneumonia in HIV infected children (per 1000 children per year)	Episode of severe pneumonia in HIV infected children	Episodes of severe pneumonia attributable to HIV
**Gambia**	60 (23-133)	13868 (5346-30643)	312 (120-694)	112 (37-324)	91 (30-262)
**Ghana**	45 (17-103)	130056 (48700-295604)	232 (87-532)	4044 (1372-10508)	3266 (1105-8527)
**Grenada**	30 (11-72)	318 (118-748)	159 (59-375)	2 (1-4)	1 (1-3)
**Guatemala**	62 (24-138)	115625 (44268-256059)	323 (124-725)	616 (234-1446)	498 (187-1172)
**Guinea**	50 (19-111)	75504 (29397-167949)	259 (100-581)	1016 (341-2724)	821 (275-2214)
**Guinea-Bissau**	62 (24-137)	13261 (5093-29388)	311 (120-695)	540 (198-1297)	437 (160-1054)
**Guyana**	42 (16-97)	3710 (1414-8475)	221 (83-506)	23 (8-64)	19 (7-52)
**Haiti**	51 (18-118)	61833 (22539-143948)	255 (94-598)	2522 (888-6175)	2035 (714-5007)
**Honduras**	32 (12-74)	30479 (11692-69425)	168 (64-387)	350 (120-891)	283 (97-724)
**India**	59 (23-130)	7233149 (2813037-15998283)	305 (117-682)	37560 (14391-83832)	30312 (11615-67752)
**Indonesia**	78 (30-175)	1646114 (631674-3682083)	406 (155-916)	8541 (3261-19269)	6901 (2633-15675)
**Iran (Islamic Republic of)**	48 (17-121)	306182 (109807-763377)	253 (91-632)	1617 (574-4104)	1308 (462-3318)
**Iraq**	30 (11-73)	117867 (43210-281592)	159 (58-383)	614 (224-1481)	496 (181-1200)
**Jamaica**	31 (11-72)	8658 (3199-20308)	159 (59-377)	88 (27-264)	71 (22-215)
**Jordan**	45 (15-119)	31354 (10634-82751)	236 (80-621)	164 (55-432)	132 (45-349)
**Kenya**	64 (24-147)	329125 (123806-751818)	304 (114-700)	39024 (14282-92540)	31493 (11553-75008)
**Kiribati**	42 (16-98)	412 (154-975)	217 (82-513)	2 (1-9)	2 (1-7)
**Kuwait**	48 (17-121)	9034 (3238-22573)	254 (90-633)	48 (17-120)	39 (14-97)
**Lao People's Dem. Republic**	73 (27-170)	59599 (22027-138464)	379 (140-890)	310 (114-725)	250 (92-587)
**Lebanon**	48 (17-121)	14765 (5284-36933)	254 (90-633)	78 (28-197)	63 (23-160)
**Lesotho**	61 (23-137)	16537 (6286-37001)	261 (100-588)	3701 (1379-8472)	2989 (1117-6876)
**Liberia**	38 (14-92)	19038 (6899-45595)	196 (71-473)	561 (194-1456)	454 (157-1181)

Country	Incidence of severe pneumonia (per 1000 children per year) in children younger than 5 years	Episodes of severe pneumonia in children younger than 5 years	Incidence of severe pneumonia in HIV infected children (per 1000 children per year)	Episode of severe pneumonia in HIV infected children	Episodes of severe pneumonia attributable to HIV
**Libyan Arab Jamahiriya**	48 (17-121)	26549 (9525-66344)	254 (90-633)	140 (50-351)	113 (40-287)
**Madagascar**	73 (28-162)	208148 (79931-459199)	379 (146-844)	1550 (522-4691)	1252 (421-3798)
**Malawi**	64 (24-142)	130332 (49622-290099)	282 (107-635)	24023 (9121-54348)	19408 (7326-43970)
**Malaysia**	73 (27-170)	198915 (73544-462068)	379 (139-889)	1033 (379-2423)	834 (306-1963)
**Maldives**	33 (12-78)	1146 (429-2686)	176 (65-418)	6 (2-14)	5 (2-12)
**Mali**	58 (22-130)	111066 (42610-249128)	297 (114-670)	2172 (768-5469)	1754 (619-4431)
**Marshall Islands**	42 (16-99)	260 (97-616)	217 (81-518)	2 (1-6)	1 (0-5)
**Mauritania**	52 (20-115)	23108 (8967-50825)	271 (105-604)	150 (53-458)	121 (43-370)
**Mauritius**	45 (17-101)	4470 (1722-9954)	236 (90-528)	24 (9-54)	19 (7-44)
**Mexico**	30 (11-72)	374438 (139179-880680)	159 (59-377)	1969 (730-4693)	1595 (592-3814)
**Micronesia (Fed. States of)**	42 (16-98)	625 (234-1475)	218 (82-513)	4 (1-12)	3 (1-10)
**Mongolia**	42 (16-98)	9719 (3638-22952)	218 (82-515)	56 (20-184)	45 (16-150)
**Morocco**	42 (16-98)	131698 (49231-305515)	219 (82-513)	683 (254-1605)	552 (206-1303)
**Mozambique**	65 (25-143)	206782 (79284-455242)	322 (124-715)	13291 (4972-31162)	10737 (4014-25293)
**Myanmar**	57 (21-133)	274575 (102487-645791)	294 (109-693)	1472 (539-3583)	1187 (432-2903)
**Namibia**	60 (23-133)	16533 (6377-36608)	275 (106-612)	2442 (925-5570)	1974 (743-4520)
**Nauru**	42 (16-99)	50 (19-119)	217 (82-516)	0 (0-2)	0 (0-1)
**Nepal**	54 (20-124)	186708 (70958-429340)	280 (107-646)	974 (372-2251)	787 (302-1825)
**Nicaragua**	38 (14-87)	26628 (10083-61163)	196 (74-457)	139 (52-323)	112 (42-261)
**Niger**	83 (31-190)	187718 (71111-428909)	430 (162-990)	1614 (514-4711)	1304 (414-3813)
**Nigeria**	64 (24-148)	1345997 (501176-3125966)	325 (121-759)	40425 (14096-102675)	32663 (11367-83031)
**Niue**	74 (27-172)	17 (6-40)	380 (139-891)	0 (0-1)	0 (0-1)

Country	Incidence of severe pneumonia (per 1000 children per year) in children younger than 5 years	Episodes of severe pneumonia in children younger than 5 years	Incidence of severe pneumonia in HIV infected children (per 1000 children per year)	Episode of severe pneumonia in HIV infected children	Episodes of severe pneumonia attributable to HIV
**Oman**	48 (17-121)	13082 (4691-32610)	254 (90-635)	69 (25-176)	56 (20-143)
**Pakistan**	31 (11-74)	656533 (239498-1563404)	162 (59-391)	3409 (1238-8229)	2757 (997-6671)
**Palau**	73 (27-170)	168 (62-391)	380 (139-891)	1 (0-2)	1 (0-2)
**Panama**	30 (11-71)	10597 (3934-24870)	160 (59-377)	59 (21-155)	48 (17-125)
**Papua New Guinea**	42 (16-99)	35040 (13159-82973)	218 (82-516)	299 (87-1214)	242 (70-985)
**Paraguay**	42 (16-97)	30115 (11473-68722)	220 (83-509)	157 (59-362)	127 (48-294)
**Peru**	32 (12-73)	95220 (35890-220421)	165 (62-385)	501 (188-1179)	405 (152-960)
**Philippines**	84 (32-188)	894417 (342176-2010519)	435 (166-985)	4644 (1775-10539)	3748 (1429-8541)
**Qatar**	48 (17-121)	2687 (962-6733)	254 (90-635)	14 (5-36)	11 (4-29)
**Republic of Korea**	42 (16-98)	130133 (48651-307925)	218 (82-518)	753 (269-2517)	610 (218-2044)
**Rwanda**	38 (14-86)	63638 (23937-144541)	191 (72-435)	3121 (1138-7446)	2522 (920-6039)
**Saint Kitts and Nevis**	30 (11-71)	139 (51-325)	159 (59-376)	1 (0-2)	1 (0-1)
**Saint Lucia**	30 (11-71)	482 (179-1130)	160 (59-376)	3 (1-6)	2 (1-5)
**Saint Vincent and Grenadines**	30 (11-71)	334 (124-782)	159 (59-376)	2 (1-4)	1 (1-3)
**Samoa**	42 (16-98)	1064 (398-2510)	218 (82-516)	6 (2-21)	5 (2-17)
**Sao Tome and Principe**	53 (21-117)	1151 (445-2526)	271 (105-605)	32 (11-82)	26 (9-67)
**Saudi Arabia**	48 (17-121)	135676 (48634-340249)	254 (90-634)	716 (254-1810)	579 (205-1466)
**Senegal**	45 (17-99)	75203 (29188-167521)	231 (90-521)	497 (177-1388)	401 (142-1124)
**Seychelles**	45 (18-101)	329 (127-732)	236 (90-526)	2 (1-4)	1 (1-3)
**Sierra Leone**	62 (23-138)	43404 (16453-96736)	320 (122-722)	297 (105-858)	240 (84-692)
**Solomon Islands**	41 (16-98)	2684 (1005-6347)	217 (82-515)	16 (6-51)	13 (5-41)
**Somalia**	92 (34-216)	135230 (50096-317982)	477 (176-1134)	936 (320-2556)	756 (257-2074)

Country	Incidence of severe pneumonia (per 1000 children per year) in children younger than 5 years	Episodes of severe pneumonia in children younger than 5 years	Incidence of severe pneumonia in HIV infected children (per 1000 children per year)	Episode of severe pneumonia in HIV infected children	Episodes of severe pneumonia attributable to HIV
**South Africa**	76 (29-173)	365956 (139159-837157)	354 (134-814)	47044 (17592-110752)	37959 (14142-89811)
**Sri Lanka**	38 (14-91)	63237 (23527-151041)	199 (73-476)	330 (122-791)	267 (99-642)
**Sudan**	30 (11-73)	144352 (52859-345775)	159 (58-382)	753 (274-1816)	608 (222-1474)
**Suriname**	31 (11-72)	1536 (572-3616)	159 (59-375)	14 (4-43)	11 (4-35)
**Swaziland**	71 (27-157)	11100 (4267-24572)	311 (120-695)	2150 (816-4848)	1735 (657-3932)
**Syrian Arab Republic**	30 (11-73)	73157 (26740-174955)	159 (58-383)	381 (139-919)	308 (112-745)
**Thailand**	33 (12-84)	154295 (53731-392566)	172 (60-441)	914 (312-2395)	738 (252-1939)
**Timor-Leste**	52 (19-122)	9126 (3367-21332)	271 (100-638)	47 (17-111)	38 (14-90)
**Togo**	82 (31-190)	66210 (24740-152858)	409 (153-956)	3650 (1325-8930)	2951 (1070-7214)
**Tonga**	42 (16-98)	552 (208-1306)	217 (82-516)	3 (1-11)	3 (1-9)
**Trinidad and Tobago**	30 (11-72)	2732 (1014-6431)	159 (59-377)	20 (7-62)	16 (5-50)
**Tunisia**	44 (15-113)	36298 (12834-93728)	229 (81-594)	210 (72-726)	170 (58-589)
**Tuvalu**	42 (16-98)	48 (18-113)	218 (82-513)	0 (0-2)	0 (0-1)
**Uganda**	46 (18-102)	221571 (85327-494800)	223 (85-499)	19158 (7250-44480)	15474 (5822-36205)
**United Arab Emirates**	48 (17-121)	12549 (4494-31321)	254 (91-632)	66 (24-169)	54 (19-137)
**United Republic of Tanzania**	45 (18-100)	266222 (103940-590127)	213 (83-474)	31257 (11764-72110)	25280 (9445-58483)
**Uruguay**	30 (11-71)	8243 (3055-19361)	159 (59-376)	44 (16-105)	35 (13-85)
**Vanuatu**	42 (16-98)	1176 (441-2783)	218 (82-516)	7 (2-23)	6 (2-19)
**Venezuela**	30 (11-72)	84760 (31395-199655)	160 (59-376)	479 (173-1280)	388 (140-1044)
**Viet Nam**	42 (16-98)	295965 (111304-701490)	218 (82-514)	1767 (628-6283)	1432 (510-5087)
**Yemen**	30 (11-73)	94880 (34734-227396)	159 (58-382)	495 (180-1190)	400 (146-965)
**Zambia**	70 (27-159)	131366 (49971-296836)	319 (121-722)	21565 (8160-49867)	17407 (6567-40326)

Country	Incidence of severe pneumonia (per 1000 children per year) in children younger than 5 years	Episodes of severe pneumonia in children younger than 5 years	Incidence of severe pneumonia in HIV infected children (per 1000 children per year)	Episode of severe pneumonia in HIV infected children	Episodes of severe pneumonia attributable to HIV
**Zimbabwe**	54 (21-124)	98414 (37284-223784)	225 (86-512)	25084 (9485-58220)	20268 (7639-47294)

Supplementary table 6: Incidence and episodes of clinical pneumonia in children younger than 5 years in 2015

Country	Incidence of clinical pneumonia (per 1000 children per year) in children younger than 5 years	Episodes of clinical pneumonia in children younger than 5 years	Incidence of clinical pneumonia in HIV infected children (per 1000 children per year)	Episode of clinical pneumonia in HIV infected children	Episodes of clinical pneumonia attributable to HIV
**Afghanistan**	484 (268-895)	2395405 (1324586-4432668)	2511 (1381-4691)	12433 (6837-23222)	10057 (5479-18792)
**Algeria**	226 (136-380)	1039118 (623158-1742396)	1174 (698-1983)	5397 (3206-9149)	4364 (2582-7433)
**Angola**	158 (91-268)	743818 (429795-1263384)	817 (465-1400)	13166 (3832-36205)	10679 (3099-29498)
**Antigua and Barbuda**	127 (72-222)	923 (521-1613)	674 (373-1184)	5 (3-9)	4 (2-7)
**Argentina**	127 (72-221)	471297 (266785-823452)	673 (375-1180)	2503 (1394-4388)	2034 (1121-3590)
**Bahamas**	127 (72-221)	3695 (2090-6448)	673 (375-1183)	25 (13-55)	20 (10-45)
**Bahrain**	148 (74-293)	16058 (8047-31892)	782 (389-1559)	85 (42-170)	69 (34-139)
**Bangladesh**	277 (163-470)	4241015 (2498910-7206086)	1436 (835-2457)	22026 (12808-37702)	17808 (10269-30642)
**Barbados**	127 (72-222)	2201 (1242-3846)	674 (375-1184)	12 (7-21)	9 (5-17)
**Belize**	167 (99-279)	6592 (3916-11003)	871 (513-1475)	59 (28-124)	47 (22-100)
**Benin**	306 (185-509)	522886 (316293-869280)	1585 (951-2670)	4724 (2412-9162)	3819 (1935-7430)
**Bhutan**	175 (102-301)	11542 (6700-19839)	920 (526-1592)	62 (35-111)	50 (28-91)
**Bolivia**	123 (69-216)	146047 (81595-255661)	652 (360-1149)	773 (427-1367)	628 (344-1113)
**Botswana**	249 (152-414)	66395 (40437-110219)	1250 (752-2070)	3240 (1845-5684)	2618 (1483-4621)
**Brazil**	167 (99-279)	2503090 (1487643-4186797)	871 (511-1477)	13096 (7683-22216)	10594 (6166-18103)
**Burkina Faso**	275 (162-476)	865732 (509393-1496970)	1426 (827-2485)	7281 (3658-14423)	5881 (2937-11693)
**Burundi**	231 (138-391)	476026 (283527-805224)	1190 (696-2027)	7429 (3645-14528)	6002 (2936-11779)
**Cambodia**	255 (154-423)	451360 (273618-749415)	1326 (789-2218)	2640 (1504-5513)	2134 (1204-4472)
**Cameroon**	239 (143-400)	893933 (535403-1496571)	1201 (709-2017)	41921 (23804-72909)	33831 (19088-59196)
**Cape Verde**	221 (134-367)	11857 (7182-19658)	1150 (688-1908)	68 (39-147)	55 (31-120)
**Central African Republic**	227 (137-376)	160590 (97009-265981)	1150 (685-1909)	5816 (3152-10619)	4701 (2535-8625)

**Chad**	239 (129-437)	628909 (340011-1151351)	1223 (651-2260)	15758 (7397-32953)	12733 (5962-26735)
**Chile**	127 (72-221)	148432 (84084-258693)	673 (374-1182)	788 (438-1383)	640 (353-1129)
**China**	84 (40-166)	7024251 (3315052-13791976)	469 (217-929)	39027 (18022-77290)	32001 (14717-63656)
**Colombia**	125 (70-218)	466206 (262860-815495)	662 (367-1164)	2478 (1372-4379)	2013 (1107-3568)
**Comoros**	301 (174-532)	35902 (20744-63517)	1561 (895-2772)	189 (108-368)	153 (87-297)
**Congo**	225 (136-372)	170980 (103225-282490)	1150 (689-1903)	4536 (2407-8457)	3665 (1937-6844)
**Cook Islands**	285 (172-476)	599 (362-1000)	1482 (888-2494)	3 (2-5)	3 (1-4)
**Costa Rica**	133 (69-246)	46614 (24287-86255)	710 (363-1320)	249 (127-463)	202 (103-378)
**Cote d'Ivoire**	129 (73-226)	474218 (268115-827879)	673 (375-1183)	13820 (7140-26134)	11231 (5762-21334)
**Dem. Peoples's Rep. of Korea**	148 (74-293)	257929 (129840-512812)	782 (390-1561)	1367 (681-2728)	1109 (551-2228)
**Dem. Rep. of the Congo**	278 (163-471)	3854542 (2262964-6539301)	1437 (836-2459)	37763 (17560-79648)	30511 (14111-64499)
**Djibouti**	195 (117-325)	19864 (11912-33180)	1008 (599-1698)	334 (177-617)	270 (142-502)
**Dominica**	127 (72-222)	773 (437-1350)	673 (374-1183)	4 (2-7)	3 (2-6)
**Dominican Republic**	145 (79-264)	154361 (83906-280276)	770 (413-1410)	876 (454-2053)	711 (367-1670)
**Ecuador**	127 (72-221)	204113 (115402-356669)	673 (374-1186)	1085 (602-1910)	881 (485-1556)
**Egypt**	117 (64-209)	1414776 (775148-2537521)	627 (340-1132)	7602 (4114-13721)	6190 (3337-11234)
**El Salvador**	166 (99-278)	86519 (51377-144434)	870 (511-1475)	467 (271-872)	378 (219-707)
**Equatorial Guinea**	233 (140-391)	29912 (18006-50161)	1174 (700-1984)	1271 (705-2269)	1028 (567-1844)
**Eritrea**	241 (146-400)	196014 (119180-326040)	1252 (751-2078)	1126 (643-2341)	911 (517-1904)
**Ethiopia**	242 (147-402)	3539917 (2152704-5874567)	1252 (750-2078)	52893 (26480-101945)	42744 (21303-82705)
**Fiji**	162 (89-291)	14265 (7845-25647)	850 (462-1542)	75 (41-136)	61 (33-111)
**Gabon**	248 (142-433)	59385 (34058-103632)	1265 (718-2232)	1779 (896-3442)	1438 (718-2788)
**Gambia**	242 (147-400)	88597 (53835-146466)	1250 (748-2080)	905 (427-1864)	731 (344-1508)
**Ghana**	190 (114-315)	770760 (463959-1277691)	984 (584-1648)	11014 (4178-25716)	8906 (3364-20908)
**Grenada**	127 (72-222)	1260 (710-2201)	674 (373-1185)	7 (4-12)	5 (3-10)
**Guatemala**	167 (99-278)	348076 (206639-580886)	871 (511-1476)	1926 (1111-3784)	1561 (894-3067)
**Guinea**	244 (145-413)	499544 (296745-844625)	1258 (738-2144)	7226 (3469-14462)	5832 (2795-11750)
**Guinea-Bissau**	248 (151-411)	71594 (43475-118794)	1251 (748-2077)	2958 (1660-5232)	2387 (1336-4250)

**Guyana**	228 (136-385)	15322 (9100-25866)	1184 (695-2018)	140 (58-406)	113 (47-330)
**Haiti**	226 (133-383)	279997 (164390-473784)	1167 (674-1995)	4065 (1935-8206)	3286 (1553-6657)
**Honduras**	166 (98-281)	135049 (79884-229016)	862 (501-1483)	714 (414-1268)	577 (332-1029)
**India**	352 (206-611)	43567384 (25429504-75589014)	1830 (1057-3182)	226344 (130818-393623)	182810 (105223-319849)
**Indonesia**	326 (196-545)	8106927 (4884066-13560739)	1697 (1015-2870)	42504 (25297-72719)	34362 (20296-59071)
**Iran (Islamic Republic of)**	148 (74-293)	1013570 (507690-2010985)	782 (391-1563)	5367 (2680-10716)	4354 (2162-8720)
**Iraq**	193 (115-321)	1104387 (660330-1839203)	1007 (599-1699)	5768 (3432-9731)	4668 (2751-7913)
**Jamaica**	127 (72-221)	25874 (14613-45104)	673 (374-1183)	167 (86-363)	135 (69-295)
**Jordan**	148 (74-294)	144730 (72863-287787)	782 (390-1561)	767 (383-1530)	622 (310-1248)
**Kenya**	234 (140-391)	1679839 (1004783-2800621)	1187 (699-1997)	60951 (32562-111733)	49262 (26239-90882)
**Kiribati**	163 (91-285)	2399 (1350-4210)	855 (474-1505)	13 (7-22)	10 (6-18)
**Kuwait**	148 (74-293)	51420 (25781-102028)	782 (389-1561)	272 (135-543)	221 (109-442)
**Lao People's Dem. Republic**	362 (205-638)	303412 (172077-534900)	1876 (1051-3314)	1578 (885-2826)	1275 (711-2283)
**Lebanon**	148 (74-294)	68089 (34236-135376)	782 (390-1562)	361 (180-720)	293 (145-586)
**Lesotho**	262 (158-438)	73010 (44022-122030)	1151 (687-1926)	14278 (8389-24441)	11532 (6735-19853)
**Liberia**	206 (117-353)	144612 (82091-247522)	1068 (599-1842)	1527 (682-3258)	1234 (548-2636)
**Libyan Arab Jamahiriya**	148 (74-294)	95809 (48082-190747)	782 (391-1564)	508 (254-1015)	412 (205-826)
**Madagascar**	194 (116-326)	729961 (435981-1228874)	1007 (592-1705)	3950 (2285-7288)	3194 (1836-5923)
**Malawi**	221 (132-375)	653748 (390018-1106792)	1078 (635-1838)	53271 (31140-92137)	43037 (25002-74811)
**Malaysia**	159 (87-286)	393096 (216260-708730)	842 (456-1528)	2088 (1128-3791)	1697 (913-3087)
**Maldives**	168 (96-290)	6289 (3596-10841)	892 (504-1550)	33 (19-58)	27 (15-47)
**Mali**	293 (176-494)	958538 (574186-1614397)	1517 (896-2574)	8167 (3968-16735)	6604 (3185-13584)
**Marshall Islands**	237 (131-433)	1191 (657-2176)	1235 (673-2288)	6 (3-12)	5 (3-9)
**Mauritania**	221 (133-367)	132968 (80109-220504)	1150 (684-1913)	718 (420-1388)	580 (337-1122)
**Mauritius**	226 (136-380)	15989 (9636-26812)	1175 (699-1978)	83 (49-142)	67 (40-115)
**Mexico**	127 (72-222)	1472309 (832716-2574787)	674 (374-1182)	7826 (4350-13744)	6352 (3503-11245)
**Micronesia (Fed. States of)**	224 (126-408)	2597 (1463-4720)	1168 (648-2130)	14 (7-25)	11 (6-20)

**Mongolia**	131 (68-240)	44238 (23139-81220)	685 (353-1268)	232 (120-429)	187 (96-349)
**Morocco**	193 (116-321)	659275 (396184-1098142)	1007 (599-1697)	3446 (2048-5806)	2788 (1647-4718)
**Mozambique**	276 (166-462)	1328171 (801551-2227055)	1348 (805-2270)	100838 (58812-174572)	81485 (47150-141734)
**Myanmar**	286 (162-502)	1305054 (740564-2290541)	1481 (830-2620)	7599 (4056-16404)	6137 (3263-13245)
**Namibia**	245 (147-414)	82857 (49778-139972)	1179 (700-2018)	7910 (4498-14055)	6397 (3619-11439)
**Nauru**	142 (69-300)	136 (66-288)	758 (367-1595)	1 (0-2)	1 (0-2)
**Nepal**	259 (153-436)	725981 (429986-1225106)	1343 (782-2296)	3805 (2218-6805)	3075 (1782-5520)
**Nicaragua**	166 (99-278)	100819 (59931-168516)	871 (511-1478)	527 (309-896)	427 (249-730)
**Niger**	300 (173-521)	1243350 (715895-2157346)	1554 (885-2723)	6496 (3693-11453)	5248 (2965-9318)
**Nigeria**	302 (160-538)	9401585 (4984868-16747235)	1541 (805-2760)	266475 (135793-483365)	215309 (109239-391682)
**Niue**	158 (88-284)	22 (12-39)	819 (444-1476)	1 (0-2)	1 (0-1)
**Oman**	148 (74-293)	56952 (28579-112875)	781 (390-1558)	301 (150-600)	244 (122-490)
**Pakistan**	289 (170-488)	7120514 (4202740-12028777)	1497 (880-2556)	36929 (21693-63041)	29834 (17428-51048)
**Palau**	169 (94-299)	337 (188-598)	888 (492-1589)	2 (1-3)	1 (1-3)
**Panama**	127 (72-221)	46697 (26361-81559)	674 (374-1181)	250 (139-449)	203 (111-367)
**Papua New Guinea**	213 (127-361)	212031 (126127-359379)	1108 (653-1899)	1723 (828-3730)	1392 (666-3024)
**Paraguay**	167 (99-279)	112274 (66675-187900)	871 (510-1476)	588 (344-1008)	475 (277-817)
**Peru**	127 (72-219)	382408 (218525-661697)	670 (375-1177)	2025 (1133-3563)	1643 (912-2892)
**Philippines**	288 (168-517)	3244633 (1893117-5822399)	1500 (860-2691)	16888 (9684-30294)	13646 (7779-24537)
**Qatar**	148 (74-293)	19550 (9826-38823)	782 (390-1560)	104 (52-206)	84 (42-169)
**Republic of Korea**	137 (73-251)	312376 (167220-573186)	728 (382-1341)	1666 (875-3071)	1356 (701-2514)
**Rwanda**	154 (90-264)	261327 (152141-447199)	799 (458-1381)	2730 (1254-5704)	2207 (1011-4630)
**Saint Kitts and Nevis**	127 (72-221)	581 (329-1014)	673 (376-1184)	3 (2-5)	3 (1-4)
**Saint Lucia**	127 (72-221)	1745 (986-3045)	673 (373-1181)	9 (5-16)	8 (4-13)
**Saint Vincent and Grenadines**	127 (71-222)	1080 (606-1887)	674 (374-1184)	6 (3-10)	5 (3-8)
**Samoa**	153 (80-282)	3693 (1933-6808)	800 (414-1493)	19 (10-36)	16 (8-29)

**Sao Tome and Principe**	206 (124-345)	6098 (3663-10215)	1054 (625-1773)	137 (51-315)	111 (41-257)
**Saudi Arabia**	148 (74-294)	466829 (234362-928317)	782 (390-1560)	2473 (1234-4931)	2007 (995-4021)
**Senegal**	251 (150-423)	652553 (390649-1101261)	1303 (771-2225)	3636 (2093-7533)	2940 (1680-6112)
**Seychelles**	226 (136-380)	1897 (1143-3184)	1175 (699-1983)	10 (6-17)	8 (5-14)
**Sierra Leone**	239 (142-400)	239822 (142572-401208)	1234 (725-2078)	2682 (1358-5221)	2166 (1092-4221)
**Solomon Islands**	185 (107-323)	15154 (8768-26425)	962 (551-1690)	79 (45-138)	64 (36-112)
**Somalia**	464 (258-834)	914247 (507717-1643265)	2403 (1318-4358)	5717 (2881-12516)	4623 (2315-10166)
**South Africa**	233 (141-387)	1252624 (757150-2077824)	1150 (687-1910)	86427 (49393-151936)	69776 (39616-123689)
**Sri Lanka**	193 (111-340)	316629 (182203-559399)	1007 (575-1789)	1654 (945-2939)	1336 (757-2388)
**Sudan**	193 (116-320)	1146911 (688367-1907236)	1008 (599-1695)	6018 (3575-10205)	4871 (2870-8299)
**Suriname**	127 (72-221)	6107 (3451-10658)	674 (374-1180)	33 (18-59)	27 (15-49)
**Swaziland**	176 (101-307)	30585 (17556-53261)	804 (453-1410)	4871 (2643-8850)	3939 (2122-7186)
**Syrian Arab Republic**	148 (74-294)	323640 (162375-643845)	782 (389-1560)	1714 (853-3419)	1391 (691-2785)
**Thailand**	167 (88-312)	635279 (333865-1186013)	873 (452-1644)	3317 (1717-6247)	2682 (1385-5060)
**Timor-Leste**	266 (142-478)	54259 (28914-97422)	1383 (731-2494)	282 (149-508)	228 (120-412)
**Togo**	245 (148-406)	283704 (172031-471354)	1251 (748-2081)	7112 (3826-13055)	5749 (3072-10587)
**Tonga**	149 (80-267)	1918 (1029-3443)	784 (414-1411)	10 (5-18)	8 (4-15)
**Trinidad and Tobago**	127 (72-221)	12185 (6888-21254)	673 (374-1181)	72 (39-153)	59 (31-125)
**Tunisia**	148 (74-293)	145095 (72832-288135)	782 (389-1557)	768 (382-1529)	623 (310-1247)
**Tuvalu**	132 (66-252)	133 (67-253)	698 (343-1341)	1 (0-2)	1 (0-2)
**Uganda**	232 (141-384)	1687729 (1024842-2795560)	1158 (694-1931)	91625 (51799-159997)	74024 (41628-129982)
**United Arab Emirates**	148 (74-293)	72595 (36473-144187)	782 (390-1563)	384 (192-768)	312 (155-625)
**United Republic of Tanzania**	255 (153-426)	2397937 (1437770-4003238)	1291 (764-2169)	86882 (47941-155753)	70117 (38591-126297)
**Uruguay**	127 (72-221)	30533 (17238-53327)	674 (375-1185)	162 (90-286)	132 (72-234)
**Vanuatu**	233 (132-411)	8091 (4574-14278)	1207 (675-2145)	42 (23-75)	34 (19-61)
**Venezuela**	127 (72-221)	375058 (212331-654559)	674 (373-1184)	2005 (1109-3581)	1629 (895-2925)
**Viet Nam**	240 (133-427)	1859015 (1025819-3304844)	1248 (679-2247)	9716 (5272-17648)	7855 (4232-14345)
**Yemen**	193 (116-321)	756698 (455188-1261711)	1007 (599-1697)	3952 (2352-6658)	3197 (1891-5418)

**Zambia**	289 (173-490)	824264 (491788-1396281)	1395 (818-2382)	78276 (44604-137539)	63280 (35816-111346)
**Zimbabwe**	242 (145-407)	606343 (364290-1018681)	1157 (686-1953)	62790 (36284-109647)	50731 (29080-89295)

Supplementary table 7: Incidence and episodes of severe pneumonia in children younger than 5 years in 2015

Country	Incidence of severe pneumonia (per 1000 children per year) in children younger than 5 years	Episodes of severe pneumonia in children younger than 5 years	Incidence of severe pneumonia in HIV infected children (per 1000 children per year)	Episode of severe pneumonia in HIV infected children	Episodes of severe pneumonia attributable to HIV
**Afghanistan**	78 (29-188)	384260 (142964-928264)	404 (149-981)	2001 (737-4856)	1617 (595-3939)
**Algeria**	36 (14-80)	166754 (64459-368296)	189 (73-422)	868 (335-1945)	700 (270-1583)
**Angola**	25 (10-58)	118577 (45455-271754)	130 (50-301)	2074 (499-7252)	1682 (404-5896)
**Antigua and Barbuda**	20 (8-47)	147 (56-342)	108 (40-253)	1 (0-2)	1 (0-2)
**Argentina**	20 (8-47)	75398 (28340-175130)	108 (40-253)	400 (149-940)	325 (121-768)
**Bahamas**	20 (8-47)	591 (222-1371)	108 (40-253)	4 (1-11)	3 (1-9)
**Bahrain**	24 (8-60)	2560 (904-6523)	124 (44-319)	14 (5-35)	11 (4-28)
**Bangladesh**	44 (17-99)	677348 (259359-1518390)	230 (87-519)	3524 (1337-7958)	2849 (1078-6470)
**Barbados**	20 (8-47)	352 (132-818)	108 (40-253)	2 (1-4)	2 (1-4)
**Belize**	27 (10-60)	1054 (408-2359)	139 (53-314)	9 (3-25)	8 (3-20)
**Benin**	49 (19-108)	83873 (32569-184835)	254 (99-567)	756 (267-1895)	610 (215-1538)
**Bhutan**	28 (11-64)	1857 (702-4236)	148 (56-340)	10 (4-23)	8 (3-19)
**Bolivia**	20 (7-46)	23375 (8781-54582)	104 (39-246)	124 (46-293)	101 (37-237)
**Botswana**	40 (16-88)	10647 (4141-23540)	201 (78-445)	519 (196-1198)	419 (158-967)
**Brazil**	27 (10-60)	399901 (155019-897109)	139 (53-315)	2092 (803-4743)	1693 (649-3849)
**Burkina Faso**	44 (17-100)	138710 (52841-313146)	228 (87-519)	1170 (410-2987)	946 (330-2421)
**Burundi**	37 (14-83)	76281 (29252-171157)	190 (73-430)	1180 (413-3002)	954 (334-2436)
**Cambodia**	41 (16-90)	72438 (27985-159135)	213 (82-470)	431 (159-1132)	348 (129-917)
**Cameroon**	38 (15-86)	143164 (55211-320223)	192 (74-433)	6737 (2513-15570)	5442 (2025-12593)
**Cape Verde**	35 (14-79)	1898 (738-4227)	184 (71-409)	11 (4-29)	9 (3-24)

**Central African Republic**	36 (14-81)	25731 (9994-57065)	184 (71-410)	927 (345-2209)	750 (279-1798)
**Chad**	38 (14-92)	100662 (37096-241302)	196 (72-472)	2513 (848-6650)	2033 (684-5414)
**Chile**	20 (8-47)	23699 (8933-55168)	108 (40-253)	126 (47-296)	103 (38-242)
**China**	14 (5-34)	1123550 (376390-2866461)	75 (25-193)	6249 (2067-16036)	5125 (1690-13244)
**Colombia**	20 (7-46)	74528 (28018-173341)	106 (40-249)	397 (148-935)	322 (120-762)
**Comoros**	48 (18-111)	5780 (2205-13249)	251 (96-583)	30 (12-73)	25 (9-59)
**Congo**	36 (14-80)	27333 (10604-60758)	184 (71-410)	725 (259-1770)	587 (210-1438)
**Cook Islands**	46 (18-101)	96 (37-212)	238 (93-532)	1 (0-1)	0 (0-1)
**Costa Rica**	21 (8-52)	7484 (2647-18132)	114 (40-279)	40 (14-98)	32 (11-80)
**Cote d'Ivoire**	21 (8-48)	75899 (28583-176202)	108 (40-253)	2202 (797-5534)	1790 (644-4510)
**Dem. Peoples's Rep. of Korea**	24 (8-60)	41143 (14489-104759)	125 (44-321)	218 (77-560)	177 (62-456)
**Dem. Rep. of the Congo**	44 (17-100)	615657 (235185-1384938)	230 (87-521)	6040 (2039-16057)	4879 (1649-13003)
**Djibouti**	31 (12-70)	3188 (1237-7108)	162 (63-364)	53 (19-131)	43 (16-106)
**Dominica**	20 (8-47)	124 (46-287)	108 (40-253)	1 (0-2)	1 (0-1)
**Dominican Republic**	23 (9-56)	24649 (9114-59129)	123 (45-296)	142 (50-390)	115 (41-318)
**Ecuador**	20 (8-47)	32665 (12310-75813)	108 (40-253)	173 (65-407)	141 (53-332)
**Egypt**	19 (7-44)	226501 (84820-538621)	100 (37-241)	1217 (448-2920)	990 (365-2393)
**El Salvador**	27 (10-60)	13845 (5372-31005)	139 (53-314)	75 (29-179)	61 (23-146)
**Equatorial Guinea**	37 (14-83)	4806 (1853-10647)	189 (73-424)	204 (77-477)	165 (62-388)
**Eritrea**	39 (15-85)	31388 (12172-69506)	201 (78-445)	184 (69-459)	148 (55-373)
**Ethiopia**	39 (15-86)	567035 (220985-1255877)	201 (78-445)	8365 (2929-20798)	6768 (2360-16901)
**Fiji**	26 (10-61)	2295 (848-5351)	136 (51-325)	12 (4-29)	10 (4-23)
**Gabon**	40 (15-92)	9533 (3611-21972)	203 (77-470)	284 (100-700)	230 (81-568)
**Gambia**	39 (15-86)	14165 (5527-31333)	200 (78-445)	144 (49-381)	117 (39-310)
**Ghana**	30 (12-68)	123352 (47930-274980)	157 (61-352)	1752 (501-5214)	1416 (405-4213)
**Grenada**	20 (8-47)	202 (76-468)	108 (40-253)	1 (0-3)	1 (0-2)
**Guatemala**	27 (10-60)	55726 (21549-124796)	139 (53-314)	312 (118-769)	253 (95-625)
**Guinea**	39 (15-88)	79935 (30790-180036)	202 (78-456)	1159 (390-3009)	938 (314-2437)

**Guinea-Bissau**	40 (15-88)	11468 (4471-25345)	200 (78-446)	474 (177-1108)	383 (143-899)
**Guyana**	37 (14-82)	2464 (946-5528)	190 (73-431)	22 (7-80)	18 (5-65)
**Haiti**	36 (14-82)	44982 (17204-101564)	188 (72-426)	648 (218-1688)	524 (176-1375)
**Honduras**	26 (10-60)	21593 (8295-48728)	138 (53-313)	115 (44-264)	93 (35-214)
**India**	57 (22-127)	6993653 (2661243-15765551)	293 (111-671)	36296 (13779-83009)	29328 (11098-67412)
**Indonesia**	52 (20-115)	1301069 (498847-2869919)	272 (105-607)	6809 (2618-15351)	5505 (2104-12469)
**Iran (Islamic Republic of)**	24 (8-60)	161453 (56768-410399)	125 (44-320)	855 (302-2195)	694 (245-1786)
**Iraq**	31 (12-69)	177196 (68967-395165)	162 (63-362)	926 (359-2075)	749 (290-1689)
**Jamaica**	20 (8-47)	4139 (1556-9591)	108 (40-252)	27 (9-73)	22 (8-60)
**Jordan**	24 (8-60)	23102 (8124-58962)	124 (44-320)	122 (43-314)	99 (35-256)
**Kenya**	38 (15-84)	269243 (104272-600469)	191 (74-429)	9755 (3599-23550)	7889 (2899-19073)
**Kiribati**	26 (10-61)	385 (143-895)	137 (51-322)	2 (1-5)	2 (1-4)
**Kuwait**	24 (8-60)	8193 (2874-20821)	125 (44-319)	43 (15-111)	35 (12-90)
**Lao People's Dem. Republic**	58 (22-134)	48715 (18218-112476)	302 (112-708)	254 (94-598)	205 (76-486)
**Lebanon**	24 (8-60)	10859 (3826-27682)	125 (44-320)	57 (20-148)	47 (16-120)
**Lesotho**	42 (16-93)	11717 (4549-26007)	185 (71-412)	2289 (873-5157)	1851 (704-4184)
**Liberia**	33 (12-75)	23094 (8762-52581)	170 (64-392)	243 (80-662)	196 (64-536)
**Libyan Arab Jamahiriya**	24 (8-60)	15280 (5372-38936)	125 (44-320)	81 (29-207)	66 (23-169)
**Madagascar**	31 (12-69)	116583 (44986-261906)	161 (62-363)	635 (240-1490)	514 (193-1211)
**Malawi**	35 (14-79)	104755 (40457-233850)	173 (66-388)	8518 (3244-19331)	6886 (2624-15700)
**Malaysia**	25 (9-61)	63120 (23495-150520)	135 (50-324)	334 (123-804)	271 (100-655)
**Maldives**	27 (10-62)	1007 (384-2325)	143 (54-332)	5 (2-12)	4 (2-10)
**Mali**	47 (18-105)	153660 (59481-342394)	243 (94-547)	1312 (455-3426)	1061 (366-2788)
**Marshall Islands**	38 (14-91)	191 (70-455)	198 (73-479)	1 (0-2)	1 (0-2)
**Mauritania**	35 (14-79)	21288 (8273-47320)	184 (71-411)	116 (45-279)	94 (36-226)
**Mauritius**	36 (14-81)	2562 (991-5696)	189 (73-423)	13 (5-30)	11 (4-25)
**Mexico**	20 (8-47)	235595 (88526-547638)	108 (40-252)	1252 (467-2939)	1017 (378-2397)

**Micronesia (Fed. States of)**	36 (13-85)	417 (155-981)	188 (69-447)	2 (1-5)	2 (1-4)
**Mongolia**	21 (8-51)	7088 (2577-17182)	109 (39-267)	37 (13-90)	30 (11-73)
**Morocco**	31 (12-69)	105827 (41146-235471)	162 (63-362)	554 (214-1240)	448 (173-1009)
**Mozambique**	44 (17-98)	213124 (82285-471772)	216 (84-485)	16227 (6147-36881)	13116 (4955-29918)
**Myanmar**	46 (17-106)	209183 (77752-483656)	238 (88-552)	1233 (446-3312)	996 (359-2688)
**Namibia**	39 (15-88)	13301 (5176-29757)	190 (73-426)	1272 (482-2929)	1027 (387-2378)
**Nauru**	23 (8-61)	22 (7-58)	121 (41-325)	0 (0-0)	0 (0-0)
**Nepal**	41 (16-92)	115876 (44604-259539)	215 (82-486)	611 (232-1416)	494 (188-1148)
**Nicaragua**	27 (10-60)	16144 (6226-36164)	139 (53-314)	84 (32-191)	68 (26-155)
**Niger**	48 (18-109)	198793 (75021-451921)	249 (93-570)	1040 (391-2404)	841 (314-1946)
**Nigeria**	48 (18-113)	1500575 (547605-3516956)	245 (89-581)	42340 (15306-103084)	34202 (12350-83467)
**Niue**	25 (9-60)	3 (1-8)	131 (48-313)	0 (0-0)	0 (0-0)
**Oman**	24 (8-60)	9074 (3198-23045)	125 (44-320)	48 (17-123)	39 (14-101)
**Pakistan**	46 (18-103)	1140680 (440718-2552577)	241 (93-544)	5937 (2290-13408)	4794 (1850-10874)
**Palau**	27 (10-64)	54 (20-128)	143 (53-339)	0 (0-1)	0 (0-1)
**Panama**	20 (8-47)	7468 (2815-17298)	108 (40-252)	40 (15-95)	33 (12-77)
**Papua New Guinea**	34 (13-77)	34224 (13026-76732)	179 (68-404)	275 (93-752)	222 (75-611)
**Paraguay**	27 (10-60)	17940 (6950-40317)	139 (54-315)	94 (36-213)	76 (29-173)
**Peru**	20 (8-47)	61258 (22882-142437)	107 (40-250)	323 (122-758)	262 (98-617)
**Philippines**	46 (18-107)	522829 (198457-1204693)	241 (91-562)	2718 (1026-6342)	2196 (825-5146)
**Qatar**	24 (8-60)	3114 (1098-7956)	125 (44-321)	16 (6-42)	13 (5-35)
**Republic of Korea**	22 (8-53)	50195 (18071-120925)	117 (42-284)	267 (96-648)	217 (78-529)
**Rwanda**	25 (9-56)	41664 (15875-95655)	127 (48-293)	435 (145-1135)	352 (117-922)
**Saint Kitts and Nevis**	20 (8-47)	93 (35-216)	108 (40-253)	0 (0-1)	0 (0-1)
**Saint Lucia**	20 (8-47)	279 (105-646)	108 (40-253)	1 (1-3)	1 (0-3)
**Saint Vincent and Grenadines**	20 (8-47)	173 (65-400)	108 (40-252)	1 (0-2)	1 (0-2)
**Samoa**	24 (9-59)	592 (214-1436)	128 (46-314)	3 (1-8)	3 (1-6)

**Sao Tome and Principe**	33 (13-74)	977 (377-2181)	169 (65-379)	22 (6-63)	18 (5-51)
**Saudi Arabia**	24 (8-60)	74471 (26204-189719)	125 (44-320)	394 (139-1012)	320 (113-823)
**Senegal**	40 (16-90)	104392 (40463-234065)	209 (81-468)	592 (222-1530)	478 (179-1234)
**Seychelles**	36 (14-80)	304 (118-674)	189 (72-423)	2 (1-4)	1 (0-3)
**Sierra Leone**	38 (15-86)	38365 (14758-86086)	198 (76-444)	431 (151-1089)	348 (121-885)
**Solomon Islands**	30 (11-68)	2440 (929-5538)	155 (59-356)	13 (5-29)	10 (4-24)
**Somalia**	74 (28-174)	146691 (54754-342779)	386 (142-910)	927 (320-2483)	749 (257-2006)
**South Africa**	37 (15-83)	200634 (78038-445634)	184 (72-410)	13883 (5267-32148)	11222 (4243-26089)
**Sri Lanka**	31 (12-71)	50981 (19092-117264)	162 (61-375)	266 (100-616)	215 (80-500)
**Sudan**	31 (12-69)	184135 (71551-410563)	162 (62-363)	966 (373-2189)	781 (302-1778)
**Suriname**	20 (8-47)	977 (368-2264)	108 (40-253)	5 (2-12)	4 (2-10)
**Swaziland**	28 (11-65)	4888 (1837-11302)	128 (48-299)	779 (287-1861)	630 (231-1514)
**Syrian Arab Republic**	24 (8-60)	51656 (18139-132000)	125 (44-320)	273 (97-701)	222 (78-572)
**Thailand**	27 (10-65)	101972 (36671-248355)	140 (50-344)	531 (190-1307)	429 (154-1060)
**Timor-Leste**	42 (15-100)	8623 (3129-20397)	220 (80-524)	45 (16-107)	36 (13-86)
**Togo**	39 (15-87)	45439 (17626-100510)	200 (78-445)	1134 (414-2716)	917 (334-2205)
**Tonga**	24 (9-57)	308 (111-733)	125 (45-300)	2 (1-4)	1 (0-3)
**Trinidad and Tobago**	20 (8-47)	1949 (732-4516)	108 (40-253)	12 (4-31)	10 (3-25)
**Tunisia**	24 (8-60)	23112 (8104-58931)	125 (44-320)	122 (43-315)	99 (35-257)
**Tuvalu**	21 (7-53)	21 (7-53)	111 (39-281)	0 (0-0)	0 (0-0)
**Uganda**	37 (14-83)	270471 (105347-600568)	186 (72-413)	14679 (5549-34031)	11864 (4483-27561)
**United Arab Emirates**	24 (8-60)	11571 (4082-29531)	125 (44-320)	61 (22-157)	50 (18-128)
**United Republic of Tanzania**	41 (16-91)	383430 (148445-855392)	207 (80-464)	13894 (5175-33123)	11221 (4172-26893)
**Uruguay**	20 (8-47)	4880 (1836-11317)	108 (40-252)	26 (10-61)	21 (8-50)
**Vanuatu**	37 (14-86)	1304 (488-2993)	195 (72-452)	7 (3-16)	5 (2-13)
**Venezuela**	20 (8-47)	60024 (22535-139189)	108 (40-252)	321 (120-756)	260 (97-617)
**Viet Nam**	39 (14-91)	298455 (109028-702805)	200 (73-474)	1557 (569-3699)	1257 (458-3001)
**Yemen**	31 (12-69)	121259 (47184-270659)	162 (63-363)	634 (246-1425)	513 (198-1159)

**Zambia**	47 (18-104)	132848 (51225-297764)	225 (87-506)	12610 (4746-29188)	10190 (3817-23583)
**Zimbabwe**	39 (15-86)	97432 (37808-216024)	186 (72-416)	10070 (3844-23272)	8131 (3095-18973)

Supplementary table 8: Decrease in incidence of clinical pneumonia in developing countries between 2000 and 2015 (in descending order)

Country	Estimated incidence of clinical pneumonia per 1000 children per year [2000]	Estimated incidence of clinical pneumonia per 1000 children per year [2015]	Reduction in incidence of clinical pneumonia between 2000 and 2015
China	259	84	68%
Niue	467	158	66%
Malaysia	460	159	65%
Palau	460	169	63%
Swaziland	441	176	60%
Cote d'Ivoire	315	129	59%
Central African Republic	534	227	57%
Madagascar	456	194	57%
Guatemala	388	167	57%
Dem. Peoples's Rep. of Korea	341	148	57%
Bolivia	277	123	56%
Chad	528	239	55%
Togo	513	245	52%
Angola	327	158	52%
Bahrain	302	148	51%
Iran (Islamic Republic of)	302	148	51%
Kuwait	302	148	51%
Lebanon	302	148	51%
Libyan Arab Jamahiriya	302	148	51%
Oman	302	148	51%
Qatar	302	148	51%
Saudi Arabia	302	148	51%
United Arab Emirates	302	148	51%
South Africa	473	233	51%
Mongolia	259	131	49%
Tuvalu	259	132	49%
Jordan	283	148	48%
Republic of Korea	259	137	47%
Tunisia	271	148	45%
Nauru	259	142	45%
Cambodia	462	255	45%
Philippines	521	288	45%
Malawi	396	221	44%
Botswana	437	249	43%
Tonga	259	149	42%
Niger	519	300	42%
Kenya	402	234	42%
Benin	520	306	41%

Samoa	258	153	41%
Burundi	380	231	39%
Sierra Leone	385	239	38%
Sao Tome and Principe	331	206	38%
Fiji	259	162	37%
El Salvador	265	166	37%
Belize	266	167	37%
Colombia	199	125	37%
Kiribati	259	163	37%
Paraguay	265	167	37%
Eritrea	376	241	36%
Peru	197	127	36%
Guinea-Bissau	384	248	35%
Comoros	466	301	35%
Gambia	374	242	35%
Rwanda	237	154	35%
Namibia	372	245	34%
Zambia	438	289	34%
Ghana	286	190	34%
Antigua and Barbuda	191	127	34%
Argentina	191	127	34%
Bahamas	191	127	34%
Barbados	191	127	34%
Chile	191	127	34%
Ecuador	191	127	34%
Grenada	191	127	34%
Jamaica	191	127	34%
Mexico	191	127	34%
Panama	191	127	34%
Saint Kitts and Nevis	191	127	34%
Saint Lucia	191	127	34%
Saint Vincent and Grenadines	191	127	34%
Suriname	191	127	34%
Trinidad and Tobago	191	127	34%
Venezuela	191	127	34%
Dominica	190	127	33%
Uruguay	190	127	33%
Indonesia	487	326	33%
Dem. Rep. of the Congo	413	278	33%
Cape Verde	325	221	32%
Mauritania	325	221	32%
Mozambique	405	276	32%
Brazil	244	167	32%
Lesotho	380	262	31%

Ethiopia	349	242	31%
Costa Rica	190	133	30%
Nicaragua	236	166	30%
Haiti	317	226	29%
Zimbabwe	339	242	29%
Solomon Islands	259	185	29%
Morocco	263	193	27%
Nigeria	401	302	25%
Bangladesh	361	277	23%
Gabon	323	248	23%
Nepal	336	259	23%
Egypt	151	117	23%
Syrian Arab Republic	191	148	23%
Dominican Republic	187	145	22%
Guinea	313	244	22%
Lao People's Dem. Republic	460	362	21%
Burkina Faso	349	275	21%
Mauritius	283	226	20%
Algeria	282	226	20%
Seychelles	282	226	20%
Somalia	576	464	19%
Thailand	207	167	19%
Uganda	287	232	19%
Afghanistan	598	484	19%
Mali	362	293	19%
Bhutan	216	175	19%
Myanmar	353	286	19%
Timor-Leste	328	266	19%
Maldives	207	168	19%
Equatorial Guinea	287	233	19%
Sri Lanka	237	193	19%
Papua New Guinea	259	213	18%
Honduras	201	166	17%
Liberia	240	206	14%
Guyana	265	228	14%
Micronesia (Fed. States of)	259	224	14%
Vanuatu	259	233	10%
Senegal	278	251	10%
United Republic of Tanzania	281	255	9%
Marshall Islands	259	237	8%
Viet Nam	259	240	7%
India	366	352	4%
Cameroon	241	239	1%
Djibouti	193	195	-1%

**Iraq**	191	193	-1%
**Sudan**	191	193	-1%
**Yemen**	191	193	-1%
**Congo**	210	225	-7%
**Cook Islands**	259	285	-10%
**Pakistan**	195	289	-48%

Supplementary table 9: Decrease in number of episodes of clinical pneumonia in developing countries between 2000 and 2015 (in descending order)

Country	Episodes of clinical pneumonia in 2000	Episodes of clinical pneumonia in 2015	Reduction in incidence of clinical pneumonia between 2000 and 2015 (%)
Niue	109	22	80%
Malaysia	1252567	393096	69%
Palau	1058	337	68%
China	21664371	7024251	68%
Dem. Peoples's Rep. of Korea	676397	257929	62%
Republic of Korea	809678	312376	61%
El Salvador	205855	86519	58%
Bolivia	337941	146047	57%
Nauru	312	136	56%
Swaziland	69222	30585	56%
Tuvalu	299	133	56%
Jamaica	54224	25874	52%
Guatemala	721079	348076	52%
Central African Republic	315982	160590	49%
Saint Vincent and Grenadines	2085	1080	48%
Colombia	886715	466206	47%
Iran (Islamic Republic of)	1909614	1013570	47%
South Africa	2287592	1252624	45%
Saudi Arabia	846478	466829	45%
Tonga	3443	1918	44%
Samoa	6615	3693	44%
Fiji	25507	14265	44%
Madagascar	1296879	729961	44%
Cote d'Ivoire	831445	474218	43%
Mauritius	27851	15989	43%
Saint Lucia	3020	1745	42%

**Libyan Arab Jamahiriya**	165612	95809	42%
**Philippines**	5569601	3244633	42%
**Dominica**	1324	773	42%
**Chile**	253993	148432	42%
**Brazil**	4256325	2503090	41%
**Cape Verde**	20141	11857	41%
**Uruguay**	51639	30533	41%
**Paraguay**	188106	112274	40%
**Nicaragua**	166240	100819	39%
**Costa Rica**	75133	46614	38%
**Nepal**	1164550	725981	38%
**Antigua and Barbuda**	1479	923	38%
**Barbados**	3523	2201	38%
**Mexico**	2343698	1472309	37%
**Grenada**	1993	1260	37%
**Suriname**	9634	6107	37%
**Tunisia**	225313	145095	36%
**Peru**	593405	382408	36%
**Thailand**	967387	635279	34%
**Rwanda**	397823	261327	34%
**Cambodia**	686893	451360	34%
**Guyana**	23147	15322	34%
**Botswana**	99258	66395	33%
**Micronesia (Fed. States of)**	3879	2597	33%
**Saint Kitts and Nevis**	867	581	33%
**Togo**	414104	283704	31%
**Bahamas**	5377	3695	31%
**Oman**	81486	56952	30%
**Bangladesh**	6041515	4241015	30%
**Panama**	66520	46697	30%
**Venezuela**	530593	375058	29%
**Chad**	889337	628909	29%

Syrian Arab Republic	457405	323640	29%
Bhutan	16262	11542	29%
Lesotho	102662	73010	29%
Honduras	189792	135049	29%
Trinidad and Tobago	17113	12185	29%
Ecuador	286484	204113	29%
Argentina	657436	471297	28%
Belize	9138	6592	28%
Haiti	387837	279997	28%
Mongolia	60535	44238	27%
Bahrain	21901	16058	27%
Marshall Islands	1620	1191	26%
Jordan	196313	144730	26%
Lebanon	92236	68089	26%
Myanmar	1715988	1305054	24%
Eritrea	252602	196014	22%
Indonesia	10234904	8106927	21%
Morocco	821577	659275	20%
Sri Lanka	393835	316629	20%
Dominican Republic	191800	154361	20%
Malawi	811482	653748	19%
Namibia	102839	82857	19%
Benin	647464	522886	19%
Lao People's Dem. Republic	374893	303412	19%
Kenya	2052821	1679839	18%
Ethiopia	4315381	3539917	18%
Angola	892424	743818	17%
Sao Tome and Principe	7162	6098	15%
Guinea-Bissau	82753	71594	13%
Maldives	7140	6289	12%
Sierra Leone	270598	239822	11%
Comoros	39824	35902	10%

**Solomon Islands**	16733	15154	9%
**Kuwait**	56363	51420	9%
**Seychelles**	2054	1897	8%
**Mauritania**	143839	132968	8%
**United Arab Emirates**	78282	72595	7%
**Kiribati**	2568	2399	7%
**Afghanistan**	2551075	2395405	6%
**Ghana**	816597	770760	6%
**Timor-Leste**	57165	54259	5%
**India**	45046369	43567384	3%
**Papua New Guinea**	217962	212031	3%
**Zimbabwe**	613876	606343	1%
**Gabon**	59423	59385	0%
**Zambia**	817804	824264	-1%
**Viet Nam**	1844250	1859015	-1%
**Gambia**	86442	88597	-2%
**Mozambique**	1287681	1328171	-3%
**Djibouti**	19012	19864	-4%
**Burundi**	455332	476026	-5%
**Dem. Rep. of the Congo**	3650795	3854542	-6%
**Guinea**	472902	499544	-6%
**Niger**	1173726	1243350	-6%
**Cook Islands**	565	599	-6%
**Somalia**	845515	914247	-8%
**Vanuatu**	7326	8091	-10%
**Nigeria**	8459265	9401585	-11%
**Burkina Faso**	757451	865732	-14%
**Qatar**	16773	19550	-17%
**Algeria**	877260	1039118	-18%
**Egypt**	1177828	1414776	-20%
**Liberia**	119385	144612	-21%
**Uganda**	1384676	1687729	-22%

**Equatorial Guinea**	24450	29912	-22%
**Sudan**	902948	1146911	-27%
**Yemen**	593564	756698	-27%
**Cameroon**	665451	893933	-34%
**Mali**	694702	958538	-38%
**Senegal**	467974	652553	-39%
**United Republic of Tanzania**	1655983	2397937	-45%
**Iraq**	736872	1104387	-50%
**Congo**	108072	170980	-58%
**Pakistan**	4099287	7120514	-74%

Supplementary table 10: Reduction in episodes of clinical pneumonia in HIV-infected children between 2000 and 2015 (in descending order)

Country	Estimated episodes in 2000	Estimated episodes in 2015	Reduction in clinical pneumonia in HIV-infected between 2000 and 2015 (%)
Rwanda	19478	2730	86%
Botswana	18501	3240	82%
Cote d'Ivoire	73607	13820	81%
Ethiopia	249513	52893	79%
Central African Republic	26995	5816	78%
Kenya	243421	60951	75%
Haiti	15817	4065	74%
Burkina Faso	28310	7281	74%
Dominican Republic	3106	876	72%
South Africa	293956	86427	71%
Eritrea	3819	1126	71%
Jamaica	557	167	70%
Togo	22939	7112	69%
China	123010	39027	68%
Malaysia	6490	2088	68%
Honduras	2177	714	67%
Palau	6	2	67%
Malawi	149884	53271	64%
Swaziland	13442	4871	64%
Republic of Korea	4591	1666	64%
Suriname	88	33	62%
Dem. Peoples's Rep. of Korea	3511	1367	61%
Cambodia	6749	2640	61%
Zimbabwe	156959	62790	60%
El Salvador	1128	467	59%
Madagascar	9540	3950	59%
Bolivia	1845	773	58%
Ghana	25458	11014	57%
Liberia	3517	1527	57%
Benin	10717	4724	56%
United Republic of Tanzania	194405	86882	55%
Colombia	5032	2478	51%
Cape Verde	138	68	51%
Nauru	2	1	50%

Niue	2	1	50%
Samoa	38	19	50%
Tonga	20	10	50%
Tuvalu	2	1	50%
Guatemala	3817	1926	50%
Fiji	146	75	49%
Namibia	15194	7910	48%
Chad	30044	15758	48%
Iran (Islamic Republic of)	10062	5367	47%
Saint Vincent and Grenadines	11	6	45%
Saudi Arabia	4451	2473	44%
Saint Lucia	16	9	44%
Mauritius	146	83	43%
Brazil	22998	13096	43%
Dominica	7	4	43%
Belize	103	59	43%
Burundi	12826	7429	42%
Trinidad and Tobago	124	72	42%
Thailand	5706	3317	42%
Bahamas	43	25	42%
Zambia	134456	78276	42%
Libyan Arab Jamahiriya	871	508	42%
Philippines	28893	16888	42%
Chile	1341	788	41%
Uruguay	274	162	41%
Tunisia	1296	768	41%
Mali	13650	8167	40%
Saint Kitts and Nevis	5	3	40%
Paraguay	978	588	40%
Nicaragua	866	527	39%
Congo	7323	4536	38%
Lesotho	23007	14278	38%
Dem. Rep. of the Congo	60481	37763	38%
Antigua and Barbuda	8	5	38%
Costa Rica	398	249	37%
Nepal	6069	3805	37%
Barbados	19	12	37%
Mexico	12343	7826	37%
Grenada	11	7	36%
Micronesia (Fed. States of)	22	14	36%
Niger	10130	6496	36%
Peru	3123	2025	35%
Marshall Islands	9	6	33%

Sao Tome and Principe	204	137	33%
Mongolia	343	232	32%
Venezuela	2954	2005	32%
Panama	366	250	32%
Oman	431	301	30%
Bangladesh	31396	22026	30%
Ecuador	1537	1085	29%
Syrian Arab Republic	2387	1714	28%
Argentina	3481	2503	28%
Bhutan	85	62	27%
Bahrain	115	85	26%
Gabon	2394	1779	26%
Lebanon	485	361	26%
Cook Islands	4	3	25%
Jordan	1021	767	25%
Uganda	119754	91625	23%
Mauritania	906	718	21%
Indonesia	53117	42504	20%
Sri Lanka	2054	1654	19%
Morocco	4272	3446	19%
Lao People's Dem. Republic	1945	1578	19%
Myanmar	9168	7599	17%
Comoros	228	189	17%
Solomon Islands	95	79	17%
Kiribati	15	13	13%
Maldives	38	33	13%
Guinea-Bissau	3368	2958	12%
Djibouti	375	334	11%
Viet Nam	10797	9716	10%
Seychelles	11	10	9%
Kuwait	296	272	8%
United Arab Emirates	413	384	7%
Angola	14149	13166	7%
Afghanistan	13240	12433	6%
Papua New Guinea	1830	1723	6%
Timor-Leste	297	282	5%
India	233949	226344	3%
Guyana	144	140	3%
Somalia	5839	5717	2%
Cameroon	42650	41921	2%
Vanuatu	42	42	0%
Nigeria	254032	266475	-5%
Guinea	6397	7226	-13%
Algeria	4590	5397	-18%

**Qatar**	88	104	-18%
**Senegal**	3053	3636	-19%
**Mozambique**	82950	100838	-22%
**Egypt**	6229	7602	-22%
**Yemen**	3100	3952	-27%
**Sudan**	4711	6018	-28%
**Gambia**	702	905	-29%
**Sierra Leone**	1821	2682	-47%
**Iraq**	3850	5768	-50%
**Pakistan**	21309	36929	-73%
**Equatorial Guinea**	624	1271	-104%

Supplementary table 11: List of hospital-based studies reporting hospitalisation rate for pneumonia in children younger than 5 years

Location (author)	Study period	Region	No. of hospitalisations	Population	Hospitalisation rate	LCI	UCI
**Kassena-Nankana District, Ghana[47]**	1990-91	Africa	30	1439	20.9	14.6	29.8
**Western Region, The Gambia[48]**	1993-96	Africa			8.0	7.5	8.6
**Soweto, South Africa[49]**	1998-2001	Africa	1134	41879.38	27.1	25.5	28.7
**Manhiça, Mozambique[7]**	1999-2000	Africa		6020	68.0	62.0	75.0
**Bamako, Mali[50]**	2000	Africa		200160	3.5	3.2	3.7
**Agincourt, South Africa[7]**	2000-01	Africa		8258	80.0	75.0	86.0
**Bondo district, Kenya[51]**	2001-03	Africa		52200	7.0	6.6	7.4
**Upper River Division and Central River Division, The Gambia[47]**	2002-04	Africa			10.3	8.9	11.9
**Kilifi District, Kenya[47]**	2002-08	Africa	5453	274075.4	19.9	19.4	20.4
**Manhiça district, Mozambique[47]**	2004-06	Africa			24.2	21.7	26.9
**Bondo district, Nyanza province, Kenya[47]**	2007-09	Africa	1694	100911.8389	16.8	16.0	17.6
**The Greater Banjul area and Upper River region, The Gambia[47]**	2008-09	Africa	898	27086	33.2	31.1	35.4
**Lwak, Kisumu, Kenya[47]**	2008-09	Africa	241	2437.63	98.9	87.1	112.2
**Soweto, South Africa[47]**	2009-10	Africa	1836	123574	14.9	14.2	15.6
**Lwak, Kisumu, Kenya[47]**	2009-10	Africa	177	2058.25	86.0	74.2	99.7
**American Indians and Alaska Natives, USA[52]**	1990-95	America	7641	678782	11.3	11.0	11.5
**Tennessee, USA[53]**	1995-2003	America			31.8	31.1	32.6
**USA[54]**	1997-99	America	569856	76903644	7.4	7.4	7.4
**USA[55]**	1996-98	America	2221	122369	18.2	17.4	18.9
**USA[56]**	1997-2000	America	232552	48127200	5.2	5.2	5.2

**American Indians and Alaska Natives, USA[57]**	1999-2001	America	11454	348486	32.9	32.3	33.5
**Colorado, USA[47]**	2000-08	America	50469	2993352	16.9	16.7	17.0
**Monroe County (NY), Davidson County (TN) and Hamilton County (OH), USA[58]**	2000-04	America		183839	15.2	14.7	15.8
**USA[54]**	2001-07	America	821914	141222337	5.8	5.8	5.8
**Paysandú and Salto, Uruguay[47]**	2001-04	America	2137	61950	34.5	33.1	36.0
**Yukon Kuskokwim Delta, Alaska, USA[47]**	2001-07	America			82.6	77.3	88.4
**Concordia and Parana, Argentina[47]**	2002-05	America	1601	37672	14.2	13.5	14.9
**San Lorenzo & Comitancillo, Guatemala[47]**	2002-04	America			18.4	11.8	28.9
**USA[59]**	2003	America			18.63	17.64	19.62
**Pilar, (Buenos Aires Province), Argentina[47]**	2003-05	America	856	81627	10.5	9.8	11.2
**USA[56]**	2003-06	America	232552	48127200	4.8	4.8	4.9
**San José, Costa Rica[47]**	2007-09	America			3.7	3.2	4.2
**Goiânia, Brazil[47]**	2007-09	America			21.6	20.7	22.6
**Multicentric, El Salvador[47]**	2007-08	America	31209	1114176	28.0	27.7	28.3
**Santa Rosa, Guatemala[47]**	2007-08	America	187	12700.6638	14.7	12.8	17.0
**Paysandú and Salto, Uruguay[47]**	2009	America	922	20650	44.7	41.9	47.6
**Colorado, USA[47]**	2009	America	6338	360188	17.6	17.2	18.0
**Santa Rosa, Guatemala[47]**	2009-10	America	676	21939.8487	30.8	28.6	33.2
**Quetzaltenango, Guatemala[47]**	2009-10	America	517	53033.336	9.8	8.9	10.6
**Yukon Kuskokwim Delta, Alaska, USA[47]**	2009-10	America			64.3	53.4	77.4

**Multicentric, El Salvador[47]**	2009-10	America	37931	1124341	33.7	33.4	34.1
**Multicentric, Pakistan[60]**	2002-03	Eastern Mediterranean	137	13364	10.3	8.7	12.1
**Karachi, Pakistan[61]**	2007-08	Eastern Mediterranean	58	3950	14.7	11.4	19.0
**Scotland, United Kingdom[62]**	1981-2005	Europe	16840	7679789	2.2	2.2	2.2
**Kuopio, Finland[63]**	1981-82	Europe	55	2917	18.9	14.5	24.6
**Spain[64]**	1995-96	Europe	15877	3213968	4.9	4.9	5.0
**Spain[65]**	1995-98	Europe	32123	7684928	4.2	4.1	4.2
**Valencia, Spain[66]**	1995-2000	Europe	23	3270	7.0	4.7	10.6
**Valencia, Spain[67]**	1995-2001	Europe	8181	1582398	5.2	5.1	5.3
**Gipuzoka, Spain[68]**	1996-2000	Europe	635	62800	10.1	9.4	10.9
**Kiel, Germany[69]**	1996-2000	Europe	367	53655	6.8	6.2	7.6
**Netherlands[70]**	1999-2000	Europe	3041	980986	3.1	3.0	3.2
**Multicentric, Germany[71]**	1999-2001	Europe			23.2	23.0	23.5
**Liguria, Italy[72]**	2000-04	Europe			4.1	3.8	4.5
**Gipuzoka, Spain[73]**	2004-07	Europe			20.7	19.0	22.5
**Netherlands[70]**	2006-07	Europe	3303	971471	3.4	3.3	3.5
**Limousin, France[74]**	2007-08	Europe			8.0	6.7	9.5
**Matlab, Bangladesh[75]**	1988-89	South-East Asia	27	503.39	53.6	36.8	78.2
**Kamalapur, Bangladesh[76]**	1999-2000	South-East Asia			40.5	30.0	54.9
**Lombok, Indonesia[47]**	1999-2002	South-East Asia			30.9	30.1	31.8
**Matlab, Bangladesh[77]**	1999-2001	South-East Asia	1250	24902	50.2	47.5	53.1
**Bhaktapur, Nepal[78]**	2004-07	South-East Asia			4.3	3.3	5.7
**Kathmandu, Nepal[79]**	2004-07	South-East Asia	967	150945	6.2	5.9	6.5

**Nakhon Phanom and Sa Kaeo provinces, Thailand [80]**	2005-10	South-East Asia	28543		57.7	57.1	58.4
**Mirzapur, Bangladesh[47]**	2004-08	South-East Asia	4444	41040	19.0	17.7	20.4
**Patan, Nepal[81]**	2005-06	South-East Asia	360	56875	6.3	5.7	7.0
**Multicentric, India[47]**	2005-07	South-East Asia			25.6	23.5	28.0
**Bangalore, India[82]**	2006	South-East Asia	967	150945	6.4	6.0	6.8
**Kamalapur, Bangladesh[47]**	2008	South-East Asia	109	4547.00229	24.0	19.9	28.9
**Multicentric, Bangladesh[47]**	2008	South-East Asia	96	6864	14.0	11.5	17.1
**Kamalapur, Bangladesh[47]**	2009-10	South-East Asia	151	7193.820435	20.99	17.9	24.62
**Multicentric, Bangladesh[47]**	2009	South-East Asia	186	14037	13.3	11.5	15.3
**Ballabgarh, Haryana, India[47]**	2009-10	South-East Asia	25	4840	3.7	2.6	5.2
**Alabang (Metro Manila), Philippines[83]**	1985-87	Western Pacific	34	1418.3	24.0	17.1	33.6
**Western Australia, Australia[84]**	1988-93	Western Pacific	7719	757610	10.2	10.0	10.4
**Zhejiang, China[85]**	1990-91	Western Pacific	64	7472	8.57	6.7	10.94
**Zhejiang, China[86]**	1990-91	Western Pacific	12	1215	9.88	5.61	17.39
**Heilongjiang, China[87]**	1991-93	Western Pacific	503	9901	50.8	46.55	55.44

**Shangdong, China[88]**	1992-93	Western Pacific	34	16751	2.03	1.45	2.84
**Chongqing, China[89]**	1992-93	Western Pacific	70	2246	31.17	24.66	39.39
**Heilongjiang, China[90]**	1993	Western Pacific	37	5812	6.37	4.61	8.79
**Auckland, New Zealand[91]**	1993-96	Western Pacific	548	50280	10.9	10.0	11.9
**Fujian, China[92]**	1994-95	Western Pacific	226	9323	24.24	21.28	27.62
**Henan, China[93]**	1994	Western Pacific	184	7917	23.24	20.11	26.85
**Jiangsu, China[94]**	1994-95	Western Pacific	110	11729	9.38	7.78	11.31
**Henan, China[95]**	1994-96	Western Pacific	1402	29590	47.38	44.96	49.93
**Fujian, China[96]**	1994-95	Western Pacific	112	4665	24.01	19.95	28.89
**Yunnan, China[97]**	1995-97	Western Pacific	1364	6966	195.81	185.69	206.48
**Shandong, China[98]**	1995-2001	Western Pacific	5823	375629	15.5	15.11	15.91
**Shangdong, China[99]**	1995-2004	Western Pacific	6916	537734	12.86	12.56	13.17
**Western Australia, Australia[100]**	1996-2005	Western Pacific	9358	360517	20.9	20.6	21.2
**Bohol, Philippines[47]**	2000-04	Western Pacific			33.2	30.3	36.4
**Tongatapu, Tonga[101]**	2000-04	Western Pacific		N/A	10.1	9.3	11.0
**Hong Kong SAR, China[102]**	2000-05	Western Pacific	18315	2131182	8.6	8.5	8.7

**Suva, Fiji**[103]	2001-02	Western Pacific	377.17	20954	18.0	16.3	19.9
**NhaTrang district, Vietnam**[104]	2005-06	Western Pacific	556	24641	22.6	20.8	24.5
**Tongatapu, Tonga**[101]	2006-07	Western Pacific		10322	7.2	6.1	8.5
**NhaTrang city, Vietnam**[105]	2007-08	Western Pacific	474	13941	34.0	31.1	37.2
**Biliran Island, The Philippines**[106]	2011-12	Western Pacific	94	4510	20.8		
**Kilifi District, Kenya**[107]	2007-10	Africa	603	45644	13.2	12.7	13.8
**Australia**[108]	1998-2004	Western Pacific	53452	7686007	7.0		
**Australia**[108]	2005-10	Western Pacific	33472	6734296	5.0		
**Commercial Claims database, United States**[109]	2007-11	America	87461	11190358	7.8		
**Medicaid database, United States**[109]	2007-11	America	79250	5716641	13.9		
**Bangalore, India**[110]	2009-11	South-East Asia	883	224966	3.9		
**Northwestern Ontario, Canada**[111]	2007-09	America	102		51.0		
**Metro Manila, Philippines**[112]	2007-09	Western Pacific	4699	158426	29.7		

Supplementary table 12: Hospitalisation rate (per 1000 children younger than 5 year per year) for pneumonia in young children (2000-2015) by World Bank income regions

Region	2000	2005	2010	2015
**Middle income**	11.5 (7.1-18.6)	15.7 (9.7-25.4)	21.4 (13.2-34.7)	29.3 (18.1-47.5)
**Low income**	5.0 (2.2-11.3)	8.1 (3.5-18.3)	13.0 (5.7-29.5)	21.0 (9.2-47.7)

Supplementary table 13: Hospitalisation rate (per 1000 children younger than 5 year per year) and estimated hospitalisations for pneumonia in young children (2000-2015) by World Bank income regions

Region	Hospitalisation rate (2000)	Hospitalisations (2000) in millions	Hospitalisation rate (2015)	Hospitalisations (2015) in millions
**Low income**	5.0 (2.2-11.4)	0.3 (0.2-0.8)	20.9 (9.1-47.9)	2.1 (0.9-4.8)
**Middle income**	11.5 (7.1-18.5)	5.4 (3.3-8.7)	29.3 (18.1-47.4)	14.3 (8.8-23.2)

Supplementary table 14: List of studies reporting in hospital case fatality ratio for children admitted with pneumonia

Study	Median study year	WHO region	World Bank Income Region	CFR (95%CI)
Goiânia, Brazil[47]	2008	Americas	Upper middle	0.3 (0.2 ,0.5)
Upper River Division and Central River Division, The Gambia[47]	2003	Africa	Low income	12 (7.4 ,19.3)
Multihospital surveillance, Bangladesh[47]	2006	South East Asia	Low income	8 (7.3 ,8.7)
Multicentric, El Salvador[47]	2007	Americas	Lower middle income	1.1 (1 ,1.2)
NhaTrang district, Vietnam (Anh et al., 2009)[104]	2006	Western Pacific	Lower middle income	2.6 (1.7 ,4.1)
Kilifi District, Kenya[47]	2005	Africa	Low income	9.5 (8.7 ,10.4)
Bondo district, Kenya[51]	2002	Africa	Low income	11 (9.2 ,13.2)
Paysandú and Salto, Uruguay[47]	2002	Americas	High income	0.3 (0.2 ,0.7)
Pilar, Argentina[47]	2004	Americas	Lower middle income	0.2 (0.1 ,0.9)
Santa Rosa, Guatemala[47]	2008	Americas	Guatemala	4.8 (2.5 ,9.3)
Goiânia, Brazil[47]	2007	Americas	Lower middle income	1.1 (0.3 ,3.3)
Nakhon Phanom and Sa Kaeo provinces, Thailand[47]	2007	South East Asia	Upper middle	0.8 (0.7 ,1)
Bangalore, India[82]	2006	South East Asia	Lower middle income	5.8 (4.5 ,7.5)
Patan, Nepal[81]	2006	South East Asia	Low income	2.2 (1.1 ,4.4)
Kamalapur, Bangladesh[47]	2008	South East Asia	Low income	0.9 (0.1 ,6.5)
Bohol, Philppines[47]	2002	Western Pacific	Lower middle income	1.6 (0.9 ,2.9)
Suva, Fiji[103]	2002	Western Pacific	Upper middle	2.8 (1.4 ,5.9)
Paysandú and Salto, Uruguay[47]	2009	Americas	High income	5.1 (3.8 ,6.8)
Nakhon Phanom and Sa Kaeo provinces, Thailand[47]	2009	South East Asia	Upper middle	0.4 (0.2 ,0.6)
Kamalapur, Bangladesh[47]	2009	South East Asia	Low income	0.7 (0.1 ,4.7)

Study	Median study year	WHO region	World Bank Income Region	CFR (95%CI)
Soweto, South Africa[47]	2010	Africa	Upper middle	0.8 (0.5 ,1.4)
Manhiça district, Mozambique[47]	2005	Africa	Low income	8.8 (6.6 ,11.7)
Multicentric, India[47]	2006	South East Asia	Lower middle income	0.3 (0.1 ,1.2)
Bondo district, Kenya[47]	2008	Africa	Low income	4.3 (3.2 ,5.8)
The Greater Banjul area and Upper River region, The Gambia[47]	2009	Africa	Low income	3.7 (2.6 ,5.2)
Lwak, Kisumu, Kenya[47]	2009	Africa	Low income	1.6 (0.7 ,3.2)
Kibera, Nairobi, Kenya[47]	2009	Africa	Low income	1.4 (0.5 ,3.7)
Karachi, Pakistan[61]	2008	Eastern Mediterranean	Lower middle income	3.7 (0.9 ,14.8)
Lwak, Kisumu, Kenya[47]	2010	Africa	Low income	1 (0.3 ,3.1)
Kibera, Nairobi, Kenya[47]	2010	Africa	Low income	0.6 (0.2 ,1.9)
Santa Rosa, Guatemala[47]	2010	Americas	Lower middle income	5 (3.6 ,7)
Quetzaltenango, Guatemala[47]	2010	Americas	Lower middle income	3.5 (2.2 ,5.5)
Soweto, South Africa[47]	2002	Africa	Upper middle	6.3 (5.4 ,7.3)
Concordia and Parana, Argentina[47]	2004	Americas	Lower middle income	1.1 (0.7 ,1.7)
Mirzapur, Bangladesh[47]	2006	South East Asia	Low income	2.2 (1.4 ,3.5)
Lombok, Indonesia[47]	2001	South East Asia	Lower middle income	11 (10.2 ,11.9)
Multicentre, Kenya[113]	2008	Africa	Low income	5.9 (5.1 ,6.8)
Western Australia[114]	2001	Western Pacific	High income	0.9 (0.7 ,1.2)
Guriel district, Somalia[115]	2010	Africa	Low income	2.1 (1.6 ,2.7)
Chennai, Indias[116]	2007	South East Asia	Lower middle income	8.2 (7.4 ,9.1)
Blantyre, Malawi[117]	2006	Africa	Low income	12.2 (8.2 ,18.1)
Kilifi, Kenya[118]	2007	Africa	Low income	6 (4.3 ,8.4)
Biliran, The Philippines[106]	2011	Western Pacific	Lower middle income	1.4 (0.4 ,5.8)

Study	Median study year	WHO region	World Bank Income Region	CFR (95%CI)
**Bangalore, India**[110]	2010	South East Asia	Lower middle income	0.8 (0.6 ,1.1)
**Multicentre, Malawi**[119]	2001	Africa	Low income	15.2 (13.3 ,17.3)
**Multicentre, Malawi**[119]	2002	Africa	Low income	11.3 (10.2 ,12.6)
**Multicentre, Malawi**[119]	2003	Africa	Low income	9.1 (8.2 ,10)
**Multicentre, Malawi**[119]	2004	Africa	Low income	9.2 (8.3 ,10.2)
**Multicentre, Malawi**[119]	2005	Africa	Low income	9.7 (9 ,10.5)
**Multicentre, Malawi**[119]	2006	Africa	Low income	7.4 (6.8 ,8)
**Multicentre, Malawi**[119]	2007	Africa	Low income	6.8 (6.4 ,7.4)
**Multicentre, Malawi**[119]	2008	Africa	Low income	7.3 (6.8 ,7.9)
**Multicentre, Malawi**[119]	2009	Africa	Low income	6.1 (5.7 ,6.5)
**Multicentre, Malawi**[119]	2010	Africa	Low income	5.3 (4.9 ,5.6)
**Multicentre, Malawi**[119]	2011	Africa	Low income	4.1 (3.8 ,4.4)
**Multicentre, Malawi**[119]	2012	Africa	Low income	4.5 (4.2 ,4.9)
**Colorado, USA**[47]	2004	Americas	High income	0.6 (0.5 ,0.7)
**USA**[54]	2000	Americas	High income	0.2 (0.2 ,0.2)
**Colorado, USA**[47]	2009	Americas	High income	0.8 (0.6 ,1.1)
**Tuscany, Italy**[120]	2005	Europe	High income	0.1 (0 ,0.2)
**Poznań and Poznański, Poland**[121]	2008	Europe	High income	0.1 (0 ,0.6)
**Malawi**[122]	2002	Africa	Low income	10.4 (9.9 ,10.9)
**Mozambique**[123]	2007	Africa	Low income	9.8 (7.9 ,12.2)
**Bangladesh**[124]	2012	South-east Asia	Lower middle income	0 (0 ,0.2)

Supplementary table 15: Number of children aged below 5 years residing in low and middle income countries and exposed to the risk factors for pneumonia in 2000 and 2015

Risk factor	year	Number of children exposed (uncertainty range)	Percentage of children <5 exposed (UR)
HIV	2000	1517247 (1413328-1652981)	0.29 (0.27-0.32)
HIV	2015	1046529 (979467-1116290)	0.18 (0.17-0.19)
Incomplete immunisation	2000	177383184 (175158137-179486533)	34.14 (33.71-34.54)
Incomplete immunisation	2015	143938193 (143106886-144715979)	24.82 (24.68-24.95)
Indoor air pollution	2000	283449840 (282633011-284312655)	54.55 (54.39-54.71)
Indoor air pollution	2015	317162319 (316345916-317999622)	54.69 (54.55-54.83)
Low birth weight	2000	45058267 (43611587-46448152)	8.67 (8.39-8.94)
Low birth weight	2015	56102037 (55579933-56663706)	9.67 (9.58-9.77)
Malnutrition	2000	169994730 (167422035-172307433)	32.71 (32.22-33.16)
Malnutrition	2015	138866321 (138055666-139679023)	23.94 (23.8-24.08)
Non-exclusive breastfeeding	2000	111180903 (110285434-112158640)	21.4 (21.22-21.58)
Non-exclusive breastfeeding	2015	145729789 (144969738-146520225)	25.13 (25-25.26)
Overcrowding	2000	274991971 (272641751-277504089)	52.92 (52.47-53.4)
Overcrowding	2015	269602621 (268770720- 270498009)	46.49 (46.34-46.64)

Supplementary table 16: Number and proportion of children aged below 5 years exposed to risk factors for pneumonia in 2000 and 2015 by WHO region

WHO Region	Risk factor	Year	Number of children <5 years exposed	Proportion of children <5 years exposed
**Africa**	HIV	2000	1359327 (1281359-1444812)	1.23 (1.16-1.31)
**Africa**	HIV	2015	935157 (876380-993682)	0.59 (0.56-0.63)
**Africa**	Incomplete immunisation	2000	50187173 (49811529-50553124)	45.51 (45.17-45.84)
**Africa**	Incomplete immunisation	2015	48954267 (48596704-49319121)	31.01 (30.78-31.24)
**Africa**	Indoor air pollution	2000	83067674 (82801791-83327464)	75.32 (75.08-75.56)
**Africa**	Indoor air pollution	2015	126385468 (126057269-126691254)	80.05 (79.85-80.25)
**Africa**	Low birth weight	2000	6677566 (6502727-6846623)	6.05 (5.9-6.21)
**Africa**	Low birth weight	2015	11740886 (11527591-11967748)	7.44 (7.3-7.58)
**Africa**	Malnutrition	2000	30637217 (30250518-30996126)	27.78 (27.43-28.11)
**Africa**	Malnutrition	2015	39398207 (39023342-39778736)	24.96 (24.72-25.2)
**Africa**	Non-exclusive breastfeeding	2000	35199474 (34854015-35561790)	31.92 (31.6-32.25)
**Africa**	Non-exclusive breastfeeding	2015	35631421 (35302371-35962243)	22.57 (22.36-22.78)
**Africa**	Overcrowding	2000	65676921 (65271690-66080104)	59.55 (59.18-59.92)
**Africa**	Overcrowding	2015	90697231 (90271048-91113680)	57.45 (57.18-57.71)
**Americas**	HIV	2000	38890 (26547-57112)	0.07 (0.05-0.1)
**Americas**	HIV	2015	15739 (11001-22377)	0.03 (0.02-0.04)
**Americas**	Incomplete immunisation	2000	14701969 (14415651-14983042)	26.06 (25.55-26.56)
**Americas**	Incomplete immunisation	2015	7839078 (7681172-8013075)	14.91 (14.61-15.24)
**Americas**	Indoor air pollution	2000	28525155 (28174495-28858713)	50.56 (49.94-51.15)
**Americas**	Indoor air pollution	2015	20430282 (20221678-20651161)	38.87 (38.47-39.29)
**Americas**	Low birth weight	2000	4979795 (4778269-5180071)	8.83 (8.47-9.18)
**Americas**	Low birth weight	2015	4837143 (4703597-4981895)	9.2 (8.95-9.48)
**Americas**	Malnutrition	2000	4396997 (4224571-4574243)	7.79 (7.49-8.11)
**Americas**	Malnutrition	2015	4167716 (4036279-4302185)	7.93 (7.68-8.18)
**Americas**	Non-exclusive breastfeeding	2000	19656560 (19341361-19974090)	34.84 (34.28-35.41)
**Americas**	Non-exclusive breastfeeding	2015	9580729 (9405765-9754210)	18.23 (17.89-18.56)

**Americas**	Overcrowding	2000	25569037 (25246038-25902275)	45.32 (44.75-45.91)
**Americas**	Overcrowding	2015	23529458 (23292239-23750698)	44.76 (44.31-45.18)
**Eastern Mediterranean**	HIV	2000	9669 (5093-17317)	0.02 (0.01-0.03)
**Eastern Mediterranean**	HIV	2015	13007 (7673-21274)	0.02 (0.01-0.03)
**Eastern Mediterranean**	Incomplete immunisation	2000	13764639 (13516337-14022351)	21.42 (21.03-21.82)
**Eastern Mediterranean**	Incomplete immunisation	2015	18091538 (17822412-18357222)	22.74 (22.4-23.07)
**Eastern Mediterranean**	Indoor air pollution	2000	33190463 (32935988-33426011)	51.65 (51.26-52.02)
**Eastern Mediterranean**	Indoor air pollution	2015	27006480 (26748652-27264002)	33.94 (33.61-34.26)
**Eastern Mediterranean**	Low birth weight	2000	5671189 (5516894-5818944)	8.83 (8.59-9.06)
**Eastern Mediterranean**	Low birth weight	2015	8694050 (8533716-8856996)	10.93 (10.72-11.13)
**Eastern Mediterranean**	Malnutrition	2000	7715006 (7546633-7889825)	12.01 (11.74-12.28)
**Eastern Mediterranean**	Malnutrition	2015	15451407 (15197935-15747717)	19.42 (19.1-19.79)
**Eastern Mediterranean**	Non-exclusive breastfeeding	2000	17738982 (17524436-17964649)	27.61 (27.27-27.96)
**Eastern Mediterranean**	Non-exclusive breastfeeding	2015	20599906 (20361046-20857997)	25.89 (25.59-26.21)
**Eastern Mediterranean**	Overcrowding	2000	41157777 (40903211-41408354)	64.05 (63.66-64.44)
**Eastern Mediterranean**	Overcrowding	2015	49120476 (48835717-49408113)	61.73 (61.37-62.09)
**South East Asia**	HIV	2000	68003 (41648-103765)	0.04 (0.02-0.06)
**South East Asia**	HIV	2015	70950 (48889-98802)	0.04 (0.03-0.06)
**South East Asia**	Incomplete immunisation	2000	67512686 (66790318-68259841)	38 (37.6-38.42)
**South East Asia**	Incomplete immunisation	2015	60435075 (59844539-61060421)	33.81 (33.47-34.15)
**South East Asia**	Indoor air pollution	2000	123930679 (123232981-124588730)	69.76 (69.37-70.13)
**South East Asia**	Indoor air pollution	2015	124716236 (124112463-125342264)	69.76 (69.42-70.11)
**South East Asia**	Low birth weight	2000	17883921 (17443377-18384735)	10.07 (9.82-10.35)
**South East Asia**	Low birth weight	2015	25453233 (25007115-25920165)	14.24 (13.99-14.5)
**South East Asia**	Malnutrition	2000	75367309 (74615634-76097785)	42.42 (42-42.83)
**South East Asia**	Malnutrition	2015	71476697 (70837057-72085927)	39.98 (39.62-40.32)

**South East Asia**	Non-exclusive breastfeeding	2000	29238112 (28846281-29688741)	16.46 (16.24-16.71)
**South East Asia**	Non-exclusive breastfeeding	2015	38796183 (38292551-39275361)	21.7 (21.42-21.97)
**South East Asia**	Overcrowding	2000	92554939 (91810611-93345362)	52.1 (51.68-52.54)
**South East Asia**	Overcrowding	2015	94744206 (94108902-95400693)	53 (52.64-53.36)
**Western Pacific**	HIV	2000	41359 (6665-157357)	0.04 (0.01-0.14)
**Western Pacific**	HIV	2015	11675 (5997-20460)	0.01 (0.01-0.02)
**Western Pacific**	Incomplete immunisation	2000	31216717 (29217765-33111860)	28.12 (26.32-29.82)
**Western Pacific**	Incomplete immunisation	2015	8618235 (8405198-8835134)	7.75 (7.56-7.95)
**Western Pacific**	Indoor air pollution	2000	14735869 (14648160-14876786)	13.27 (13.19-13.4)
**Western Pacific**	Indoor air pollution	2015	18623854 (18388328-18851831)	16.75 (16.54-16.96)
**Western Pacific**	Low birth weight	2000	9845797 (8567230-11093255)	8.87 (7.72-9.99)
**Western Pacific**	Low birth weight	2015	5376725 (5212534-5548181)	4.84 (4.69-4.99)
**Western Pacific**	Malnutrition	2000	51878201 (49559122-54047723)	46.73 (44.64-48.68)
**Western Pacific**	Malnutrition	2015	8372293 (8182197-8562801)	7.53 (7.36-7.7)
**Western Pacific**	Non-exclusive breastfeeding	2000	9347775 (8740731-10055203)	8.42 (7.87-9.06)
**Western Pacific**	Non-exclusive breastfeeding	2015	41121550 (40721572-41536390)	36.99 (36.63-37.36)
**Western Pacific**	Overcrowding	2000	50033298 (47790734-52380411)	45.07 (43.05-47.18)
**Western Pacific**	Overcrowding	2015	11511251 (11279656-11728475)	10.35 (10.15-10.55)

Additional care is required in interpreting the results for the Western Pacific region as there was a considerable increase in Countries contributing data to the DHS between 2000 and 2015. Only 2 countries (Cambodia and the Philippines) of the 20 of countries contributing data in 2015 contributed in 2000. This means that the regional comparison was particularly vulnerable to the imputation approached described in the analysis. This was not an issue in the remaining regions where the majority of countries contributing to the DHS survey in the 2015 period also contributed in 2000; the percentages were 86%, 90%, 100% and 100% for the Africa, Americas, Eastern Mediterranean and South-East Asian regions respectively.

Supplementary table 17: Pneumonia deaths among children under-five for 132 low and middle income countries, 2000-2015

Country	Pneumonia deaths among children under-five				Difference between 2000 and 2015	
	2000	2005	2010	2015	Absolute difference	Relative difference
Afghanistan	28942	29981	25105	18671	-10271	-35%
Algeria	3723	3303	3117	3130	-592	-16%
Angola	30484	36275	34571	29502	-982	-3%
Antigua and Barbuda	0	1	0	0	0	0%
Argentina	968	886	823	708	-260	-27%
Bahamas	19	9	15	9	-10	-52%
Bahrain	3	6	7	3	0	11%
Bangladesh	62632	45036	25034	17352	-45281	-72%
Barbados	0	3	1	1	1	0%
Belize	24	14	13	11	-13	-53%
Benin	7229	6413	6555	5702	-1528	-21%
Bhutan	277	172	114	60	-217	-78%
Bolivia (Plurinational State of)	3728	2594	1903	1304	-2424	-65%
Botswana	330	391	429	307	-22	-7%
Brazil	14929	7549	3415	4773	-10156	-68%
Burkina Faso	9774	11872	7856	7761	-2013	-21%
Burundi	7522	7086	7322	5913	-1609	-21%
Cabo Verde	76	49	49	37	-39	-52%
Cambodia	7405	4632	2739	1694	-5712	-77%
Cameroon	16115	14747	14227	11071	-5045	-31%
Central African Republic	3135	2978	3420	3368	233	7%
Chad	15765	18810	19393	19275	3511	22%
Chile	276	142	89	67	-209	-76%
China	118092	60173	33332	22242	-95850	-81%
Colombia	2631	1973	1372	1217	-1415	-54%
Comoros	373	403	330	300	-72	-19%
Congo	2202	2101	1407	971	-1232	-56%

Country	Pneumonia deaths among children under-five				Difference between 2000 and 2015	
	2000	2005	2010	2015	Absolute difference	Relative difference
Cook Islands	1	1	0	0	-1	-81%
Costa Rica	74	51	34	33	-41	-56%
Cote d'Ivoire	9279	8641	9323	10935	1656	18%
Democratic People's Republic of Korea	4637	2146	1854	1383	-3254	-70%
Democratic Republic of the Congo	41587	38820	44934	46226	4639	11%
Djibouti	367	325	235	184	-183	-50%
Dominica	0	1	0	1	0	84%
Dominican Republic	1164	884	782	700	-464	-40%
Ecuador	1573	1153	1059	861	-712	-45%
Egypt	15545	10894	8216	8375	-7170	-46%
El Salvador	835	456	301	215	-620	-74%
Equatorial Guinea	485	381	463	418	-67	-14%
Eritrea	2402	2388	1824	1551	-851	-35%
Ethiopia	86397	68880	44478	31427	-54971	-64%
Fiji	53	51	57	47	-6	-10%
Gabon	397	445	396	355	-42	-11%
Gambia	998	977	836	766	-232	-23%
Ghana	7923	8013	7740	6450	-1472	-19%
Grenada	3	2	1	2	-2	-52%
Guatemala	4456	3190	2566	2146	-2309	-52%
Guinea	8732	8403	7050	7008	-1724	-20%
Guinea-Bissau	1523	1591	1178	1028	-495	-33%
Guyana	74	52	31	44	-30	-40%
Haiti	6537	5412	6037	4021	-2516	-38%
Honduras	1156	753	539	372	-784	-68%
India	481539	371044	270948	178994	-302545	-63%
Indonesia	44917	34851	27372	25000	-19918	-44%
Iran (Islamic Republic of)	7320	4644	3877	3098	-4222	-58%

Country	Pneumonia deaths among children under-five				Difference between 2000 and 2015	
	2000	2005	2010	2015	Absolute difference	Relative difference
Iraq	6354	6191	6648	5874	-480	-8%
Jamaica	68	56	56	41	-27	-40%
Jordan	481	359	347	327	-155	-32%
Kenya	21276	17415	14681	10507	-10769	-51%
Kiribati	37	27	38	27	-10	-26%
Kuwait	35	23	41	56	21	60%
Lao People's Democratic Republic	4314	3748	2989	2006	-2308	-54%
Lebanon	132	75	33	46	-85	-65%
Lesotho	1086	1078	942	955	-130	-12%
Liberia	2845	2492	2242	1737	-1108	-39%
Libya	369	278	187	127	-242	-66%
Madagascar	15968	12599	9220	6951	-9017	-56%
Malawi	13366	7975	7031	5416	-7950	-59%
Malaysia	484	271	204	185	-299	-62%
Maldives	45	18	9	5	-41	-89%
Mali	17781	16339	15731	10766	-7015	-39%
Marshall Islands	15	13	10	8	-7	-47%
Mauritania	2162	2438	2169	1616	-546	-25%
Mauritius	13	21	21	21	8	65%
Mexico	10458	6895	4877	3459	-6999	-67%
Micronesia (Federated States of)	29	22	15	12	-17	-58%
Mongolia	594	361	288	236	-358	-60%
Morocco	5299	3834	2864	2246	-3052	-58%
Mozambique	17254	17590	14809	11757	-5497	-32%
Myanmar	17745	15336	11019	7516	-10230	-58%
Namibia	659	661	596	618	-41	-6%
Nauru	1	1	1	1	0	-39%
Nepal	12038	8004	5208	2946	-9092	-76%

Country	Pneumonia deaths among children under-five				Difference between 2000 and 2015	
	2000	2005	2010	2015	Absolute difference	Relative difference
Nicaragua	993	693	574	431	-563	-57%
Niger	25830	25072	19325	18290	-7541	-29%
Nigeria	145231	149071	157059	133239	-11992	-8%
Niue	0	0	0	0	0	-24%
Oman	102	57	55	60	-41	-41%
Pakistan	93923	84850	78094	63960	-29963	-32%
Palau	1	1	0	0	0	-44%
Panama	185	194	228	170	-15	-8%
Papua New Guinea	2300	2516	2264	1934	-366	-16%
Paraguay	705	488	401	305	-400	-57%
Peru	3952	2199	1487	1076	-2876	-73%
Philippines	19200	17475	15141	12224	-6976	-36%
Qatar	8	6	7	9	1	17%
Republic of Korea	188	77	59	39	-149	-79%
Rwanda	12710	6657	3573	1929	-10781	-85%
Saint Kitts and Nevis	1	0	0	0	-1	-100%
Saint Lucia	2	3	3	2	0	1%
Saint Vincent and the Grenadines	2	1	2	2	0	1%
Samoa	15	11	10	8	-7	-47%
Sao Tome and Principe	72	59	44	35	-37	-52%
Saudi Arabia	1284	857	675	491	-793	-62%
Senegal	9729	8264	5567	3984	-5745	-59%
Seychelles	1	1	1	1	0	-9%
Sierra Leone	5914	6766	4864	3714	-2199	-37%
Solomon Islands	75	86	95	85	10	14%
Somalia	12480	14883	17018	14669	2189	18%
South Africa	12851	10431	10521	7105	-5746	-45%
Sri Lanka	550	426	264	193	-357	-65%

Country	Pneumonia deaths among children under-five				Difference between 2000 and 2015	
	2000	2005	2010	2015	Absolute difference	Relative difference
Sudan	24491	24467	20654	15551	-8940	-37%
Suriname	31	24	22	16	-15	-47%
Swaziland	696	721	517	354	-342	-49%
Syrian Arab Republic	1749	1017	806	427	-1322	-76%
Thailand	2651	1725	1149	843	-1808	-68%
Timor-Leste	972	584	460	550	-422	-43%
Togo	2831	2857	2716	2962	131	5%
Tonga	6	5	5	5	-1	-18%
Trinidad and Tobago	17	43	36	30	13	76%
Tunisia	822	451	331	234	-589	-72%
Tuvalu	2	1	1	1	-1	-66%
Uganda	22161	19172	16025	13659	-8502	-38%
United Arab Emirates	31	27	33	25	-5	-17%
United Republic of Tanzania	31096	27617	19171	14322	-16773	-54%
Uruguay	79	73	53	31	-48	-61%
Vanuatu	26	25	32	26	0	-1%
Venezuela (Bolivarian Republic of)	1014	1051	1047	965	-49	-5%
Viet Nam	8603	7754	5685	4808	-3795	-44%
Yemen	14202	10937	8027	5373	-8830	-62%
Zambia	12365	8815	8202	6020	-6345	-51%
Zimbabwe	5108	6570	6847	5582	474	9%

Supplementary table 18: Pneumonia mortality among children under-five for 132 low and middle income countries, 2000-2015

Country	Pneumonia mortality among children under-five				Difference between 2000 and 2015		
	2000	2005	2010	2015	Absolute difference	Relative difference	Annual Rate of Change*
Afghanistan	30.8	28.4	23.0	18.3	-12.5	-41%	3.5
Algeria	6.6	5.0	3.4	3.3	-3.3	-50%	4.6
Angola	41.3	41.9	34.9	27.0	-14.3	-35%	2.8
Antigua and Barbuda	0.0	0.6	0.3	0.3	0.3	0%	0.0
Argentina	1.4	1.2	1.1	0.9	-0.5	-34%	2.7
Bahamas	3.7	1.6	2.3	1.6	-2.1	-57%	5.7
Bahrain	0.2	0.4	0.4	0.1	-0.1	-35%	2.9
Bangladesh	17.3	12.9	7.9	5.4	-11.8	-69%	7.7
Barbados	0.0	0.8	0.4	0.3	0.3	0%	0.0
Belize	3.1	1.9	1.7	1.4	-1.8	-57%	5.6
Benin	25.3	19.9	18.7	15.2	-10.1	-40%	3.4
Bhutan	17.5	11.7	7.5	4.9	-12.6	-72%	8.5
Bolivia (Plurinational State of)	14.7	10.4	7.6	5.3	-9.4	-64%	6.8
Botswana	7.0	8.4	8.3	5.3	-1.7	-24%	1.9
Brazil	3.9	2.2	1.2	1.5	-2.4	-62%	6.5
Burkina Faso	19.0	20.5	12.2	11.3	-7.7	-41%	3.5
Burundi	27.2	21.8	17.9	12.8	-14.4	-53%	5.0
Cabo Verde	6.0	4.4	4.3	3.4	-2.7	-44%	3.9
Cambodia	23.8	13.6	7.2	4.7	-19.1	-80%	10.8
Cameroon	25.6	20.7	18.6	13.5	-12.1	-47%	4.3
Central African Republic	21.7	20.5	23.2	20.5	-1.1	-5%	0.4
Chad	38.9	39.5	35.9	31.8	-7.1	-18%	1.3

* For formula see p2155 of the - Li Liu, Hope L. Johnson, Simon Cousens, Jamie Perin, Susana Scott, Joy Lawn, Igor Rudan, Harry Campbell, Richard Cibulskis, Mengying Li, Colin Mathers, and Robert E. Black, for the Child Health Epidemiology Reference Group (CHERG) of WHO and UNICEF. 2012. “Global, regional, and national causes of child mortality: an updated systematic analysis for 2010 with time trends since 2000”. Lancet. 379: 2151-61.

Country	Pneumonia mortality among children under-five				Difference between 2000 and 2015		
	2000	2005	2010	2015	Absolute difference	Relative difference	Annual Rate of Change*
**Chile**	1.1	0.6	0.4	0.3	-0.8	-73%	8.8
**China**	7.6	3.5	2.1	1.3	-6.2	-83%	11.7
**Colombia**	3.2	2.3	1.8	1.6	-1.6	-49%	4.5
**Comoros**	18.3	18.3	13.6	11.5	-6.9	-37%	3.1
**Congo**	18.8	16.1	9.4	5.9	-12.9	-68%	7.7
**Cook Islands**	1.8	1.8	0.8	0.4	-1.4	-78%	10.1
**Costa Rica**	0.9	0.7	0.5	0.5	-0.5	-49%	4.5
**Cote d'Ivoire**	14.0	12.6	12.6	13.2	-0.8	-6%	0.4
**Democratic People's Republic of Korea**	11.9	5.6	5.6	3.7	-8.3	-69%	7.9
**Democratic Republic of the Congo**	19.3	15.5	16.0	14.8	-4.6	-24%	1.8
**Djibouti**	16.8	14.5	11.1	8.3	-8.5	-50%	4.7
**Dominica**	0.5	0.8	0.1	0.8	0.4	76%	-3.8
**Dominican Republic**	5.6	3.9	3.6	3.2	-2.4	-43%	3.7
**Ecuador**	5.0	3.7	3.2	2.6	-2.4	-47%	4.3
**Egypt**	9.1	5.6	4.1	3.0	-6.1	-67%	7.4
**El Salvador**	6.0	3.9	2.6	2.1	-3.9	-66%	7.1
**Equatorial Guinea**	23.6	16.9	17.7	14.7	-9.0	-38%	3.2
**Eritrea**	19.4	15.1	10.8	9.3	-10.0	-52%	4.9
**Ethiopia**	29.6	23.6	15.0	10.0	-19.6	-66%	7.3
**Fiji**	2.7	2.6	2.9	2.8	0.1	2%	-0.1
**Gabon**	9.9	10.6	8.3	6.9	-3.0	-30%	2.4
**Gambia**	18.4	15.7	11.7	9.3	-9.1	-50%	4.6
**Ghana**	12.7	11.1	9.8	7.2	-5.5	-43%	3.8
**Grenada**	1.5	1.3	0.4	0.8	-0.7	-46%	4.1
**Guatemala**	11.2	7.8	6.3	4.8	-6.4	-57%	5.6
**Guinea**	24.2	22.1	17.1	15.4	-8.9	-37%	3.0

Country	Pneumonia mortality among children under-five				Difference between 2000 and 2015		
	2000	2005	2010	2015	Absolute difference	Relative difference	Annual Rate of Change*
Guinea-Bissau	29.8	28.8	19.7	15.8	-14.0	-47%	4.2
Guyana	4.0	3.4	2.8	2.6	-1.4	-35%	2.9
Haiti	24.6	20.8	22.1	15.6	-8.9	-36%	3.0
Honduras	5.9	4.1	3.1	2.3	-3.6	-61%	6.3
India	17.3	13.5	10.2	7.1	-10.2	-59%	5.9
Indonesia	10.3	7.8	5.9	4.6	-5.8	-56%	5.4
Iran (Islamic Republic of)	5.7	3.8	2.8	2.3	-3.4	-60%	6.1
Iraq	7.5	6.9	6.0	4.8	-2.7	-36%	3.0
Jamaica	1.2	1.2	1.2	1.0	-0.2	-13%	0.9
Jordan	3.2	2.4	1.9	1.6	-1.6	-51%	4.7
Kenya	18.2	13.0	10.2	6.9	-11.3	-62%	6.5
Kiribati	14.2	15.3	10.4	9.5	-4.7	-33%	2.7
Kuwait	0.8	0.5	0.6	0.8	0.0	6%	-0.4
Lao People's Democratic Republic	26.8	23.0	16.2	11.6	-15.2	-57%	5.6
Lebanon	2.2	1.2	0.7	0.5	-1.7	-79%	10.4
Lesotho	18.4	20.9	16.6	15.3	-3.1	-17%	1.2
Liberia	23.5	19.5	15.7	11.5	-12.0	-51%	4.8
Libya	3.1	2.2	1.3	1.0	-2.1	-67%	7.4
Madagascar	24.8	18.9	12.6	8.5	-16.3	-66%	7.2
Malawi	28.2	15.6	11.7	8.6	-19.6	-69%	7.9
Malaysia	0.9	0.6	0.5	0.3	-0.5	-62%	6.4
Maldives	7.3	2.8	1.2	0.6	-6.7	-92%	16.5
Mali	35.1	27.7	23.1	14.8	-20.2	-58%	5.7
Marshall Islands	6.9	6.8	6.3	6.0	-0.8	-12%	0.9
Mauritania	21.4	21.5	17.6	12.3	-9.1	-43%	3.7
Mauritius	0.7	1.0	1.5	1.5	0.7	96%	-4.5

Country	Pneumonia mortality among children under-five				Difference between 2000 and 2015		
	2000	2005	2010	2015	Absolute difference	Relative difference	Annual Rate of Change*
Mexico	4.2	2.9	2.1	1.5	-2.7	-65%	7.0
Micronesia (Federated States of)	9.0	8.1	6.0	5.1	-3.9	-44%	3.8
Mongolia	12.4	7.5	4.3	3.3	-9.1	-73%	8.8
Morocco	8.7	6.3	4.1	3.1	-5.6	-64%	6.8
Mozambique	21.7	19.8	15.2	11.1	-10.6	-49%	4.5
Myanmar	15.3	13.2	11.2	8.2	-7.1	-47%	4.2
Namibia	11.6	11.3	9.3	8.3	-3.3	-28%	2.2
Nauru	6.8	6.5	4.9	4.7	-2.0	-30%	2.4
Nepal	15.7	11.7	8.2	5.3	-10.3	-66%	7.2
Nicaragua	7.3	5.4	4.4	3.6	-3.7	-51%	4.7
Niger	45.1	37.6	24.4	19.2	-25.9	-57%	5.7
Nigeria	28.8	26.1	24.9	19.0	-9.7	-34%	2.8
Niue	6.5	0.9	6.9	5.5	-1.1	-17%	1.2
Oman	1.9	1.1	0.8	0.7	-1.1	-60%	6.1
Pakistan	21.0	19.7	14.8	11.9	-9.1	-43%	3.8
Palau	2.6	2.1	2.1	1.2	-1.4	-53%	5.0
Panama	2.5	2.9	3.0	2.3	-0.3	-11%	0.8
Papua New Guinea	12.3	13.2	10.9	9.2	-3.1	-25%	1.9
Paraguay	4.8	3.8	2.8	2.3	-2.5	-53%	5.0
Peru	6.5	3.6	2.5	1.7	-4.7	-73%	8.8
Philippines	8.4	7.3	6.7	5.2	-3.2	-38%	3.2
Qatar	0.6	0.5	0.4	0.3	-0.3	-51%	4.7
Republic of Korea	0.4	0.2	0.1	0.1	-0.3	-77%	9.7
Rwanda	36.0	20.7	9.6	5.7	-30.3	-84%	12.3
Saint Kitts and Nevis	0.5	0.3	0.2	0.0	-0.5	-100%	0.0
Saint Lucia	0.7	1.1	1.1	0.8	0.1	9%	-0.6

Country	Pneumonia mortality among children under-five				Difference between 2000 and 2015		
	2000	2005	2010	2015	Absolute difference	Relative difference	Annual Rate of Change*
Saint Vincent and the Grenadines	0.7	0.5	0.8	1.0	0.2	33%	-1.9
Samoa	2.9	2.0	1.8	1.7	-1.2	-40%	3.4
Sao Tome and Principe	13.7	10.4	7.6	5.5	-8.2	-60%	6.1
Saudi Arabia	2.2	1.5	1.1	0.8	-1.5	-65%	6.9
Senegal	25.5	19.0	11.4	6.9	-18.6	-73%	8.7
Seychelles	0.9	0.8	0.6	0.7	-0.2	-23%	1.7
Sierra Leone	34.1	32.7	22.0	17.0	-17.0	-50%	4.6
Solomon Islands	5.4	5.5	5.6	5.1	-0.3	-6%	0.4
Somalia	34.9	39.9	39.4	32.9	-2.0	-6%	0.4
South Africa	11.0	10.1	8.5	7.0	-4.0	-36%	3.0
Sri Lanka	1.6	1.1	0.7	0.6	-1.0	-62%	6.5
Sudan	22.7	20.5	16.9	12.0	-10.7	-47%	4.2
Suriname	2.8	2.4	2.2	1.6	-1.2	-42%	3.6
Swaziland	21.0	21.2	14.2	9.7	-11.3	-54%	5.2
Syrian Arab Republic	3.3	2.2	1.5	1.0	-2.3	-70%	8.0
Thailand	3.0	2.0	1.5	1.1	-1.8	-61%	6.4
Timor-Leste	24.6	18.4	15.3	10.6	-14.0	-57%	5.6
Togo	15.2	13.3	11.5	11.8	-3.4	-22%	1.7
Tonga	2.3	1.8	1.9	1.8	-0.4	-18%	1.3
Trinidad and Tobago	0.9	2.1	1.8	1.6	0.7	72%	-3.6
Tunisia	4.9	2.7	1.8	1.1	-3.8	-77%	9.8
Tuvalu	7.6	5.9	3.9	3.5	-4.1	-54%	5.2
Uganda	20.3	15.0	11.2	8.6	-11.7	-58%	5.7
United Arab Emirates	0.6	0.4	0.4	0.3	-0.3	-57%	5.6
United Republic of Tanzania	23.2	17.1	10.7	7.0	-16.2	-70%	8.0
Uruguay	1.5	1.4	1.1	0.6	-0.9	-57%	5.7

Country	Pneumonia mortality among children under-five				Difference between 2000 and 2015		
	2000	2005	2010	2015	Absolute difference	Relative difference	Annual Rate of Change*
**Vanuatu**	4.4	4.1	4.1	3.9	-0.5	-12%	0.9
**Venezuela (Bolivarian Republic of)**	1.7	1.8	1.8	1.6	-0.1	-8%	0.6
**Viet Nam**	7.3	5.1	3.7	3.1	-4.2	-58%	5.8
**Yemen**	22.0	14.5	10.0	6.5	-15.5	-70%	8.1
**Zambia**	27.6	17.4	14.4	9.8	-17.8	-65%	6.9
**Zimbabwe**	13.0	15.2	13.7	10.2	-2.8	-21%	1.6

Supplementary table 19: Pneumonia deaths among children under-five for all countries and selected regions, 2000-2015

Level	Country	Pneumonia deaths among children under-five			
		2000	2005	2010	2015
Region	Low income region*	442418	397618	336014	281143
	Middle income region*	1291163	1032085	833061	636871
	Developing countries	1694792	1401246	1146196	898291
	Africa#	645206	602249	547266	463098
	Southeast Asia#	628004	479342	343430	234841
Country	Afghanistan	28942	29981	25105	18671
	Angola	30484	36275	34571	29502
	Albania	266	135	72	74
	Andorra	0	0	0	0
	United Arab Emirates	31	27	33	25
	Argentina	968	886	823	708
	Armenia	218	170	106	62
	Antigua and Barbuda	0	1	0	0
	Australia	40	42	42	31
	Austria	8	1	6	4
	Azerbaijan	1877	1182	1041	881
	Burundi	7522	7086	7322	5913
	Belgium	8	18	15	9
	Benin	7229	6413	6555	5702
	Burkina Faso	9774	11872	7856	7761
	Bangladesh	62632	45036	25034	17352
	Bulgaria	290	219	195	108
	Bahrain	3	6	7	3
	Bahamas	19	9	15	9
	Bosnia and Herzegovina	35	17	19	9
	Belarus	181	96	50	34
	Belize	24	14	13	11

Level	Country	Pneumonia deaths among children under-five			
		2000	2005	2010	2015
	Bolivia (Plurinational State of)	3728	2594	1903	1304
	Brazil	14929	7549	3415	4773
	Barbados	0	3	1	1
	Brunei Darussalam	3	3	3	3
	Bhutan	277	172	114	60
	Botswana	330	391	429	307
	Central African Republic	3135	2978	3420	3368
	Canada	52	40	43	38
	Switzerland	6	8	7	4
	Chile	276	142	89	67
	China	118092	60173	33332	22242
	Cote d'Ivoire	9279	8641	9323	10935
	Cameroon	16115	14747	14227	11071
	Democratic Republic of the Congo	41587	38820	44934	46226
	Congo	2202	2101	1407	971
	Cook Islands	1	1	0	0
	Colombia	2631	1973	1372	1217
	Comoros	373	403	330	300
	Cabo Verde	76	49	49	37
	Costa Rica	74	51	34	33
	Cuba	81	88	75	72
	Cyprus	4	2	1	1
	Czech Republic	21	24	36	20
	Germany	74	68	41	47
	Djibouti	367	325	235	184
	Dominica	0	1	0	1
	Denmark	9	7	3	2
	Dominican Republic	1164	884	782	700

Level	Country	Pneumonia deaths among children under-five			
		2000	2005	2010	2015
	Algeria	3723	3303	3117	3130
	Ecuador	1573	1153	1059	861
	Egypt	15545	10894	8216	8375
	Eritrea	2402	2388	1824	1551
	Spain	49	39	43	23
	Estonia	10	4	6	2
	Ethiopia	86397	68880	44478	31427
	Finland	4	6	6	3
	Fiji	53	51	57	47
	France	61	65	59	60
	Micronesia (Federated States of)	29	22	15	12
	Gabon	397	445	396	355
	United Kingdom	185	167	178	123
	Georgia	329	189	104	54
	Ghana	7923	8013	7740	6450
	Guinea	8732	8403	7050	7008
	Gambia	998	977	836	766
	Guinea-Bissau	1523	1591	1178	1028
	Equatorial Guinea	485	381	463	418
	Greece	24	32	17	16
	Grenada	3	2	1	2
	Guatemala	4456	3190	2566	2146
	Guyana	74	52	31	44
	Honduras	1156	753	539	372
	Croatia	24	15	8	4
	Haiti	6537	5412	6037	4021
	Hungary	70	55	25	26
	Indonesia	44917	34851	27372	25000

Level	Country	Pneumonia deaths among children under-five			
		2000	2005	2010	2015
	India	481539	371044	270948	178994
	Ireland	13	12	13	5
	Iran (Islamic Republic of)	7320	4644	3877	3098
	Iraq	6354	6191	6648	5874
	Iceland	0	0	0	0
	Israel	15	24	9	12
	Italy	53	38	27	25
	Jamaica	68	56	56	41
	Jordan	481	359	347	327
	Japan	357	279	224	185
	Kazakhstan	1687	1574	1151	726
	Kenya	21276	17415	14681	10507
	Kyrgyzstan	992	701	591	509
	Cambodia	7405	4632	2739	1694
	Kiribati	37	27	38	27
	Saint Kitts and Nevis	1	0	0	0
	Republic of Korea	188	77	59	39
	Kuwait	35	23	41	56
	Lao People's Democratic Republic	4314	3748	2989	2006
	Lebanon	132	75	33	46
	Liberia	2845	2492	2242	1737
	Libya	369	278	187	127
	Saint Lucia	2	3	3	2
	Sri Lanka	550	426	264	193
	Lesotho	1086	1078	942	955
	Lithuania	37	29	6	10
	Luxembourg	0	0	0	0
	Latvia	15	23	7	5

Level	Country	Pneumonia deaths among children under-five			
		2000	2005	2010	2015
	Morocco	5299	3834	2864	2246
	Monaco	0	0	0	0
	Republic of Moldova	393	229	145	108
	Madagascar	15968	12599	9220	6951
	Maldives	45	18	9	5
	Mexico	10458	6895	4877	3459
	Marshall Islands	15	13	10	8
	The former Yugoslav Republic of Macedonia	34	23	15	8
	Mali	17781	16339	15731	10766
	Malta	1	1	0	1
	Myanmar	17745	15336	11019	7516
	Montenegro	3	4	2	1
	Mongolia	594	361	288	236
	Mozambique	17254	17590	14809	11757
	Mauritania	2162	2438	2169	1616
	Mauritius	13	21	21	21
	Malawi	13366	7975	7031	5416
	Malaysia	484	271	204	185
	Namibia	659	661	596	618
	Niger	25830	25072	19325	18290
	Nigeria	145231	149071	157059	133239
	Nicaragua	993	693	574	431
	Niue	0	0	0	0
	Netherlands	28	25	22	13
	Norway	10	7	3	3
	Nepal	12038	8004	5208	2946
	Nauru	1	1	1	1
	New Zealand	23	25	24	22

Level	Country	Pneumonia deaths among children under-five			
		2000	2005	2010	2015
	Oman	102	57	55	60
	Pakistan	93923	84850	78094	63960
	Panama	185	194	228	170
	Peru	3952	2199	1487	1076
	Philippines	19200	17475	15141	12224
	Palau	1	1	0	0
	Papua New Guinea	2300	2516	2264	1934
	Poland	167	128	110	85
	Democratic People's Republic of Korea	4637	2146	1854	1383
	Portugal	29	19	7	6
	Paraguay	705	488	401	305
	Qatar	8	6	7	9
	Romania	2186	1190	865	526
	Russian Federation	4632	2725	1615	1290
	Rwanda	12710	6657	3573	1929
	Saudi Arabia	1284	857	675	491
	Sudan	24491	24467	20654	15551
	Senegal	9729	8264	5567	3984
	Singapore	16	26	11	20
	Solomon Islands	75	86	95	85
	Sierra Leone	5914	6766	4864	3714
	El Salvador	835	456	301	215
	San Marino	0	0	0	0
	Somalia	12480	14883	17018	14669
	Serbia	86	55	30	30
	South Sudan	9754	8600	8493	8058
	Sao Tome and Principe	72	59	44	35
	Suriname	31	24	22	16

Level	Country	Pneumonia deaths among children under-five			
		2000	2005	2010	2015
	Slovakia	68	52	34	35
	Slovenia	0	1	2	1
	Sweden	6	10	11	14
	Swaziland	696	721	517	354
	Seychelles	1	1	1	1
	Syrian Arab Republic	1749	1017	806	427
	Chad	15765	18810	19393	19275
	Togo	2831	2857	2716	2962
	Thailand	2651	1725	1149	843
	Tajikistan	4031	2508	2100	2048
	Turkmenistan	1621	1689	1138	983
	Timor-Leste	972	584	460	550
	Tonga	6	5	5	5
	Trinidad and Tobago	17	43	36	30
	Tunisia	822	451	331	234
	Turkey	4489	2440	1030	725
	Tuvalu	2	1	1	1
	United Republic of Tanzania	31096	27617	19171	14322
	Uganda	22161	19172	16025	13659
	Ukraine	1111	660	601	331
	Uruguay	79	73	53	31
	United States of America	988	1018	832	675
	Uzbekistan	6539	5293	4515	3909
	Saint Vincent and the Grenadines	2	1	2	2
	Venezuela (Bolivarian Republic of)	1014	1051	1047	965
	Viet Nam	8603	7754	5685	4808
	Vanuatu	26	25	32	26
	Samoa	15	11	10	8

Level	Country	Pneumonia deaths among children under-five			
		2000	2005	2010	2015
	Yemen	14202	10937	8027	5373
	South Africa	12851	10431	10521	7105
	Zambia	12365	8815	8202	6020
	Zimbabwe	5108	6570	6847	5582

* World Bank region, # WHO region

Supplementary table 20: Pneumonia mortality among children under-five for all countries and selected regions, 2000-2015

Level	Country	Pneumonia deaths among children under-five			
		2000	2005	2010	2015
Region	Low income region*	25.5	21.0	16.2	12.6
	Middle income region*	13.1	10.2	8.2	6.1
	Developing countries	15.2	12.1	9.7	7.4
	Africa#	24.6	20.8	16.9	13.2
	Southeast Asia#	15.9	12.3	9.1	6.4
Country	Afghanistan	30.8	28.4	23.0	18.3
	Angola	41.3	41.9	34.9	27.0
	Albania	5.1	3.4	2.2	1.6
	Andorra	0.1	0.2	0.1	0.1
	United Arab Emirates	0.6	0.4	0.4	0.3
	Argentina	1.4	1.2	1.1	0.9
	Armenia	6.2	3.7	2.3	1.5
	Antigua and Barbuda	0.0	0.6	0.3	0.3
	Australia	0.2	0.2	0.1	0.1
	Austria	0.1	0.0	0.1	0.1
	Azerbaijan	15.0	9.0	6.3	3.8
	Burundi	27.2	21.8	17.9	12.8
	Belgium	0.1	0.1	0.1	0.1
	Benin	25.3	19.9	18.7	15.2
	Burkina Faso	19.0	20.5	12.2	11.3
	Bangladesh	17.3	12.9	7.9	5.4
	Bulgaria	4.4	3.3	2.5	1.8
	Bahrain	0.2	0.4	0.4	0.1
	Bahamas	3.7	1.6	2.3	1.6
	Bosnia and Herzegovina	0.7	0.7	0.4	0.3
	Belarus	2.0	1.0	0.5	0.3
	Belize	3.1	1.9	1.7	1.4

Level	Country	Pneumonia deaths among children under-five			
		2000	2005	2010	2015
	Bolivia (Plurinational State of)	14.7	10.4	7.6	5.3
	Brazil	3.9	2.2	1.2	1.5
	Barbados	0.0	0.8	0.4	0.3
	Brunei Darussalam	0.5	0.4	0.5	0.4
	Bhutan	17.5	11.7	7.5	4.9
	Botswana	7.0	8.4	8.3	5.3
	Central African Republic	21.7	20.5	23.2	20.5
	Canada	0.2	0.1	0.1	0.1
	Switzerland	0.1	0.1	0.1	0.0
	Chile	1.1	0.6	0.4	0.3
	China	7.6	3.5	2.1	1.3
	Cote d'Ivoire	14.0	12.6	12.6	13.2
	Cameroon	25.6	20.7	18.6	13.5
	Democratic Republic of the Congo	19.3	15.5	16.0	14.8
	Congo	18.8	16.1	9.4	5.9
	Cook Islands	1.8	1.8	0.8	0.4
	Colombia	3.2	2.3	1.8	1.6
	Comoros	18.3	18.3	13.6	11.5
	Cabo Verde	6.0	4.4	4.3	3.4
	Costa Rica	0.9	0.7	0.5	0.5
	Cuba	0.5	0.7	0.6	0.6
	Cyprus	0.4	0.2	0.1	0.1
	Czech Republic	0.3	0.2	0.3	0.2
	Germany	0.1	0.1	0.1	0.1
	Djibouti	16.8	14.5	11.1	8.3
	Dominica	0.5	0.8	0.1	0.8
	Denmark	0.1	0.1	0.0	0.0
	Dominican Republic	5.6	3.9	3.6	3.2

Level	Country	Pneumonia deaths among children under-five			
		2000	2005	2010	2015
	Algeria	6.6	5.0	3.4	3.3
	Ecuador	5.0	3.7	3.2	2.6
	Egypt	9.1	5.6	4.1	3.0
	Eritrea	19.4	15.1	10.8	9.3
	Spain	0.1	0.1	0.1	0.1
	Estonia	0.8	0.3	0.3	0.2
	Ethiopia	29.6	23.6	15.0	10.0
	Finland	0.1	0.1	0.1	0.1
	Fiji	2.7	2.6	2.9	2.8
	France	0.1	0.1	0.1	0.1
	Micronesia (Federated States of)	9.0	8.1	6.0	5.1
	Gabon	9.9	10.6	8.3	6.9
	United Kingdom	0.3	0.2	0.2	0.2
	Georgia	6.4	3.3	1.6	1.0
	Ghana	12.7	11.1	9.8	7.2
	Guinea	24.2	22.1	17.1	15.4
	Gambia	18.4	15.7	11.7	9.3
	Guinea-Bissau	29.8	28.8	19.7	15.8
	Equatorial Guinea	23.6	16.9	17.7	14.7
	Greece	0.2	0.3	0.1	0.2
	Grenada	1.5	1.3	0.4	0.8
	Guatemala	11.2	7.8	6.3	4.8
	Guyana	4.0	3.4	2.8	2.6
	Honduras	5.9	4.1	3.1	2.3
	Croatia	0.4	0.4	0.2	0.1
	Haiti	24.6	20.8	22.1	15.6
	Hungary	0.8	0.5	0.3	0.3
	Indonesia	10.3	7.8	5.9	4.6

Level	Country	Pneumonia deaths among children under-five			
		2000	2005	2010	2015
	India	17.3	13.5	10.2	7.1
	Ireland	0.2	0.2	0.2	0.1
	Iran (Islamic Republic of)	5.7	3.8	2.8	2.3
	Iraq	7.5	6.9	6.0	4.8
	Iceland	0.0	0.1	0.0	0.0
	Israel	0.1	0.2	0.1	0.1
	Italy	0.1	0.1	0.0	0.1
	Jamaica	1.2	1.2	1.2	1.0
	Jordan	3.2	2.4	1.9	1.6
	Japan	0.3	0.3	0.2	0.2
	Kazakhstan	8.0	5.4	3.1	1.9
	Kenya	18.2	13.0	10.2	6.9
	Kyrgyzstan	9.1	6.7	4.1	3.0
	Cambodia	23.8	13.6	7.2	4.7
	Kiribati	14.2	15.3	10.4	9.5
	Saint Kitts and Nevis	0.5	0.3	0.2	0.0
	Republic of Korea	0.4	0.2	0.1	0.1
	Kuwait	0.8	0.5	0.6	0.8
	Lao People's Democratic Republic	26.8	23.0	16.2	11.6
	Lebanon	2.2	1.2	0.7	0.5
	Liberia	23.5	19.5	15.7	11.5
	Libya	3.1	2.2	1.3	1.0
	Saint Lucia	0.7	1.1	1.1	0.8
	Sri Lanka	1.6	1.1	0.7	0.6
	Lesotho	18.4	20.9	16.6	15.3
	Lithuania	1.1	0.9	0.2	0.3
	Luxembourg	0.0	0.0	0.0	0.0
	Latvia	0.8	0.9	0.3	0.3

Level	Country	Pneumonia deaths among children under-five			
		2000	2005	2010	2015
	Morocco	8.7	6.3	4.1	3.1
	Monaco	0.0	0.0	0.3	0.1
	Republic of Moldova	8.3	5.4	3.1	2.5
	Madagascar	24.8	18.9	12.6	8.5
	Maldives	7.3	2.8	1.2	0.6
	Mexico	4.2	2.9	2.1	1.5
	Marshall Islands	6.9	6.8	6.3	6.0
	The former Yugoslav Republic of Macedonia	1.3	0.9	0.7	0.3
	Mali	35.1	27.7	23.1	14.8
	Malta	0.1	0.3	0.0	0.1
	Myanmar	15.3	13.2	11.2	8.2
	Montenegro	0.3	0.5	0.3	0.2
	Mongolia	12.4	7.5	4.3	3.3
	Mozambique	21.7	19.8	15.2	11.1
	Mauritania	21.4	21.5	17.6	12.3
	Mauritius	0.7	1.0	1.5	1.5
	Malawi	28.2	15.6	11.7	8.6
	Malaysia	0.9	0.6	0.5	0.3
	Namibia	11.6	11.3	9.3	8.3
	Niger	45.1	37.6	24.4	19.2
	Nigeria	28.8	26.1	24.9	19.0
	Nicaragua	7.3	5.4	4.4	3.6
	Niue	6.5	0.9	6.9	5.5
	Netherlands	0.1	0.1	0.1	0.1
	Norway	0.2	0.1	0.0	0.1
	Nepal	15.7	11.7	8.2	5.3
	Nauru	6.8	6.5	4.9	4.7
	New Zealand	0.4	0.4	0.4	0.4

Level	Country	Pneumonia deaths among children under-five			
		2000	2005	2010	2015
	Oman	1.9	1.1	0.8	0.7
	Pakistan	21.0	19.7	14.8	11.9
	Panama	2.5	2.9	3.0	2.3
	Peru	6.5	3.6	2.5	1.7
	Philippines	8.4	7.3	6.7	5.2
	Palau	2.6	2.1	2.1	1.2
	Papua New Guinea	12.3	13.2	10.9	9.2
	Poland	0.5	0.4	0.2	0.2
	Democratic People's Republic of Korea	11.9	5.6	5.6	3.7
	Portugal	0.3	0.2	0.1	0.1
	Paraguay	4.8	3.8	2.8	2.3
	Qatar	0.6	0.5	0.4	0.3
	Romania	8.2	5.8	4.0	3.2
	Russian Federation	3.1	1.8	1.0	0.7
	Rwanda	36.0	20.7	9.6	5.7
	Saudi Arabia	2.2	1.5	1.1	0.8
	Sudan	22.7	20.5	16.9	12.0
	Senegal	25.5	19.0	11.4	6.9
	Singapore	0.4	0.6	0.2	0.4
	Solomon Islands	5.4	5.5	5.6	5.1
	Sierra Leone	34.1	32.7	22.0	17.0
	El Salvador	6.0	3.9	2.6	2.1
	San Marino	0.3	0.1	0.1	0.1
	Somalia	34.9	39.9	39.4	32.9
	Serbia	0.7	0.6	0.3	0.3
	South Sudan	34.6	27.6	22.6	18.6
	Sao Tome and Principe	13.7	10.4	7.6	5.5
	Suriname	2.8	2.4	2.2	1.6

Level	Country	Pneumonia deaths among children under-five			
		2000	2005	2010	2015
	Slovakia	1.4	0.9	0.6	0.6
	Slovenia	0.0	0.0	0.1	0.1
	Sweden	0.1	0.1	0.1	0.1
	Swaziland	21.0	21.2	14.2	9.7
	Seychelles	0.9	0.8	0.6	0.7
	Syrian Arab Republic	3.3	2.2	1.5	1.0
	Chad	38.9	39.5	35.9	31.8
	Togo	15.2	13.3	11.5	11.8
	Thailand	3.0	2.0	1.5	1.1
	Tajikistan	21.1	14.1	9.1	7.6
	Turkmenistan	18.1	13.9	11.3	8.6
	Timor-Leste	24.6	18.4	15.3	10.6
	Tonga	2.3	1.8	1.9	1.8
	Trinidad and Tobago	0.9	2.1	1.8	1.6
	Tunisia	4.9	2.7	1.8	1.1
	Turkey	3.2	1.9	0.8	0.5
	Tuvalu	7.6	5.9	3.9	3.5
	United Republic of Tanzania	23.2	17.1	10.7	7.0
	Uganda	20.3	15.0	11.2	8.6
	Ukraine	2.8	1.6	1.0	0.8
	Uruguay	1.5	1.4	1.1	0.6
	United States of America	0.3	0.2	0.2	0.2
	Uzbekistan	12.1	9.8	6.8	5.8
	Saint Vincent and the Grenadines	0.7	0.5	0.8	1.0
	Venezuela (Bolivarian Republic of)	1.7	1.8	1.8	1.6
	Viet Nam	7.3	5.1	3.7	3.1
	Vanuatu	4.4	4.1	4.1	3.9
	Samoa	2.9	2.0	1.8	1.7

Level	Country	Pneumonia deaths among children under-five			
		2000	2005	2010	2015
	Yemen	22.0	14.5	10.0	6.5
	South Africa	11.0	10.1	8.5	7.0
	Zambia	27.6	17.4	14.4	9.8
	Zimbabwe	13.0	15.2	13.7	10.2

* World Bank region, # WHO region

Supplementary table 21: Pneumonia-specific mortality and number of deaths due to pneumonia among children under-five, assuming no Hib/PCV coverage, in 2000 and 2015

Country	Pneumonia mortality among children under-five (per 1,000 livebirths)		Pneumonia deaths among children under-five	
	**2000**	**2015**	**2000**	**2015**
**Afghanistan**	30.8	22.1	28942	22608
**Angola**	41.3	35.5	30484	38768
**Albania**	5.1	2.3	266	102
**Andorra**	0.1	0.1	0	0
**United Arab Emirates**	0.7	0.4	37	38
**Argentina**	1.6	1.3	1112	1011
**Armenia**	6.2	1.7	218	71
**Antigua and Barbuda**	0.0	0.3	0	0
**Australia**	0.2	0.2	46	47
**Austria**	0.1	0.1	8	5
**Azerbaijan**	15.0	4.9	1877	1143
**Burundi**	27.2	18.2	7522	8431
**Belgium**	0.1	0.1	9	12
**Benin**	25.3	19.7	7229	7424
**Burkina Faso**	19.0	16.2	9774	11097
**Bangladesh**	17.3	6.2	62632	19778
**Bulgaria**	4.4	2.5	290	152
**Bahrain**	0.3	0.2	4	5
**Bahamas**	4.1	2.3	21	13
**Bosnia and Herzegovina**	0.7	0.3	35	9
**Belarus**	2.0	0.3	181	36
**Belize**	3.1	1.6	24	13
**Bolivia (Plurinational State of)**	16.7	6.9	4236	1705
**Brazil**	4.5	2.1	17187	6888
**Barbados**	0.0	0.5	0	2
**Brunei Darussalam**	0.5	0.4	3	3
**Bhutan**	17.5	5.7	277	71
**Botswana**	7.0	7.3	330	423
**Central African Republic**	21.7	24.0	3135	3940
**Canada**	0.2	0.1	60	55
**Switzerland**	0.1	0.1	7	5
**Chile**	1.3	0.4	321	91
**China**	7.6	1.3	118092	22242
**Cote d'Ivoire**	14.0	14.7	9279	12209
**Cameroon**	25.6	19.1	16115	15655
**Democratic Republic of the Congo**	19.3	19.0	41587	59358
**Congo**	18.8	8.0	2202	1308
**Cook Islands**	1.8	0.5	1	0

Colombia	3.6	2.3	2911	1688
Comoros	18.3	12.7	373	333
Cabo Verde	6.0	3.9	76	43
Costa Rica	1.1	0.6	86	43
Cuba	0.6	0.7	92	82
Cyprus	0.4	0.1	5	1
Czech Republic	0.3	0.2	21	23
Germany	0.1	0.1	87	66
Djibouti	16.8	10.8	367	239
Dominica	0.5	1.0	0	1
Denmark	0.2	0.1	10	3
Dominican Republic	5.6	3.8	1164	823
Algeria	6.6	3.8	3723	3630
Ecuador	5.0	3.6	1573	1191
Egypt	9.1	3.0	15545	8375
Eritrea	19.4	10.8	2402	1799
Spain	0.1	0.1	55	26
Estonia	0.8	0.2	10	2
Ethiopia	29.6	12.5	86397	39430
Finland	0.1	0.1	5	5
Fiji	3.1	3.9	61	67
France	0.1	0.1	70	89
Micronesia (Federated States of)	10.1	6.3	33	15
Gabon	9.9	7.7	397	398
United Kingdom	0.3	0.2	210	176
Georgia	6.4	1.1	329	62
Ghana	12.7	10.3	7923	9267
Guinea	24.2	16.7	8732	7617
Gambia	21.4	13.3	1161	1098
Guinea-Bissau	29.8	17.9	1523	1165
Equatorial Guinea	23.6	15.2	485	435
Greece	0.3	0.2	27	26
Grenada	1.6	0.9	3	2
Guatemala	11.2	6.0	4456	2679
Guyana	4.0	3.5	74	60
Honduras	7.0	3.2	1360	524
Croatia	0.4	0.1	24	5
Haiti	24.6	16.8	6537	4325
Hungary	1.0	0.4	82	37
Indonesia	10.3	4.7	44917	25806
India	17.6	7.7	490274	193883
Ireland	0.2	0.1	14	6
Iran (Islamic Republic of)	5.7	2.3	7320	3098
Iraq	7.5	5.3	6354	6473
Iceland	0.0	0.0	0	0
Israel	0.1	0.1	17	18
Italy	0.1	0.1	59	35

Jamaica	1.2	1.2	68	46
Jordan	3.2	1.8	481	381
Japan	0.3	0.2	357	185
Kazakhstan	8.0	2.5	1687	961
Kenya	18.2	9.1	21276	13842
Kyrgyzstan	9.1	3.4	992	591
Cambodia	23.8	5.5	7405	1973
Kiribati	14.2	12.1	37	34
Saint Kitts and Nevis	0.5	0.0	1	0
Republic of Korea	0.4	0.1	188	39
Kuwait	0.9	1.2	42	84
Lao People's Democratic Republic	26.8	15.3	4314	2646
Lebanon	2.2	0.5	132	53
Liberia	23.5	13.5	2845	2040
Libya	3.1	1.3	369	162
Saint Lucia	0.7	0.9	2	3
Sri Lanka	1.6	0.7	550	224
Lesotho	18.4	17.9	1086	1119
Lithuania	1.1	0.3	38	10
Luxembourg	0.0	0.0	0	0
Latvia	0.9	0.4	16	7
Morocco	8.7	4.2	5299	3021
Monaco	0.0	0.1	0	0
Republic of Moldova	8.3	2.7	393	119
Madagascar	24.8	10.8	15968	8872
Maldives	7.3	0.7	45	5
Mexico	5.0	2.0	12390	4809
Marshall Islands	7.4	7.1	16	10
The former Yugoslav Republic of Macedonia	1.3	0.4	34	9
Mali	35.1	20.0	17781	14529
Malta	0.1	0.2	1	1
Myanmar	15.3	9.1	17745	8380
Montenegro	0.3	0.2	3	1
Mongolia	12.4	3.9	594	279
Mozambique	21.7	14.4	17254	15323
Mauritania	21.4	16.9	2162	2226
Mauritius	0.7	1.7	13	24
Malawi	28.2	12.0	13366	7551
Malaysia	0.9	0.4	484	219
Namibia	11.6	9.6	659	720
Niger	45.1	21.9	25830	20850
Nigeria	28.8	21.3	145231	148937
Nicaragua	8.4	5.3	1140	632
Niue	7.9	8.2	0	0
Netherlands	0.2	0.1	31	19
Norway	0.2	0.1	10	5
Nepal	15.7	6.1	12038	3355

Nauru	6.8	5.5	1	1
New Zealand	0.5	0.5	26	28
Oman	1.9	1.2	102	93
Pakistan	21.0	14.8	93923	79726
Panama	2.5	2.8	185	212
Peru	6.9	2.4	4231	1467
Philippines	8.4	5.9	19200	13910
Palau	3.0	1.6	1	1
Papua New Guinea	12.3	10.1	2300	2123
Poland	0.5	0.3	167	101
Democratic People's Republic of Korea	11.9	4.2	4637	1592
Portugal	0.3	0.1	32	8
Paraguay	4.8	3.1	705	419
Qatar	0.8	0.5	9	14
Romania	8.2	3.7	2186	607
Russian Federation	3.1	0.7	4632	1328
Rwanda	36.0	8.3	12710	2807
Saudi Arabia	2.2	1.2	1284	734
Sudan	22.7	16.8	24491	21733
Senegal	25.5	9.4	9729	5474
Singapore	0.4	0.4	16	20
Solomon Islands	5.4	5.8	75	97
Sierra Leone	34.1	23.6	5914	5154
El Salvador	6.0	2.9	835	304
San Marino	0.3	0.2	0	0
Somalia	34.9	34.9	12480	15567
Serbia	0.7	0.4	86	34
South Sudan	34.6	18.6	9754	8058
Sao Tome and Principe	13.7	7.8	72	49
Suriname	2.8	1.9	31	19
Slovakia	1.5	0.9	74	51
Slovenia	0.0	0.1	0	2
Sweden	0.1	0.2	7	20
Swaziland	21.0	13.5	696	494
Seychelles	0.9	0.9	1	1
Syrian Arab Republic	3.3	1.1	1749	453
Chad	38.9	34.2	15765	20707
Togo	15.2	14.7	2831	3675
Thailand	3.0	1.1	2651	843
Tajikistan	21.1	8.9	4031	2390
Turkmenistan	18.1	10.2	1621	1162
Timor-Leste	24.6	11.9	972	620
Tonga	2.3	2.2	6	6
Trinidad and Tobago	1.0	1.9	18	37
Tunisia	4.9	1.3	822	272
Turkey	3.2	0.7	4489	1045
Tuvalu	7.6	3.9	2	1

United Republic of Tanzania	23.2	10.0	31096	20593
Uganda	20.3	10.7	22161	17041
Ukraine	2.8	0.9	1111	368
Uruguay	1.7	0.9	92	45
United States of America	0.3	0.3	1168	1048
Uzbekistan	12.1	6.9	6539	4586
Saint Vincent and the Grenadines	0.7	1.2	2	2
Venezuela (Bolivarian Republic of)	1.8	1.8	1017	1068
Viet Nam	7.3	3.3	8603	5207
Vanuatu	4.4	4.3	26	29
Samoa	2.9	2.0	15	9
Yemen	22.0	8.7	14202	7154
South Africa	12.4	9.1	14564	9248
Zambia	27.6	13.2	12365	8140
Zimbabwe	13.0	14.3	5108	7819

Checklist of information that should be included in new reports of global health estimates

Item #	Checklist item	Reported on page #
**Objectives and funding**		
1	Define the indicator(s), populations (including age, sex, and geographic entities), and time period(s) for which estimates were made.	4-5
2	List the funding sources for the work.	13
**Data Inputs**		
For all data inputs from multiple sources that are synthesized as part of the study:		
3	Describe how the data were identified and how the data were accessed.	4-5, 17
4	Specify the inclusion and exclusion criteria. Identify all ad-hoc exclusions.	22
5	Provide information on all included data sources and their main characteristics. For each data source used, report reference information or contact name/institution, population represented, data collection method, year(s) of data collection, sex and age range, diagnostic criteria or measurement method, and sample size, as relevant.	Supplementary material p 7-16, 57-64, 67-69
6	Identify and describe any categories of input data that have potentially important biases (e.g., based on characteristics listed in item 5).	10-12
For data inputs that contribute to the analysis but were not synthesized as part of the study:		
7	Describe and give sources for any other data inputs.	5
For all data inputs:		
8	Provide all data inputs in a file format from which data can be efficiently extracted (e.g., a spreadsheet rather than a PDF), including all relevant meta-data listed in item 5. For any data inputs that cannot be shared because of ethical or legal reasons, such as third-party ownership, provide a contact name or the name of the institution that retains the right to the data.	We will provide a weblink to Edinburgh Datashare which will be active for public when paper is published.
**Data analysis**		
9	Provide a conceptual overview of the data analysis method. A diagram may be helpful.	4-6
10	Provide a detailed description of all steps of the analysis, including mathematical formulae. This description should cover, as relevant, data cleaning, data pre-processing, data adjustments and weighting of data sources, and mathematical or statistical model(s).	4-6, Supplementary material p 2-6
11	Describe how candidate models were evaluated and how the final model(s) were selected.	4-6
12	Provide the results of an evaluation of model performance, if done, as well as the results of any relevant sensitivity analysis.	101-105
13	Describe methods for calculating uncertainty of the estimates. State which sources of uncertainty were, and were not, accounted for in the uncertainty analysis.	5, 10

14	State how analytic or statistical source code used to generate estimates can be accessed.	Codes are on git hub. We will provide a weblink which will be active for public when paper is published.
Results and Discussion		
15	Provide published estimates in a file format from which data can be efficiently extracted.	We will provide a weblink to Edinburgh Datashare which will be active for public when paper is published.
16	Report a quantitative measure of the uncertainty of the estimates (e.g. uncertainty intervals).	6-9
17	Interpret results in light of existing evidence. If updating a previous set of estimates, describe the reasons for changes in estimates.	10
18	Discuss limitations of the estimates. Include a discussion of any modelling assumptions or data limitations that affect interpretation of the estimates.	10-12

This checklist should be used in conjunction with the GATHER statement and Explanation and Elaboration document, found on gather-statement.org

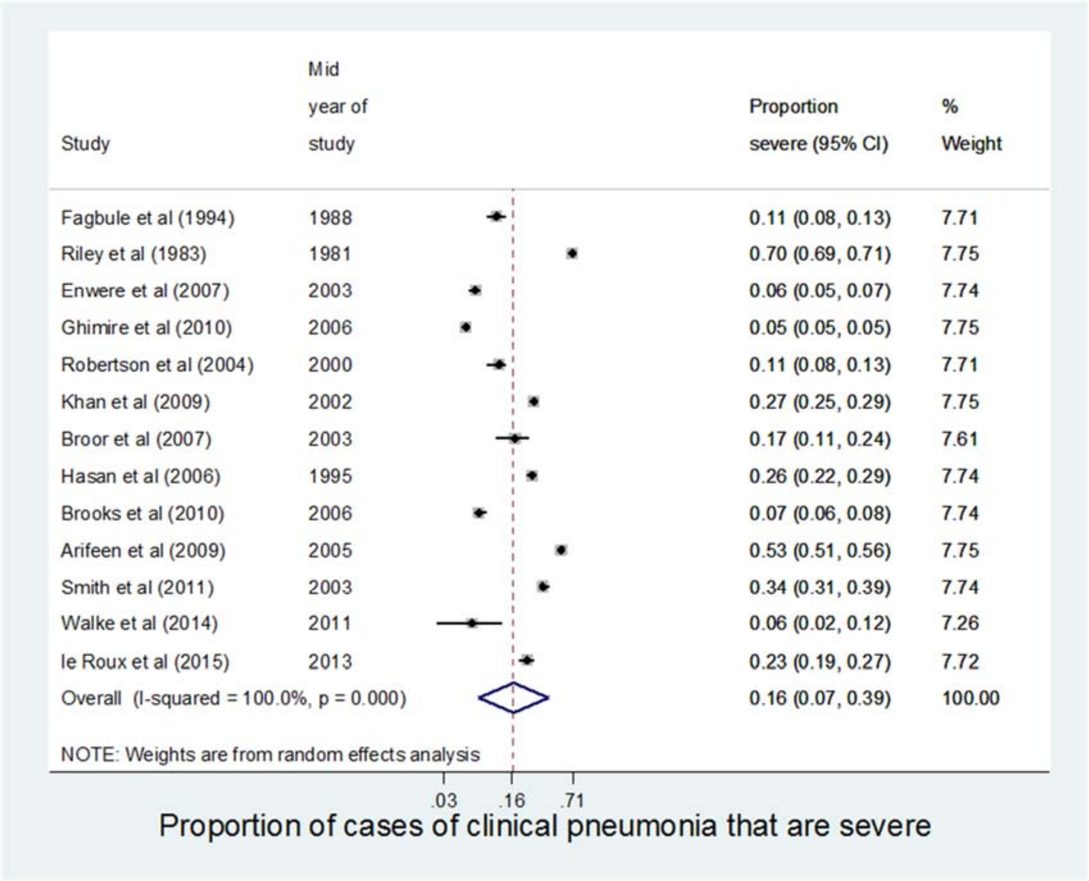

Supplementary figure 1: Studies reporting the proportion of children with clinical pneumonia having severe pneumonia (lower chest wall indrawing)

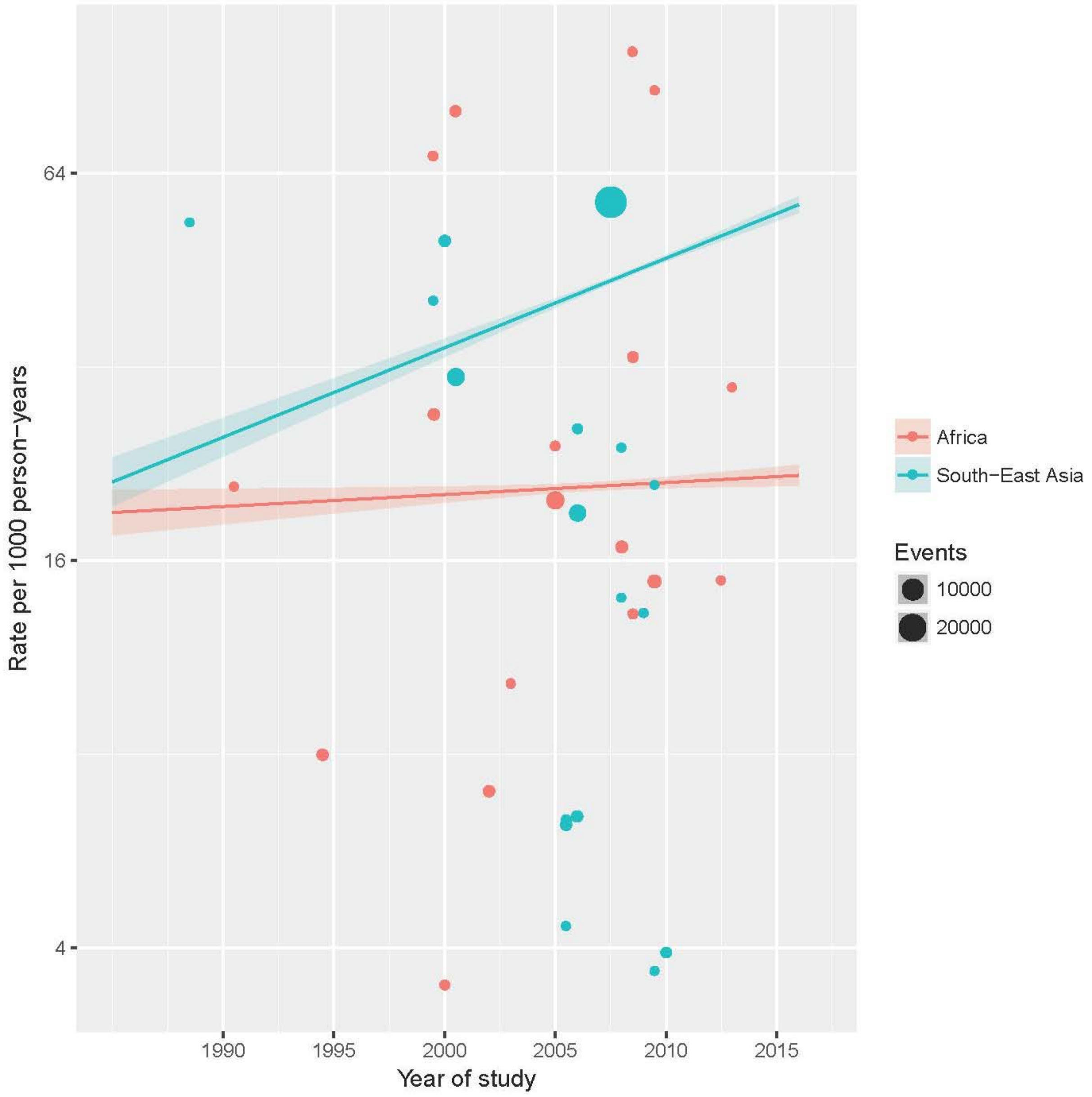

Supplementary figure 2: Hospitalisation rates for pneumonia in children younger than 5 years residing in South East Asia and Africa regions of WHO. The size of circle indicates number of pneumonia cases. The solid lines indicate trend and shaded areas indicate uncertainty range

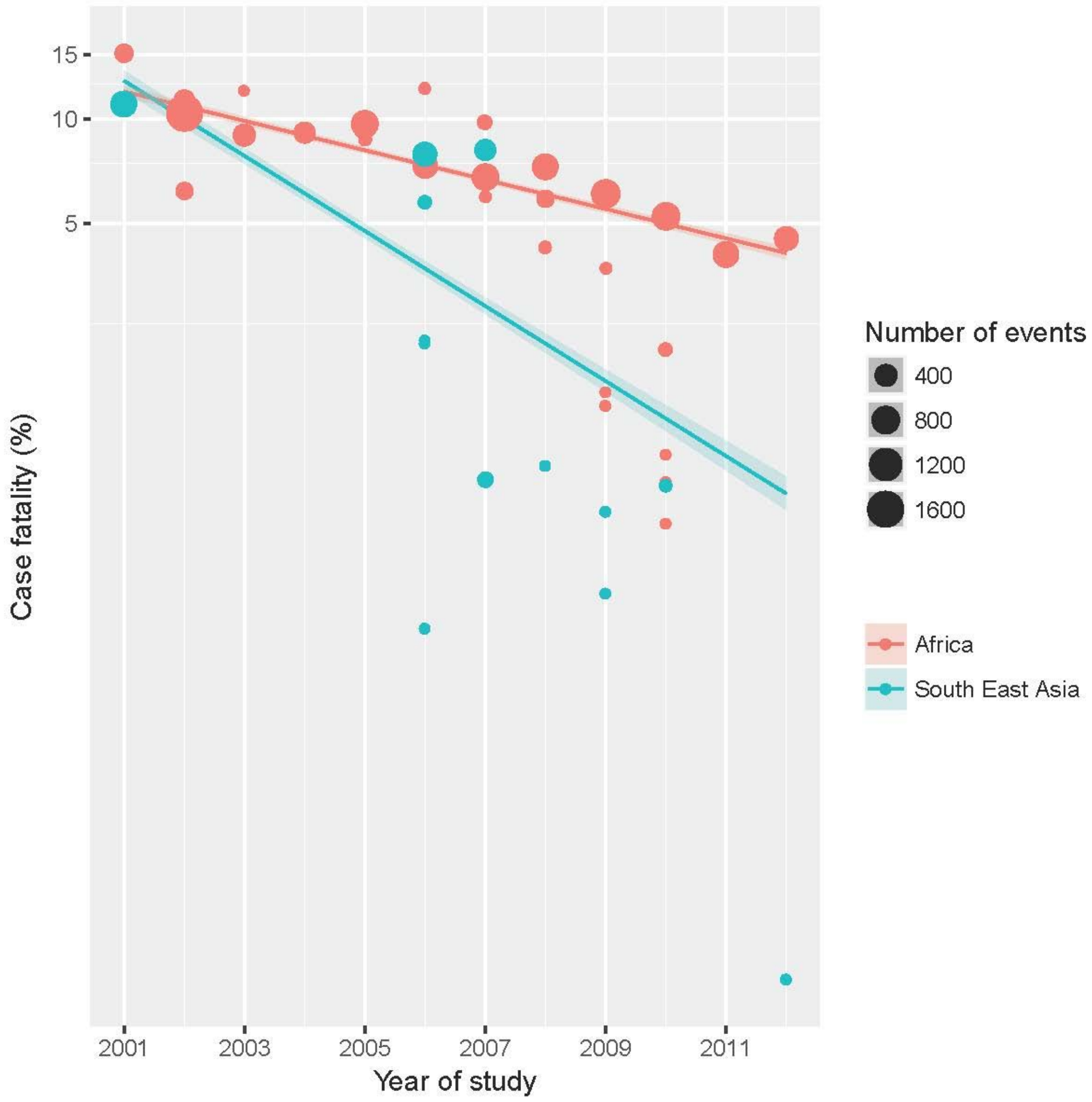

Supplementary figure 3: In-hospital case fatality ratio for child pneumonia in South-east Asia and Africa regions of WHO (2001-2012) on logit scale. The size of circle indicates number of pneumonia cases. The solid lines indicate trend and shaded areas indicate uncertainty range

References

1. Rothman KJ, Greenland S, Lash TL. Modern epidemiology. 3rd ed. Philadelphia: Lippincott Williams & Wilkins; 2008.

2. Oyejide C, Osinusi K. Acute respiratory tract infection in children in Idikan community, Ibadan, Nigeria: severity, risk factors, and frequency of occurrence. *Review of Infectious Diseases* 1990; **12**(Supplement 8): S1042-SI046.

3. Wafula E, Onyango F, Mirza W, et al. Epidemiology of acute respiratory tract infections among young children in Kenya. *Review of infectious diseases* 1990; **12**(Supplement 8): S1035-S8.

4. Campbell H, Lamont A, O'Neill K, et al. Assessment of clinical criteria for identification of severe acute lower respiratory tract infections in children. *The Lancet* 1989; **333**(8633): 297-9.

5. Afari E, SAKATOKU H, NAKANO T, et al. Acute respiratory infections in children under five in two rural communities in southern Ghana. *Japanese Journal of Tropical Medicine and Hygiene* 1991; **19**(3): 275-80.

6. Fagbule D, Parakoyi D, Spiegel R. Acute respiratory infections in Nigerian children: prospective cohort study of incidence and case management. *Journal of Tropical Pediatrics* 1994; **40**(5): 279-84.

7. Robertson SE, Roca A, Alonso P, et al. Respiratory syncytial virus infection: denominator-based studies in Indonesia, Mozambique, Nigeria and South Africa. *Bulletin of the World Health Organization* 2004; **82**(12): 914-22.

8. Enwere G, Cheung YB, Zaman S, et al. Epidemiology and clinical features of pneumonia according to radiographic findings in Gambian children. *Tropical medicine & international health* 2007; **12**(11): 1377-85.

9. le Roux DM, Myer L, Nicol MP, Zar HJ. Incidence and severity of childhood pneumonia in the first year of life in a South African birth cohort: the Drakenstein Child Health Study. *The Lancet Global Health* 2015; **3**(2): e95-e103.

10. Hortal M, Contera M, Mogdasy C, Russi JC. Acute respiratory infections in children from a deprived urban population from Uruguay. *Revista do Instituto de Medicina Tropical de Sao Paulo* 1994; **36**(1): 51-7.

11. Cruz JR, Pareja G, de Fernandez A, Peralta F, Caceres P, Cano F. Epidemiology of Acute Respiratory Tract Infections Among Guatemalan Ambulatory Preschool Cbildren. *Review of Infectious Diseases* 1990; **12**(Supplement 8): S1029-S34.

12. Borrero I, Fajardo L, Bedoya A, Zea A, Carmona F, de Borrero MF. Acute respiratory tract infections among a birth cohort of children from Cali, Colombia, who were studied through 17 months of age. *Review of Infectious Diseases* 1990; **12**(Supplement 8): S950-S6.

13. Smith KR, McCracken JP, Weber MW, et al. Effect of reduction in household air pollution on childhood pneumonia in Guatemala (RESPIRE): a randomised controlled trial. *The Lancet* 2011; **378**(9804): 1717-26.

14. Lanata CF. Incidence and evolution of pneumonia in children at the community level. *Respiratory infections in children Benguigui Y, Lopez Antuñano FJ, Schmunis G, Yunes J, eds Washington, DC: Pan American Health Organization* 1999: 59-83.

15. Khan AJ, Hussain H, Omer SB, et al. High incidence of childhood pneumonia at high altitudes in Pakistan: a longitudinal cohort study. *Bulletin of the World Health Organization* 2009; **87**(3): 193-9.

16. Ashraf S, Huque MH, Kenah E, Agboatwalla M, Luby SP. Effect of recent diarrhoeal episodes on risk of pneumonia in children under the age of 5 years in Karachi, Pakistan. *International journal of epidemiology* 2013; **42**(1): 194-200.

17. Datta N, Kumar V, Kumar L, Singhi S. Application of case management to the control of acute respiratory infections in low-birth-weight infants: a feasibility study. *Bulletin of the World Health Organization* 1987; **65**(1): 77.

18. Pandey MR, Sharma PR, Gubhaju BB, et al. Impact of a pilot acute respiratory infection (ARI) control programme in a rural community of the hill region of Nepal. *Annals of tropical paediatrics* 1989; **9**(4): 212-20.

19. Vathanophas K, Sangchai R, Raktham S, et al. A community-based study of acute respiratory tract infection in Thai children. *Review of Infectious Diseases* 1990; **12**(Supplement 8): S957-S65.

20. Reddaiah V, Kapoor SK. Acute respiratory infections in rural underfives. *Indian journal of pediatrics* 1988; **55**(3): 424-6.

21. Pandey MR, Daulaire N, Starbuck E, Houston R, McPherson K. Reduction in total under-five mortality in western Nepal through community-based antimicrobial treatment of pneumonia. *The Lancet* 1991; **338**(8773): 993-7.

22. Zaman K, Baqui A, Sack R, Bateman O, Chowdhury H, Black R. Acute respiratory infections in children: a community-based longitudinal study in rural Bangladesh. *Journal of Tropical Pediatrics* 1997; **43**(3): 133-7.

23. Singh M, Nayar S. Magnitude of acute respiratory infections in under five children. *The Journal of communicable diseases* 1996; **28**(4): 273-8.

24. Hasan K, Jolly P, Marquis G, et al. Viral etiology of pneumonia in a cohort of newborns till 24 months of age in Rural Mirzapur, Bangladesh. *Scandinavian journal of infectious diseases* 2006; **38**(8): 690-5.

25. Broor S, Parveen S, Bharaj P, et al. A prospective three-year cohort study of the epidemiology and virology of acute respiratory infections of children in rural India. *PloS one* 2007; **2**(6): e491.

26. Brooks WA, Goswami D, Rahman M, et al. Influenza is a major contributor to childhood pneumonia in a tropical developing country. *The Pediatric infectious disease journal* 2010; **29**(3): 216-21.

27. Arifeen SE, Saha SK, Rahman S, et al. Invasive pneumococcal disease among children in rural Bangladesh: results from a population-based surveillance. *Clinical Infectious Diseases* 2009; **48**(Supplement 2): S103-S13.

28. Walke SP, Das R, Acharya AS, Pemde HK. Incidence, pattern, and severity of acute respiratory infections among infants and toddlers of a peri-urban area of Delhi: A 12-month prospective study. *International Scholarly Research Notices* 2014; **2014**.

29. Lehmann D. Tari Research Unit: Final Report for the Southern Highlands Rural Development Project: Southern Highlands Rural Development Project; 1984.
30. Zhang Z, Gao L, Wang Z, Cao Y, Wu G, Zhu Z. Acute respiratory infections in Beijing children. Epidemiological studies at Dongguan Brigade. *Chin Med J (Engl)* 1986: 561-8.
31. Tupasi TE, de Leon LE, Lupisan S, et al. Patterns of acute respiratory tract infection in children: a longitudinal study in a depressed community in Metro Manila. *Review of Infectious Diseases* 1990; **12**(Supplement 8): S940-S9.
32. Smith TA, Lehmann D, Coakley C, Spooner V, Alpers MP. Relationships between growth and acute lower-respiratory infections in children aged less than 5 y in a highland population of Papua New Guinea. *The American journal of clinical nutrition* 1991; **53**(4): 963-70.
33. Hu YC, Lv WY. Analysis of childhood acute respiratory infection. *Shanghai Preventive Medicine* 1996; **8**(2).
34. Sun YF, Fang XQ, He HX, Zhu QZ, Wang Q, Chen HY. Analysis of surveilance results of acute respiratory infection in children aged 0-4 years. *Maternal And Child Health Care Of China* 1992; (05): 42-5+64.
35. Zhou P, Gu MF. Surveillance of childhood acute respiratory infection in 0-4 years. *Chinese Primary Health Care* 1994; (10): 30-1.
36. Mo JZ. Surveillance of 20867 children aged 0-4 years with ARI from rural areas in South of Jiangsu. *Chinese Primary Health Care* 1998; **12**(4).
37. Xie SM, Cheng L, Hou YJ, Cao SY, Yu Q. 3097 cases of acute respiratory infection in childhood aged 0-4 years. *Chongqing Medicine* 1993; **22**(6).
38. Lou LY, Cong GQ, Sun SX, Song YH, Li GL, Yang S. Analysis of characteristics of acute respiratory infection in children younger than 15 years old in rural areas in Heilongjiang Province. *Chinese Primary Health Care* 1995; **9**(2).
39. Zhou YY, Li ZH, Chen M, Huang GH. Seasonality analysis of pneumonia incidence in children younger than 5 years old from Guangzhou. *Chinese Journal Of Child Health Care* 2000; **8**(1): 35-6.
40. Cheng P. Deceasing childhood mortality from pneumonia via applying appropriate ARI management methods. *Jian Su Journal Of Preventive Medicine* 1996; **2**.
41. Chen W, Zhao MR, Zhao YY, Ma BJ. Analysis of acute respiratory infection in children from rural areas in Henan Province. *Chinese Rural Health* 1997; **25**(1).
42. Huang WH, Chen LN, Shi YB. Situation of acute respiratory infection in children less than 5 years in Licheng District. *Strait Journal Of Preventive Medicine* 1999; (02): 21.
43. Xu GL, Zheng JY, Li LX, Wei YH, Cai ZL. Surveillance of acute respiratory infection inunder five children from Huaning County in Yunnan Province. *Chinese Primary Health Care* 2000; **14**(6): 36-7.
44. Xie YL. Research of under five childhood mortality due to pneumonia from Hubei Province. *Hubei Journal of Preventive Medicine* 2003; **14**(4): 17-9.
45. Gao JY, Feng B, Li L. Research of establishing surveillance system to monitor childhood respiratory disease to decrease the mortality from pneumonia. *Maternal And Child Health Care Of China* 2004; **19**(16): 13-4.
46. Krishnan A, Amarchand R, Gupta V, et al. Epidemiology of acute respiratory infections in children - preliminary results of a cohort in a rural north Indian community. *BMC Infectious Diseases* 2015; **15 (1) (no pagination)**(462).
47. Nair H, Simões EAF, Rudan I, et al. Global and regional burden of hospital admissions for severe acute lower respiratory infections in young children in 2010: a systematic analysis. *Lancet* 2013; **381**: 1380-90.
48. Mulholland K, Hilton S, Adegbola R, et al. Randomised trial of Haemophilus influenzae type-b tetanus protein conjugate vaccine [corrected] for prevention of pneumonia and meningitis in Gambian infants.[Erratum appears in Lancet 1997 Aug 16;350(9076):524]. *Lancet* 1997; **349**(9060): 1191-7.
49. Madhi SA, Kuwanda L, Cutland C, Klugman KP. The impact of a 9-valent pneumococcal conjugate vaccine on the public health burden of pneumonia in HIV-infected and -uninfected children. *Clin Infect Dis* 2005; **40**(10): 1511-8.
50. Campbell JD, Sow SO, Levine MM, Kotloff KL. The causes of hospital admission and death among children in Bamako, Mali. *J Trop Pediatr* 2004; **50**(3): 158-63.
51. Tornheim JA, Manya AS, Oyando N, Kabaka S, Breiman RF, Feikin DR. The epidemiology of hospitalized pneumonia in rural Kenya: the potential of surveillance data in setting public health priorities. *Int J Infect Dis* 2007; **11**(6): 536-43.
52. Lowther SA, Shay DK, Holman RC, Clarke MJ, Kaufman SF, Anderson LJ. Bronchiolitis-associated hospitalizations among American Indian and Alaska Native children. *Pediatr Infect Dis J* 2000; **19**(1): 11-7.
53. Carroll KN, Gebretsadik T, Griffin MR, et al. Increasing burden and risk factors for bronchiolitis-related medical visits in infants enrolled in a state health care insurance plan. *Pediatrics* 2008; **122**(1): 58-64.
54. Grijalva CG, Nuorti JP, Zhu Y, Griffin MR. Increasing incidence of empyema complicating childhood community-acquired pneumonia in the United States. *Clin Infect Dis* 2010; **50**(6): 805-13.
55. Henrickson KJ, Hoover S, Kehl KS, Hua W. National disease burden of respiratory viruses detected in children by polymerase chain reaction. *Pediatr Infect Dis J* 2004; **23**(1 Suppl): S11-8.
56. Lee GE, Lorch SA, Sheffler-Collins S, Kronman MP, Shah SS. National hospitalization trends for pediatric pneumonia and associated complications. *Pediatrics* 2010; **126**(2): 204-13.
57. Peck AJ, Holman RC, Curns AT, et al. Lower respiratory tract infections among american Indian and Alaska Native children and the general population of U.S. Children. *Pediatr Infect Dis J* 2005; **24**(4): 342-51.
58. Weinberg GA, Hall CB, Iwane MK, et al. Parainfluenza virus infection of young children: estimates of the population-based burden of hospitalization. *J Pediatr* 2009; **154**(5): 694-9.

59. Yorita KL, Holman RC, Sejvar JJ, Steiner CA, Schonberger LB. Infectious disease hospitalizations among infants in the United States. *Pediatrics* 2008; **121**(2): 244-52.
60. Nizami SQ, Bhutta ZA, Hasan R. Incidence of acute respiratory infections in children 2 months to 5 years of age in periurban communities in Karachi, Pakistan. *JPMA J Pak Med Assoc* 2006; **56**(4): 163-7.
61. Owais A, Tikmani SS, Sultana S, et al. Incidence of pneumonia, bacteremia, and invasive pneumococcal disease in Pakistani children. *Trop Med Int Health* 2010; **15**(9): 1029-36.
62. Roxburgh CSD, Youngson GG, Townend JA, Turner SW. Trends in pneumonia and empyema in Scottish children in the past 25 years. *Arch Dis Child* 2008; **93**(4): 316-8.
63. Jokinen C, Heiskanen L, Juvonen H, et al. Incidence of community-acquired pneumonia in the population of four municipalities in eastern Finland. *Am J Epidemiol* 1993; **137**(9): 977-88.
64. Monge V, Gonzalez A. Hospital admissions for pneumonia in Spain. *Infection* 2001; **29**(1): 3-6.
65. Gil A, San-Martin M, Carrasco P, Gonzalez A. Epidemiology of pneumonia hospitalizations in Spain, 1995-1998. *J Infect* 2002; **44**(2): 84-7.
66. Garces-Sanchez MD, Diez-Domingo J, Ballester Sanz A, et al. Epidemiology of community-acquired pneumonia in children aged less than 5 years old in the Autonomous Community of Valencia (Spain). *An Pediatr (Barc)* 2005; **63**(2): 125-30.
67. Comes Castellano AM, Lluch Rodrigo JA, Portero Alonso A, Pastor Villalba E, Sanz Valero M. Development of the incidence of pneumonia in the autonomous community of Valencia throughout the 1995-2001 period. A retrospective study. *Anales de Medicina Interna* 2005; **22**(3): 118-23.
68. Vicente D, Montes M, Cilla G, Perez-Yarza EG, Perez-Trallero E. Hospitalization for respiratory syncytial virus in the paediatric population in Spain. *Epidemiol Infect* 2003; **131**(2): 867-72.
69. Weigl JAI, Puppe W, Belke O, Neususs J, Bagci F, Schmitt HJ. The descriptive epidemiology of severe lower respiratory tract infections in children in Kiel, Germany. *Klin Padiatr* 2005; **217**(5): 259-67.
70. van Gageldonk-Lafeber AB, Bogaerts MAH, Verheij RA, van der Sande MAB. Time trends in primary-care morbidity, hospitalization and mortality due to pneumonia. *Epidemiol Infect* 2009; **137**(10): 1472-8.
71. Forster J, Ihorst G, Rieger CHL, et al. Prospective population-based study of viral lower respiratory tract infections in children under 3 years of age (the PRI.DE study). *Eur J Pediatr* 2004; **163**(12): 709-16.
72. Ansaldi F, Sticchi L, Durando P, et al. Decline in pneumonia and acute otitis media after the introduction of childhood pneumococcal vaccination in Liguria, Italy.[Erratum appears in J Int Med Res. 2009 Mar-Apr;37(2):594]. *J Int Med Res* 2008; **36**(6): 1255-60.
73. Cilla G, Onate E, Perez-Yarza EG, Montes M, Vicente D, Perez-Trallero E. Hospitalization rates for human metapneumovirus infection among 0- to 3-year-olds in Gipuzkoa (Basque Country), Spain. *Epidemiol Infect* 2009; **137**(1): 66-72.
74. Che D, Caillere N, Brosset P, Vallejo C, Josseran L. Burden of infant bronchiolitis: data from a hospital network. *Epidemiol Infect* 2010; **138**(4): 573-5.
75. Zaman K, Baqui AH, Yunus M, et al. Acute respiratory infections in children: a community-based longitudinal study in rural Bangladesh. *J Trop Pediatr* 1997; **43**(3): 133-7.
76. Brooks WA, Santosham M, Naheed A, et al. Effect of weekly zinc supplements on incidence of pneumonia and diarrhoea in children younger than 2 years in an urban, low-income population in Bangladesh: randomised controlled trial. *Lancet* 2005; **366**(9490): 999-1004.
77. Baqui AH, Rahman M, Zaman K, et al. A population-based study of hospital admission incidence rate and bacterial aetiology of acute lower respiratory infections in children aged less than five years in Bangladesh. *J Health Popul Nutr* 2007; **25**(2): 179-88.
78. Chandyo RK, Shrestha PS, Valentiner-Branth P, et al. Two weeks of zinc administration to Nepalese children with pneumonia does not reduce the incidence of pneumonia or diarrhea during the next six months. *J Nutr* 2010; **140**(9): 1677-82.
79. Shah AS, Knoll MD, Sharma PR, et al. Invasive pneumococcal disease in Kanti Children's Hospital, Nepal, as observed by the South Asian Pneumococcal Alliance network. *Clin Infect Dis* 2009; **48** (Suppl 2): S123-8.
80. Hasan R, Rhodes J, Thamthitiwat S, et al. Incidence and etiology of acute lower respiratory tract infections in hospitalized children younger than 5 years in rural Thailand. *Pediatr Infect Dis J* 2014; **33**(2): e45-e52.
81. Williams EJ, Thorson S, Maskey M, et al. Hospital-based surveillance of invasive pneumococcal disease among young children in urban Nepal. *Clin Infect Dis* 2009; **48** (Suppl 2): S114-22.
82. Shah AS, Nisarga R, Ravi Kumar KL, Hubler R, Herrera G, Kilgore PE. Establishment of population-based surveillance for invasive pneumococcal disease in Bangalore, India. *Indian J Med Sci* 2009; **63**(11): 498-507.
83. Tupasi TE, de Leon LE, Lupisan S, et al. Patterns of acute respiratory tract infection in children: a longitudinal study in a depressed community in Metro Manila. *Rev Infect Dis* 1990; **12** (Suppl 8): S940-9.
84. Williams P, Gracey M, Smith P. Hospitalization of aboriginal and non-aboriginal patients for respiratory tract diseases in Western Australia, 1988-1993. *Int J Epidemiol* 1997; **26**(4): 797-805.
85. Sun YF, Fang XQ, He HX, Zhu QZ, Wang Q, Chen HY. Analysis of acute respiratory infections surveillance in children aged 0-4 years old (0-4岁儿童急性呼吸道感染监测结果分析). *Chinese Maternal and Child Health (中国妇幼保健)* 1992; **7**(5).
86. Hu YC, Lu WY. Effect of acute respiratory infections management in children (小儿急性呼吸道感染管理效果分析). *Shanghai Journal of Preventive Medicine (上海预防医学杂志)* 1996; **8**(2).

87. Wang L, Dong SP, Zhao GZ, Li JS. Promoting standard case management of acute respiratory infections to reduce mortality in children aged 0-4 years old (推广儿童急性呼吸道感染标准病例管理降低0-4岁儿童死亡率). *Chinese Journal of Primary Health Care (中国初级卫生保健)* 1997; **11**(3).
88. Liu Q, Fu P, Zhao S, Zou SH. Analysis of acute respiratory infections surveillance in children aged 0-4 years old in Qingdao (青岛市0-4岁儿童急性呼吸道感染监测结果分析). *Acta Academiae Medicinae Qingdao (青岛医学院学报)* 1994; **30**(3).
89. Xie SM, Chen L, Hou YJ, Zhen SY, Yu Q. Analysis of acute respiratory infections surveillance in 3097 children aged 0-4 years old (0-4岁小儿急性呼吸道感染监测3097例分析). *Chongqing Medical Journal 重庆医学* 1993; **22**(6).
90. Lou LY, Cong GQ, Sun SX, Song YH, Li GL, Yang S. Analysis of acute respiratory infections surveillance in children under 5 years in rural Heilongjiang (黑龙江省农村5岁以下儿童急性呼吸道感染监测分析). *Chinese Journal of Primary Health Care (中国初级卫生保健)* 1995; **9**(2).
91. Grant CC, Scragg R, Tan D, Pati A, Aickin R, Yee RL. Hospitalization for pneumonia in children in Auckland, New Zealand. *J Paediatr Child Health* 1998; **34**(4): 355-9.
92. Chi XX, Chen X, Ouyang Y, Xue XL. Preliminary analysis of acute respiratory infections surveillance in children under 5 years in Fujian (福建省ARI项目县5岁以下儿童监测结果初步分析). *Strait Journal of Preventive Medicine (海峡预防医学杂志)* 1996; **2**(3).
93. Chen W, Zhao MR, Zhao YY, Ma BJ. Analysis of acute respiratory infections surveillance in children in rural Henan (河南农村婴幼儿急性呼吸道感染监测结果分析). *Chinese Journal of Rural Medicine (中国农村医学)* 1997; **25**(4).
94. Chen P. Effect of acute respiratory infections management to reduce mortality in children with pneumonia (运用ARI管理适宜技术降低婴幼儿肺炎死亡率). *Jiangsu Journal of Preventive Medicine (江苏预防医学)* 1996; **2**.
95. Mo JZ. Analysis of acute respiratory infections surveillance in 20867 children aged 0-4 years old in Southern Jiangsu (苏南农村20867名0-4岁儿童ARI监测研究). *Chinese Journal of Primary Health Care (中国初级卫生保健)* 1998; **12**(4).
96. Huang WH, Chen LN, Shi LB. Analysis of acute respiratory infections surveillance in children aged 0-4 years old in Licheng (鲤城区0-4岁儿童急性呼吸道感染监测结果分析). *Strait Journal of Preventive Medicine (海峡预防医学杂志)* 1999; **5**(2).
97. Xu GL, Zheng JY, Li LX, Wei YH, Cai ZL. Analysis of acute respiratory infections monitoring in children under 5 years in Huaning, Yunnan (云南省华宁县5岁以下儿童急性呼吸道感染监测分析). *Maternal and Child Health (妇幼保健)* 2000; **14**(6).
98. Gao JY, Feng B, Li L. Establishing respiratory monitoring network to reduce pneumonia mortality in children (建立儿童呼吸监测网控制肺炎降低肺炎死亡率的研究). *Chinese Journal of Maternal and Child Health (中国妇幼保健)* 2004; **19**(8).
99. Qu JS, Li L, Wang GY. The influence of acute respiratory infection administration on mortality of pnemonia in children under five years old (急性呼吸道感染管理对5岁以下儿童肺炎死亡率的影响). *Qilu Journal of Medicine (齐鲁医学杂志)* 2009; **24**(1).
100. Moore HC, de Klerk N, Richmond P, Lehmann D. A retrospective population-based cohort study identifying target areas for prevention of acute lower respiratory infections in children. *BMC Public Health* 2010; **10**: 757.
101. Russell FM, Fakakovi T, Paasi S, Ika A, Mulholland EK. Reduction of meningitis and impact on under-5 pneumonia after introducing the Hib vaccine in the Kingdom of Tonga. *Ann Trop Paediatr* 2009; **29**(2): 111-7.
102. Ho P-L, Chiu SS, Chow FKH, Mak GC, Lau YL. Pediatric hospitalization for pneumococcal diseases preventable by 7-valent pneumococcal conjugate vaccine in Hong Kong. *Vaccine* 2007; **25**(39-40): 6837-41.
103. Magree HC, Russell FM, Sa'aga R, et al. Chest X-ray-confirmed pneumonia in children in Fiji. *Bull World Health Organ* 2005; **83**(6): 427-33.
104. Anh DD, Kilgore PE, Slack MP, et al. Surveillance of pneumococcal-associated disease among hospitalized children in Khanh Hoa Province, Vietnam. *Clin Infect Dis* 2009; **48** (Suppl 2): S57-64.
105. Yoshida LM, Suzuki M, Yamamoto T, et al. Viral pathogens associated with acute respiratory infections in central vietnamese children. *Pediatr Infect Dis J* 2010; **29**(1): 75-7.
106. Kosai H, Tamaki R, Saito M, et al. Incidence and Risk Factors of Childhood Pneumonia-Like Episodes in Biliran Island, Philippines--A Community-Based Study. *PLoS ONE [Electronic Resource]* 2015; **10**(5): e0125009.
107. Onyango CO, Njeru R, Kazungu S, et al. Influenza Surveillance Among Children With Pneumonia Admitted to a District Hospital in Coastal Kenya, 2007-2010. *J Infect Dis* 2012; **206**: S61-S7.
108. Strachan RE, Snelling TL, Jaffe A. Increased paediatric hospitalizations for empyema in Australia after introduction of the 7-valent pneumococcal conjugate vaccine. *Bull World Health Organ* 2013; **91**(3): 167-73.
109. Greenbaum AH, Chen J, Reed C, et al. Hospitalizations for severe lower respiratory tract infections. *Pediatrics* 2014; **134**(3): 546-54.
110. Nisarga R, Premalatha R, Shivananda, et al. Hospital-based surveillance of invasive pneumococcal disease and pneumonia in South Bangalore, India. *Indian Pediatrics* 2015; **52**(3): 205-11.
111. Poling J, Kelly L, Chan C, Fisman D, Ulanova M. Hospital admission for community-acquired pneumonia in a First Nations population. *Can J Rural Med* 2014; **19**(4): 135-41.
112. Capeding MR, Bravo L, Santos J, et al. Prospective Surveillance Study of Invasive Pneumococcal Disease Among Urban Children in the Philippines. *Pediatr Infect Dis J* 2013; **32**(10): E383-E9.

113. Ayieko P, Okiro EA, Edwards T, Nyamai R, English M. Variations in mortality in children admitted with pneumonia to Kenyan hospitals. *PLoS One* 2012; **7**(11): e47622.
114. Moore HC, Lehmann D, de Klerk N, Jacoby P, Richmond PC. Reduction in disparity for pneumonia hospitalisations between Australian indigenous and non-Indigenous children. *J Epidemiol Commun H* 2012; **66**(6): 489-94.
115. Ngoy BB, Zachariah R, Hinderaker SG, et al. Paediatric in-patient care in a conflict-torn region of Somalia: are hospital outcomes of acceptable quality? *Public Health Action* 2013; **3**(2): 125-7.
116. Ramachandran P, Nedunchelian K, Vengatesan A, Suresh S. Risk factors for mortality in community-acquired pneumonia among children aged 1-59 months admitted in a referral hospital. *Indian Pediatrics* 2012; **49**(11): 889-95.
117. Ramakrishna B, Graham SM, Phiri A, Mankhambo L, Duke T. Lactate as a predictor of mortality in Malawian children with WHO-defined pneumonia. *Archives of Disease in Childhood* 2012; **97**(4): 336-U.
118. Webb C, Ngama M, Ngatia A, et al. Treatment Failure Among Kenyan Children With Severe Pneumonia-A Cohort Study. *Pediatr Infect Dis J* 2012; **31**(9): E152-E7.
119. Lazzerini M, Seward N, Lufesi N, et al. Mortality and its risk factors in Malawian children admitted to hospital with clinical pneumonia, 2001-12: a retrospective observational study. *Lancet Global Health* 2016; **4**(1): e57-e68.
120. Bonsignori F, Chiappini E, Orlandini E, et al. Hospitalization rates of complicated pneumococcal community-acquired pneumonia is increasing in Tuscan children. *Int J Immunopathol Pharmacol* 2013; **26**(4): 995-1005.
121. Wysocki J, Sluzewski W, Gutterman E, Jouve S, Moscariello M, Balter I. Active hospital-based surveillance of invasive pneumococcal disease and clinical pneumonia in infants and young children in two Polish counties. *Archives of Medical Science* 2016; **12**(3): 629-38.
122. Enarson PM, Gie RP, Mwansambo CC, et al. Potentially modifiable factors associated with death of infants and children with severe pneumonia routinely managed in district hospitals in Malawi. *PLoS ONE* 2015; **10 (8) (no pagination)**(e0133365).
123. Lanaspa M, O'Callaghan-Gordo C, Machevo S, et al. High prevalence of Pneumocystis jirovecii pneumonia among Mozambican children <5 years of age admitted to hospital with clinical severe pneumonia. *Clinical Microbiology & Infection* 2015; **21**(11): 1018.e9-.e15.
124. Saha S, Hasan M, Kim L, et al. Epidemiology and risk factors for pneumonia severity and mortality in Bangladeshi children <5 years of age before 10-valent pneumococcal conjugate vaccine introduction. *BMC Public Health* 2016; **16**(1): 1233.
